# Accurate Molecular Size Determination by Diffusion Ordered NMR Spectroscopy Based on an Improved Diffusion Model

**DOI:** 10.1002/chem.71471

**Published:** 2026-07-24

**Authors:** Christian Urbank, Lisa Vondung

**Affiliations:** ^1^ Department of Chemistry University of Hamburg Hamburg Germany; ^2^ Laboratory For Zero‐Carbon Energy Institute of Integrated Research Institute of Science Tokyo Meguro‐ku Tokyo Japan

**Keywords:** diffusion, DOSY, NMR spectroscopy, uranium, uranyl complexes

## Abstract

Using the molecular volume, a semiempirical model for diffusion was developed, which allows an accurate interpretation of diffusion coefficients obtained by diffusion‐ordered spectroscopy (DOSY) NMR. While the measurement of diffusion coefficients with DOSY NMR is well‐established, correlating the obtained diffusion coefficients with molecular descriptors is difficult. Various models for small molecule diffusion have been developed in the past for organic, organometallic or coordination compounds, but none are applicable for all different substance classes. In this work we first analyze the different models using a dataset of 95 molecules and show that models relying on the common assumption of a spherical molecule, and with it the loss of information through the introduction of a radius‐parameter, produce large errors. Our new model therefore uses the molecular volume as molecular descriptor. This new model describes the diffusion of organic, organometallic and coordination compounds independent of molecular weight or density within a 2*σ* error range of 12%. The utility of the new model is demonstrated in the analysis of a range of uranyl complexes, where errors of −2% to 8% are obtained.

## Graphical abstract

A semi‐empirical *Stokes‐Einstein*‐based model for describing the diffusion of organic, organometallic and coordination compounds is developed for the interpretation of diffusion constants obtained by diffusion‐ordered NMR spectroscopy (DOSY). This model uses the molecular volume as a descriptor and shows a high accuracy, enabling application to challenging compounds, such as heavy‐metal complexes, where distinction between chloride and nitrate ligands is possible.

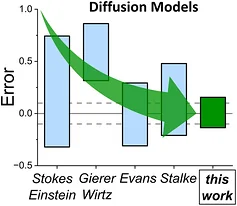

## 1 Introduction

The characterization of aggregational states of molecular compounds in solution is of interest to various fields ranging from macromolecular polymers and proteins via metal clusters and complexes to small organic molecules [1, 2, 3]. In coordination chemistry for example, it is often of interest to determine the number of ligands as well as potential coordination of solvent molecules in solution, as the structure in solution often differs from the solid state. For highly symmetric compounds, standard 1D and 2D NMR methods are often unsuitable for this task. *Diffusion ordered spectroscopy* (DOSY) NMR, however, enables the determination of the self‐diffusion coefficient *D*, which correlates with the size of the molecule or aggregate (expressed through the hydrodynamic radius *r*_H_), as shown in the *Stokes‐Einstein* equation:

(1) $$\begin{array}{c} D=\frac{k_{B}T}{6\pi \eta r_{H}}\; \end{array}$$

where k_B_ is the *Boltzmann* constant, *T* the temperature and *η* the dynamic viscosity. Extracting information on the composition of a compound from the experimentally obtained diffusion coefficient is therefore an attractive, but challenging task [3]. While diffusion NMR methods have been developed to robust techniques since the early 1990s, the qualitative analysis of the obtained spectra is still limited. Since the *Stokes‐Einstein* equation is not applicable to smaller molecules which are of similar size as the solvent molecules (<1000 g/mol), different models were developed previously linking the self‐diffusion constant *D* with a property characterizing the analyte molecule [3].

Several approaches based on modifications of the *Stokes‐Einstein* equation were developed in the past, but none of them are applicable to a wide range of molecules [4, 5]. Adjustments of these theories in the form of semiempirical models, new purely empirical models and calibrations were shown to circumvent major deviations and achieve high accuracies [6, 7, 8, 9, 10, 11, 12], but they are still limited in their validity to a close range in the vicinity of the calibration data set [13]. One of the reasons for the limited validity can be found in the choice of the parameter describing the molecule. The various models in use today are based on different molecular parameters and assumptions, which can easily lead to errors and misunderstandings in the application of these models (see Supporting Information for a discussion of the models compared in this work).

A limiting factor in the development of more universal diffusion models was the availability of comparable diffusion datasets which can be used to study diffusion behaviour over a wide range of molecular sizes, weights, and shapes independently of the spectrometer, temperature and concentration. Due to these dependencies, data obtained under different conditions could not be compared directly. In 2015, *Stalke* and coworkers established a straightforward normalization method (Equation 2) that allows for the comparison of diffusion data generated by DOSY NMR [13]:

(2) $$\begin{array}{c} log\;D_{x,\mathrm{norm}}=log\;D_{\mathrm{ref},\mathrm{fix}}-log\;D_{\mathrm{ref}}+log\;D_{x}\; \end{array}$$

with *D*_x, norm_ the normalized diffusion value of analyte x (in the following denoted as *D*), *D*_ref, fix_ the fixed value of the reference, *D*_ref_ the measured diffusion coefficient of the reference, and *D*_x_ the measured diffusion coefficient of analyte x.

When investigating a range of uranyl (UO_2_^2+^) complexes with various ligands with DOSY NMR, we noticed large deviations between the different widely used methods. We therefore wanted to gain insights into the validity of the most common diffusion models for smaller molecules through a direct comparison. In this work, we thus first compiled a dataset of *Stalke* normalized literature data for *D* enabling a direct comparison of commonly used diffusion models. Building on the gained insights, we then introduce a new model of diffusion based on the *van der Waals* volume of a molecule *V*_vdW_ which gives high accuracy over a wide range without the need for an additional correction. Finally, we demonstrate the applicability of our model in the investigation of uranyl complexes in solution.

## 2 Results and Discussion

The dataset used for evaluating the different diffusion models contains 95 molecules for which diffusion coefficients with the normalization by *Stalke* from DOSY NMR data in THF‐*d*_8_ (77 data points), C_6_D_6_ (55 data points) and/or toluene‐*d*_8_ (27 data points) were available. Since only smaller numbers of data points were available for other solvents, we restricted our investigations to these three solvents. Organic, organometallic, as well as transition metal complexes were included in the data set [9, 13, 14, 15, 16, 17, 18]. The viscosity values required for all calculations are adjusted using the formalism of *Holz *et al. [19]. The formalism for calculating the relative deviation and further details on the data set can be found in the Supporting Information.

### 2.1 *r*_H_—A Useful Parameter?

When the *Stokes*‐*Einstein* equation is used directly, it is usually assumed that the hydrodynamic radius *r*_H_ is related to the geometry of the molecule and describes a virtual sphere around the molecule.

As far as we are aware, this assumption has never been tested in detail for small molecules. A radius parameter that directly characterizes the size of a particle of any shape is the radius of gyration *r*_g_. Put simply, *r*_g_ describes the average distance of the atoms in the molecule from its centre of mass [20]. In a model of diffusion for proteins derived previously by *Tyn* and *Gusek*, *r*_g_ was used successfully in describing larger biochemical molecules [21].

We thus calculated *r*_g_ for the data set using *OVITO Basic 3.10.2* [22]. Plotting the relative deviation of *r*_H_ from *r*_g_ as obtained from the *Stokes‐Einstein* equation against the normalized *D* values shows that *r*_H_ generally overestimates the size of the molecule (Figure 1). This confirms the findings of *Evans* et al. [8]. While *Tyn* and *Gusek* observed a *2σ* error of ±20.8% for the correlation between *r*_g_ and *r*_H_ in their study of larger biomolecules [21], much larger *2σ* errors of 42% (THF‐*d*_8_), 43% (toluene‐*d*_8_), and 29% (C_6_D_6_) were obtained in our analysis, which clearly shows that the *Stokes‐Einstein* equation (Equation 1) alone is unsuitable for the analysis of diffusion of small molecules, if interpreted in this way [23].

**FIGURE 1:**
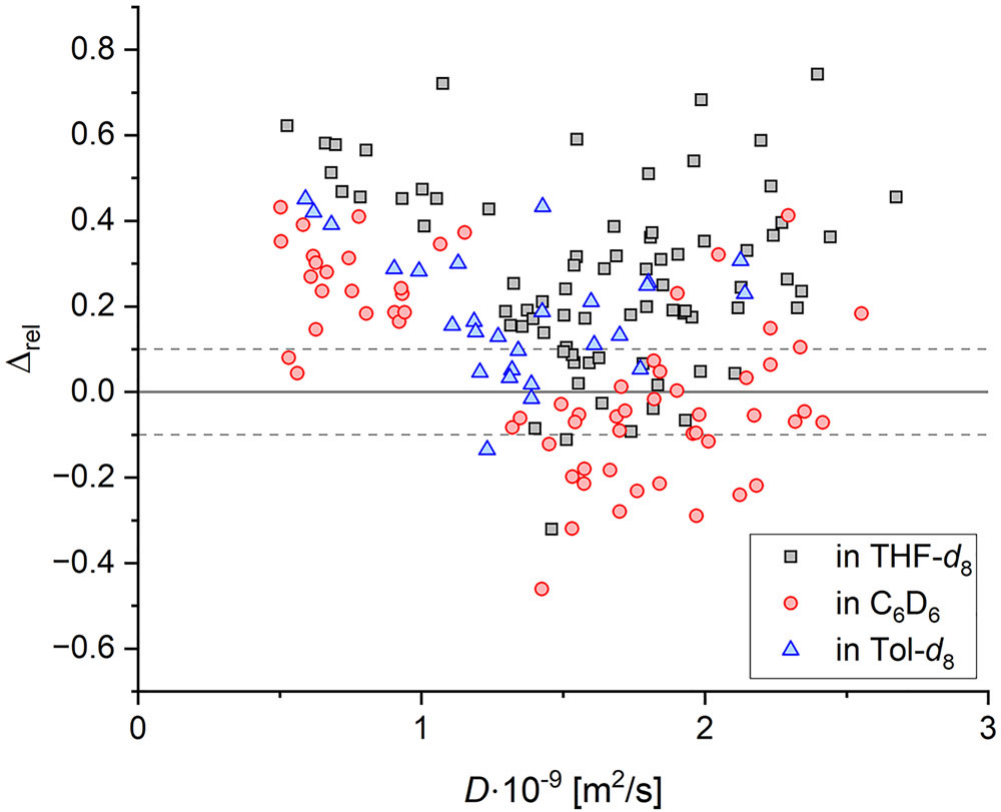
Plot of the relative deviation of *r*_H_ from *r*_g_ against the normalized diffusion coefficients *D*. Δ_rel_ was calculated using: $\Delta_{\mathrm{rel}}=\frac{r_{H}-r_{g}}{r_{g}}$ = (x*_r_*_H_‐x*_r_*_g_)/x*_r_*_g_.

Additionally, the question arises if a length parameter in general is suitable in diffusion modelling. For tackling this question, we obtained empirical power‐laws ($D=a\cdot {\left({r_{g}}^{-1}\right)}^{b}$) from r_g_^−1^ versus *D* plots (Figure S24). The thus generated models are characterized by *2σ* errors of 39% in THF‐*d*_8_, 56% in Tol‐*d*_8_, and 27% in C_6_D_6_ (see Table S13). As these empirical models are more flexible in comparison to the rigid *Stokes*‐*Einstein* relation, they show higher accuracy and precision within the given data set. Nevertheless, the errors are still too large for these models to be analytically useful. Thus, using a length parameter for this purpose does not seem to be advisable.

### 2.2 *Gierer*‐*Wirtz* and Volume Question

According to the microfriction theory of *Gierer* and *Wirtz* the error of the *Stokes*‐*Einstein* equation arises from the continuum assumption of the *Stokes* equation. They tried to circumvent this problem via the introduction of a microfriction correction factor *f*_t_ [5], an approach that was later used in a semiempirical approach by *Chen* and *Chen* [7].

The biggest challenge of the *Gierer‐Wirtz* model, and models based on it, is the generation of a reliable measure for the geometry of a given molecule. At the time of its derivation, *Gierer* and *Wirtz* had to accept a certain level of uncertainty introduced by their strategy in describing molecular geometries. Due to developments in computational methods and crystallography, we are now in a position to use descriptors which are more accurate, such as the volume or surface area.

For testing which descriptor for the molecular geometry correlates well with the experimentally observed *D*, we first fitted power laws in the form of $D=a\cdot {\left(V^{-1}\right)}^{b}$ on plots of experimental *Stalke* normalized values for *D* versus molecular volumes *V* ^−1^. The molecular volume parameters in this analysis were derived from (a) the sum of the covalence volumes derived from the covalence radii of the bonded atoms *V*_cov_ [24], (b) the sum of the *van der Waals* volumes *V*_∑vdW_ derived from the *van der Waals* radii of the bound atoms [25], (c) the *van der Waals* volume of the molecules *V*_vdW_ calculated using the software MoloVol 1.1.1 [26], and (d) the volume of the molecule *V*_C_ generated by the Connolly method, where the size of the spherical probe was derived from the *V*_vdW_ of the solvent [26]. The individual plots, fitting parameters and deviations from the experimental data (mean errors and double standard deviations) for the different volume parameters in THF‐*d*_8_, Tol‐*d*_8_, and C_6_D_6_ can be found in Figures S19–S21, and Tables S10–S12.The quality of the model based on the different volume descriptors was evaluated by analyzing the error distributions for the relative deviation of the experimental and predicted values for *D* (Figure 2 for THF‐*d*_8_ and Figures S22 and S23 for C_6_D_6_ and Tol‐*d*_8_). The relatively small *2σ* errors in the range of 11%–22% for THF‐*d*_8_ compared to previously published models (older publications usually only state 1*σ*) could be explained by the higher flexibility of the fit functions. Nevertheless, this simplified analysis (not incorporating η and *T*) allows for useful insights into the quality of correlation with the respective volume parameters for *D*: For volumes derived from summation approaches (*V*_cov_ and *V*_∑vdW_) the largest 2σ errors are obtained; in the case of THF‐*d*_8_ up to 22%. Using *V*_vdW_ and *V*_C_ gives smaller 2σ errors (11% and 12%). This shows that summation approaches are less suitable measures of the molecular volume in this context. In contrast, *V*_vdW_ and *V*_C_ are derived from the actual shape of the molecule, while *V*_C_ even accounts for the solvent accessible surface of the analyte.

**FIGURE 2:**
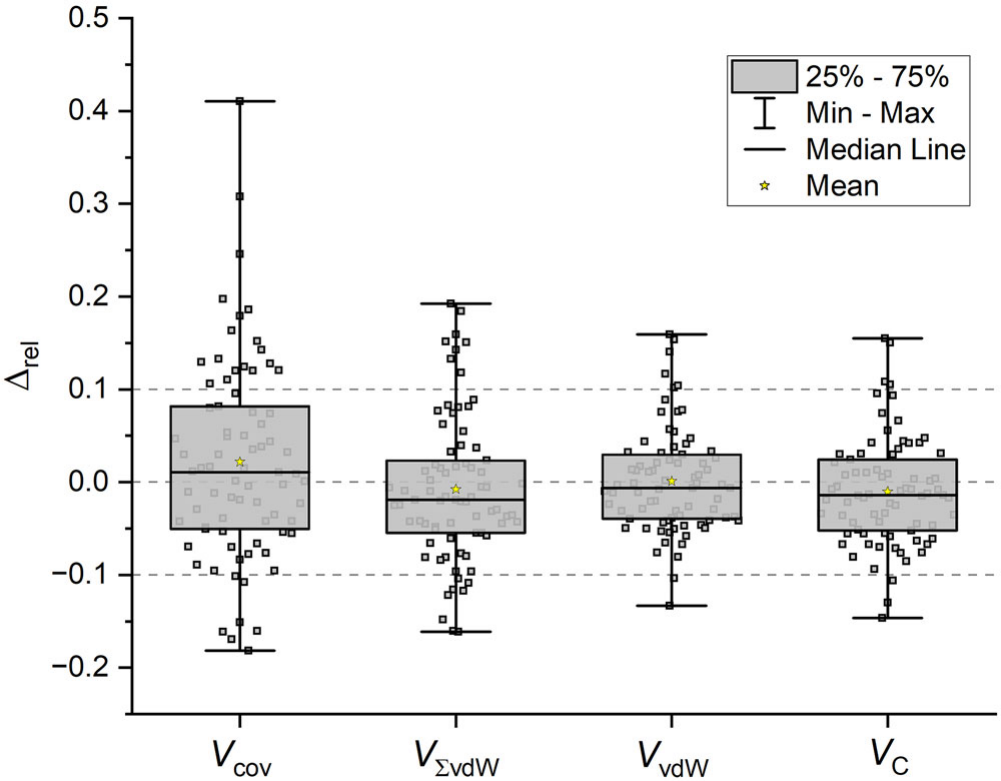
Relative deviation between experimental and predicted *D* in THF‐*d*_8_ from power‐laws derived with different measures of volume. Error bars indicate the *2σ* range.

Next, we tested the *Gierer‐Wirtz* model, which takes microfriction into account by extending the *Stokes*‐*Einstein* equation with the microfriction factor *f*_t_ (Equations 3 and 4; further details in the Supporting Information):

(3) $$\begin{array}{c} D=\frac{k_{B}T}{6\pi \eta rf_{t}}\; \end{array}$$

(4) $$\begin{array}{c} f_{t}={\left(\frac{3a_{\mathrm{GW}}}{2}+\frac{1}{1+a_{\mathrm{GW}}}\right)}^{-1}\; \end{array}$$

with a_GW_ = *r*_L_/*r*, where *r*_L_ is the radius of the solvent and *r* the radius of the analyte molecule. Based on the insights into the suitability of different volume descriptors, we tested *r*_g_, *V*_vdW_, and *V*_C_ for deriving *r* for the *Gierer‐Wirtz* model under the assumption of a perfect sphere for the derivation of *r* from *V*_vdW_ and *V*_C_ (Figures S25–S30). *r*_g_ was assumed to be equal to *r*.

We found that the *Gierer*‐*Wirtz* model systematically overestimates *D* (Figure 3; Figures S26, S28, and S30 and Tables S14–S16), even more than the *Stokes*‐*Einstein* equation. For the *Gierer‐Wirtz* model based on *r*_g_ this phenomenon is even larger, with a *2σ* error of over 87% in all solvents (Table S14). Therefore, using length parameters for the description of molecular geometries in the analysis of diffusion data does not seem to be appropriate.

**FIGURE 3:**
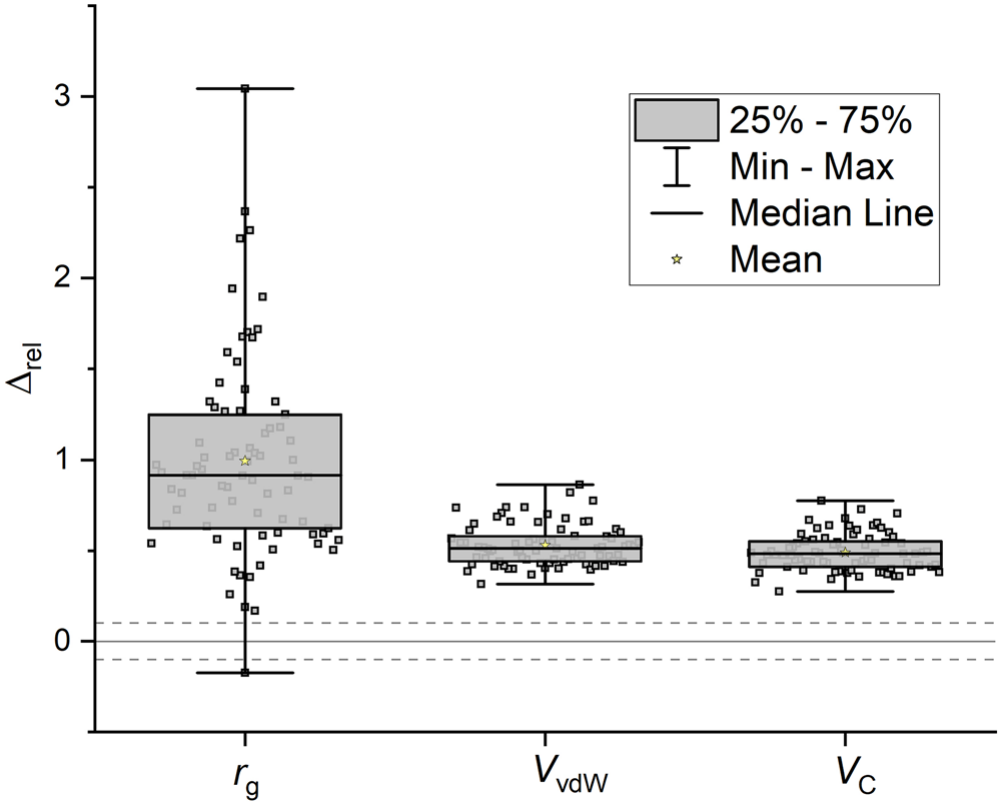
Relative deviations of the values for *D* obtained from the *Gierer*‐*Wirtz* model based on *r*_g_, V_vdW_, and V_C_ and from experimental results in THF‐*d*_8_.

The 2*σ* error of the *V*_C_‐based *Gierer‐Wirtz* model is smaller compared to the *r*_g_‐based model (21% THF‐*d*_8_; 19% C_6_D_6_; 26% Tol‐*d*_8_), but still double compared to the simple power laws used above. The same is observed for the *V*_vdW_‐based *Gierer‐Wirtz* model. If microfriction had a significant influence on diffusion, similar or even smaller 2*σ* errors than the ones obtained from the power law fits would be expected. As *V*_C_ and *V*_vdW_ give similar results, but *V*_C_ is computationally more expensive to obtain, we base our further considerations only on *V*_vdW_.

To check whether the *Gierer‐Wirtz* model is too rigid for the description of microfriction in a broad range of analytes, we analyzed our data set with the semiempirical model by *Chen* and *Chen*, which uses experimentally fitted microfriction factors (Equation 5 and chapter 2.1.2 of the Supporting Information) and should show an improvement when compared to the empirical power‐laws [27].

(5) $$\begin{array}{c} f_{t}={\left(1+0.695\;{\left(\frac{\sqrt[{3}]{r_{\mathrm{BvdW}}^{3}+N_{S}r_{\mathrm{BvdW};L}^{3}}}{\sqrt[{3}]{n_{L}}\cdot r_{\mathrm{BvdW};L}}\right)}^{2.234}\right)}^{-1}\; \end{array}$$

with *N_S_* the number of solvent molecules coordinated to the analyte, *r*_BvdW;L_ the van der Waals radius of the solvent and *n*_L_ the solvent molecular association number.

We found that the model of *Chen* and *Chen* does improve the description of diffusion significantly for the data set (2*σ*: THF‐*d*_8_ 11%; C_6_D_6_ 17%; Tol‐*d*_8_ 13%, see Table S17 and Figure S31) when compared to *Gierer* and *Wirtz*. Nevertheless, there is a remarkable overestimation of *D* with ∼20% for THF‐*d*_8_ and toluene‐*d*_8_.

To verify whether the general approach of a microfriction correction based on *a*_GW_ is feasible, we plotted *a*_GW_^−1^ versus *f*_t_, where *f_t_* is derived from the experimental *D* using Equation 3 (Figure 4).

**FIGURE 4:**
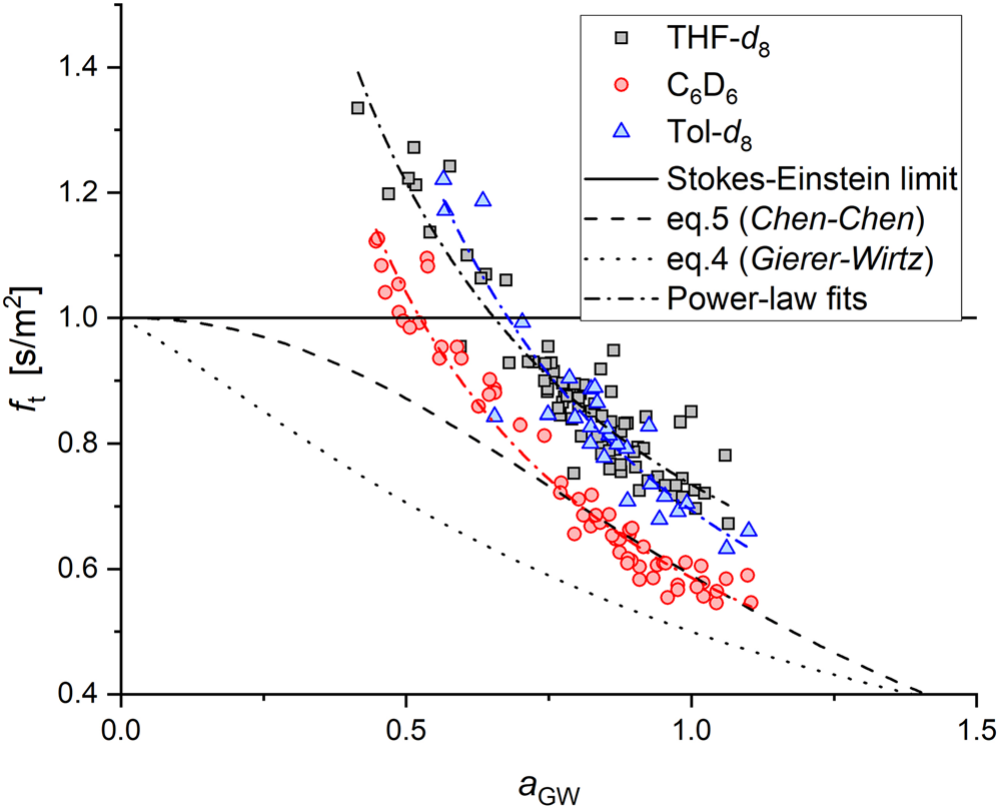
*Spernol‐Wirtz* plot. Data points are *Stalke* normalized, lines represent different models of diffusion [28].

The type of plot in Figure 4 was first utilized by *Spernol* and *Wirtz* and was the basis for the later *Gierer*‐*Wirtz* theory [5, 28]. *Chen* and *Wei* used a related version of it for their extended *Chen* and *Chen* model. If *f*_t_ = 1, the experimental values essentially follow the *Stokes‐Einstein* equation, referred to as the *Stokes*‐*Einstein* limit by *Chen* and *Wei* [27]. This limit should be approached asymptotically if the molecule size becomes large compared to the solvent (small *a*_GW_ values). If the *Gierer* and *Wirtz* microfriction theory, and models based on it, were correct, *f*_t_ values >1 would not be possible. Plotting the dataset, however, shows *f*_t_ values >1 (Figure 4). To test whether the observed dependency of *a*_GW_ on *f*_t_ is an effect of microfriction or just a coincidence, we derived power law fits for the dataset in the three solvents (Figure 4 and Table S18). The relative error distributions (Figure 5 and Table S18) reveal that the 2*σ* errors of the power‐laws for THF‐*d*_8_ (11%) and C_6_D_6_ (9%) are the same as the 2*σ* errors of the purely empirical models using *V*_vdW_. In contrast, Tol‐*d*_8_ shows an increased 2σ error of 46% (12% for the empirical model with *V*_vdW_). Following this, it can be concluded that the microfriction theory based on *a*_GW_ in general seems less likely to be correct.

**FIGURE 5:**
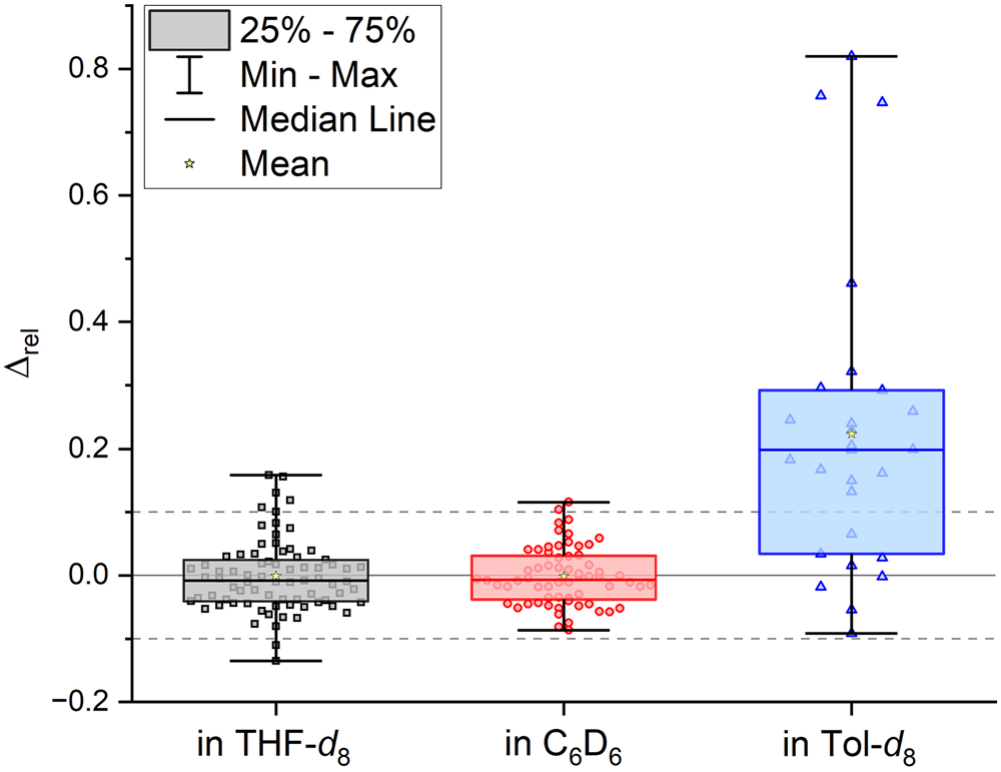
Box plot of error distributions of the *Spernol‐Wirtz* plot power‐law fits.

To test whether there is any microfriction effect present at all in our dataset, we plotted the deviations of our purely empirical *V*_vdW_ power‐law against *a*_GW_ (Figure 6). No trend is visible, indicating that it is unlikely that effects of microfriction according to *Gierer* and *Wirtz* influence diffusion of the small molecules in our dataset.

**FIGURE 6:**
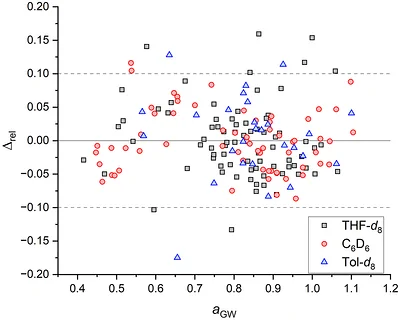
Relative deviation of the empirical power‐law of diffusion versus *a*_GW_.

The increased accuracy achieved by *Chen* and *Chen*, and *Chen* and *Wei* might be explained by utilization of a more realistic model of molecular volume (*Bondi's* group contribution method) [7, 27, 29].

Additionally, these models are mostly based on a limited variety of organic molecules, with O being the only hetero atom. The high degrees of freedom in their parameters *n_s_* and *N_s_* make the model function highly flexible and thus make the model effectively a purely empirical model for their given dataset [27].

### 2.3 Molar Mass Correlations

A popular approach to diffusion which has been widely used for analyzing diffusion data of various compounds and the generation of new diffusion models is the idea of correlating the diffusion constant with the molar mass of a molecule. This is based on Equation 6, which shows that the diffusion constants of macromolecules can be predicted using a power‐relation and their molar mass *M*:

(6) $$\begin{array}{c} D=cM^{-b}\; \end{array}$$

with *b* the Flory parameter and *c* a constant. One of the most popular models for small molecule diffusion was developed by *Stalke* and coworkers. Based on their normalization method, they developed empirical diffusion models purely relying on *M* and in some cases, a density correction using their so‐called *van‐der‐Waals* density *MD*_w_ (see Supporting Information for more details). This model can be seen as the current gold standard in the analysis of DOSY spectra [9, 13, 15, 30].

To test the more general applicability of this model, we plotted the relative deviation of our dataset in THF‐*d*_8_ for their basic diffusion model (ECC merge) and their density corrected model (MD_w_) against the molecular density ρ_M_ (Figure 7; for definition see Supporting Information, Equation 5).

**FIGURE 7:**
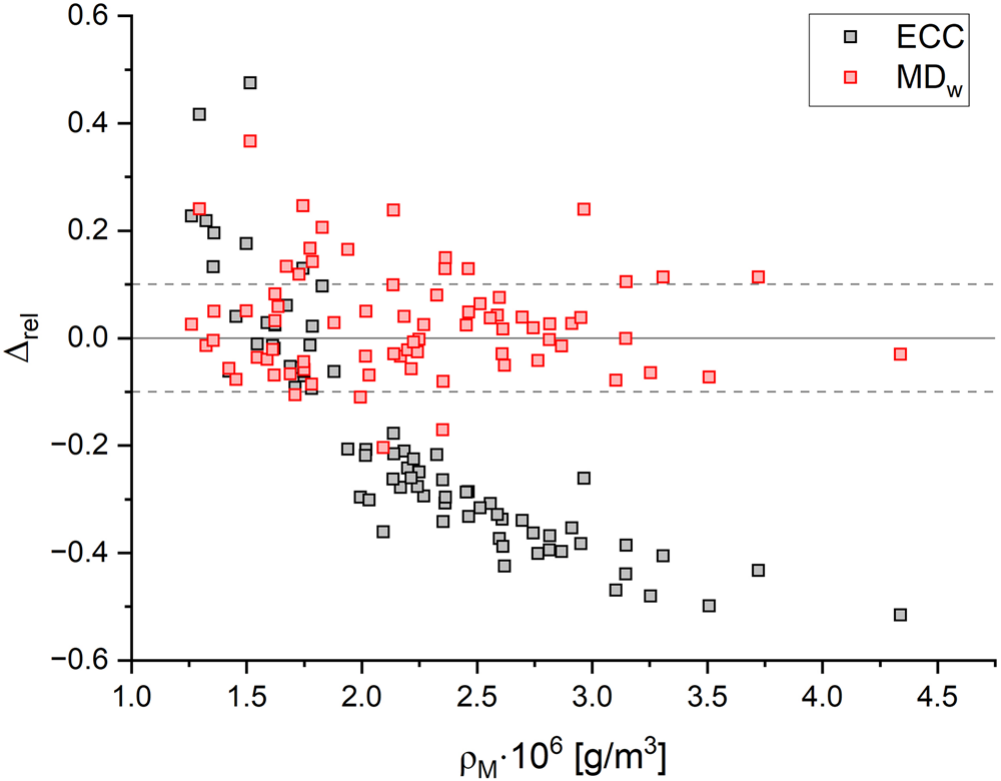
Relative error of *Stalke's* merged power‐law (ECC) and density corrected power‐law (MD_w_) plotted against the molecular density.

For *Stalke's* merged model of diffusion the same trend is visible as discussed in their prior work. The uncorrected model (ECC) has a high degree of preciseness but a systematic error when molecules are studied with densities deviating from the molecules in the calibration set. Interestingly though, through the density correction, the MD_w_ model loses its precision while gaining accuracy (${\bar{\Delta }}_{\mathrm{rel}}$= 7%; 2σ = 24.3%). This could be explained by the work of *Zaccaria* et al. in which the authors state that diffusion is not directly related to molar mass but rather to the volume of a molecule [31]. This hypothesis can be underpinned by the high orders of accuracy of our empirical power laws.

Somewhat at the cross between a *Gierer*‐*Wirtz* and a molar mass correlation is the *Evans‐Morris* model of diffusion. Based on a set of experimental diffusion constants and the assumption of a spherical analyte, they generated a highly effective model of diffusion, using a parameter called “effective density”. This model is able to generate accurate estimation of the molar mass of an analyte from its diffusion constant, as long as the effective density of the analyte does not deviate strongly from the one used in the model, excluding atoms heavier than chlorine [3, 8]. Nevertheless, the model recently showed some success in the analysis of molecules with higher densities such as coordination compounds [32]. We tested this model against our dataset. Plotting the relative errors against the molecular densities (Figure 8) reveals that similarly to the uncorrected *Stalke* model, this model also suffers from an error dependency on the molecular density ρ_M_, as noted by *Morris* and *Evans* themselves. Therefore, the utilization of this model for coordination compounds seems questionable. The same error dependency is observed when using the recently developed model by *Hiller*, *Grabe*, and *Schonert* (see Figure S33) [33].

**FIGURE 8:**
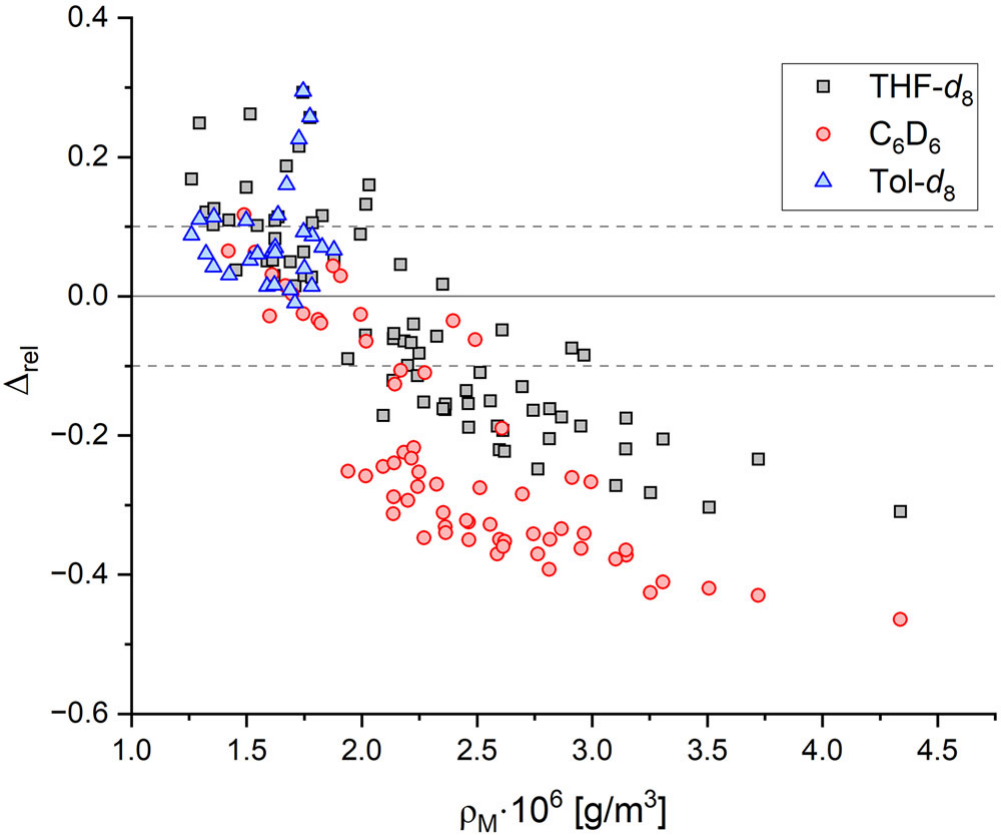
Relative errors of the *Evans‐Morris* model plotted against the molecular density.

As all the studied molar mass correlations (without density correction) of diffusion show a drift in their deviations, it becomes more likely that a function linking diffusion to molar mass must also incorporate a molecular density parameter. This might suggest that diffusion can be better described by a molecular volume parameter.

### 2.4 Stokes‐Einstein Revisited—Development of a New Volume‐Based Model

Up until today, the *Stokes*‐*Einstein* model remains the most utilized model of diffusion in DOSY and beyond. The biggest drawback for applying it is the question of how to correctly utilize the hydrodynamic radius *r*_H_, originally defined as the size a sphere would have that diffuses equal to the analyte. Based on our above observations, we propose a new interpretation of *r*_H_ which links the radius of the hypothetical sphere to a characteristic geometric parameter *x*. This is done through an empirical model, thus generating a semiempirical *Stokes*‐*Einstein* equation:

(7) $$\begin{array}{c} D=\frac{k_{B}T}{6\pi \eta }\cdot \frac{1}{r_{H}\left(x\right)}\; \end{array}$$

As outlined above, a descriptor of molecular volume seems to be the most suitable candidate for a characteristic property for describing *r*_H_ [31]. Even though *V*_C_ should be the physically more meaningful descriptor for the molecular volume in the context of diffusion, *V*_vdW_ gave very similar results in the fully empirical power law described above and thus seems to be equally well suited in this context. We therefore tested both *V*_vdW_ and *V*_C_ as volume descriptors for the semiempirical *Stokes‐Einstein* model. Accordingly, we plotted *r*_H_ versus *V*_vdW_^−1^ and *r*_H_ versus *V*_C_^−1^ (Figures S34 and S35). The dependency is best described by a power law (Equation 8; Tables 1 and S19):

(8) $$\begin{array}{c} r_{H}=a\cdot {\left(V_{\mathrm{vdW}}\right)}^{b}\; \end{array}$$

**TABLE 1:** Fitted parameter for the semi‐empirical *Stokes‐Einstein* model based on *V*_vdW_, with Å^(1‐3b)^ being the unit of a, and b being the fit parameter b.

	a [Å^(1‐3b)^]	b	R^2^	2σ
THF‐*d*_8_	0.163±0.007	0.570±0.008	0.98	11%
C_6_D_6_	0.112±0.005	0.599±0.008	0.99	9%
Tol‐*d*_8_	0.1±0.1	0.65±0.02	0.96	12%

The fit based on *V*_C_ gave slightly worse 2σ errors (10%–15%) compared to the one using *V*_vdW_. Additionally, *V*_vdW_ is computationally easier accessible, and we therefore used *V*_vdW_ for our further considerations.

The *2σ* errors of our new model (Figure 9 and Table 1) are the same as those generated by our empirical power law model (11% in THF‐*d*_8_, 9% in Tol‐*d*_8_ and 12% C_6_D_6_). The reasons for this are trivial, as both model types are essentially the same, where in the empirical version the parameters *T*, *η* and the constant *k*_B_/(6π) are incorporated into the parameters of the power law itself.

**FIGURE 9:**
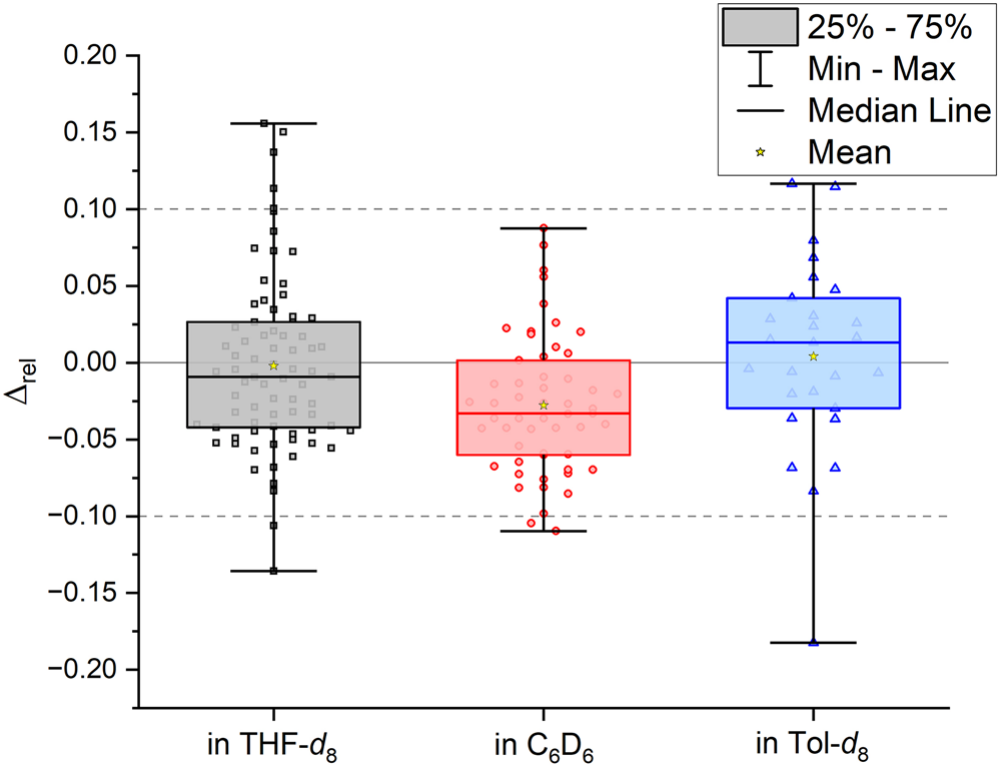
Box plot of error distributions of our semiempirical *Stokes‐Einstein* model (Equations 7 and 8).

Even though a 2σ uncertainty of 12% is sufficient for most questions arising in coordination chemistry or from the analysis of mixtures of organic compounds, outperforming the density corrected *Stalke* model by a factor of two, there is still room for improvement.

Several possible shortcomings can be identified in our model, which could be addressed to improve the error:

1. Differences in shape of the studied molecules.
2. Density differences of the analytes.
3. Structural flexibility of analyte and solvent.
4. Charge or partial charges on the surface of the analyte and the solvent.

The first point can be understood as how close the molecules relate to a perfect sphere, that is, does the sphericity ψ of the molecule correlate with the relative deviation of our model. The sphericity can be defined as [34]:

(9) $$\begin{array}{c} \Psi =\frac{\pi^{\frac{1}{3}}\cdot {\left(6V_{\mathrm{vdw}}\right)}^{\frac{2}{3}}}{A_{\mathrm{vdW}}}\; \end{array}$$

with *A*_vdW_ the *van der Waals* surface area.

Plotting the relative errors against the sphericities (Figure 10) clearly shows that Ψ does not correlate with the relative error and therefore this potential issue can be excluded.

**FIGURE 10:**
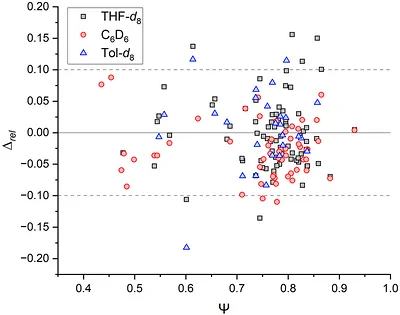
Relative deviations of our semiempirical *Stokes*‐*Einstein* model plotted against the corresponding sphericities Ψ (Equation 9) of the studied data set.

To evaluate the second potential shortcoming, that is, the density component, we tested our model in the same manner as the molar mass correlation models for a dependency on the molecular density (Figure 11). No dependency on the density was found for our semiempirical *Stokes*‐*Einstein* model.

**FIGURE 11:**
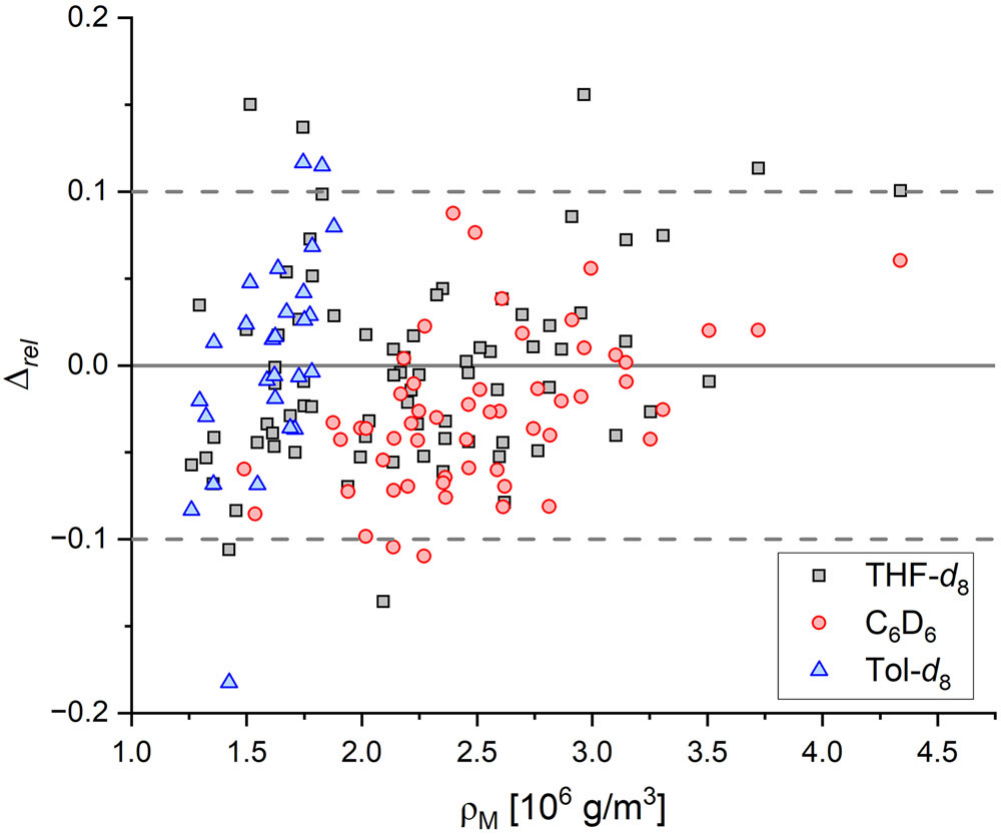
Relative errors of our semiempirical *Stokes‐Einstein* model plotted against the molecular density.

The third potential issue raises the question whether the collisions between solvent and analyte during diffusion can be seen as elastic or inelastic collisions. In a more flexible molecule, more of the energy of the collision will be converted into deformation of the molecule, that is, the inelastic part of the collision. To investigate this fully, more experimental data and theoretical studies are needed and therefore this point will be revisited in the future.

The last potential source for errors is on the question of how (partial) charges influence diffusion. This is very reminiscent of the stick or slip boundary conditions of other diffusion models. To give conclusive answers on this, we are planning to study diffusion behaviour of charged species in the future.

Even though our model still has room for additional refinements, it has one major advantage over the molar mass‐based model of diffusion (besides its higher accuracy and precision)—its biunique nature. In case a DOSY spectrum of an entirely unknown species should be analyzed, our model is still able to determine the analyte's *V*_vdW_ to a high degree of accuracy. The MD_W_ model, in contrast, is a function with two variables and thus no conclusive answer can be given of the mass of the species when only *D* is known.

### 2.5 The Semiempirical Stokes‐Einstein Model in Practise

Next, we tested the performance of the new model on a range of uranyl complexes (Figure 12), since only ∼20% of the available dataset consisted of coordination compounds. The speciation of uranyl complexes is of interest both for fundamental studies in coordination chemistry as well as for the speciation of environmental samples [35]. The uranyl ion (UO_2_^2+^) is linear and therefore ligand coordination occurs exclusively in the equatorial plane, leading to nonspherical molecular shapes. Additionally, through the presence of a uranium atom, the compounds possess relatively high molecular densities (∼2.7∙10^6^ g/m^3^). All these factors limit in theory the applicability of the other diffusion models for uranyl complexes.

**FIGURE 12:**
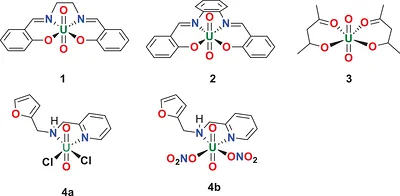
Uranyl complexes analyzed with our semiempirical *Stokes*‐*Einstein* model. Coordinated THF molecules are not shown.

Complexes **1**, **2,** and **3** were reproduced from the previous reports [36, 37, 38], while complexes **4a** and **4b** were newly synthesized.

The diffusion constants were determined experimentally in THF‐*d*_8_ (Supporting Information chapter 7). Since THF is known to coordinate to uranium, we aimed to determine the number of coordinated THF molecules. *V*_vdW_ for **1**, **2,** and **3** were determined based on reported crystal structures, which were used as input for force field optimizations to include additional coordinated THF molecules. This method seemed sufficiently accurate, as the error of the DOSY measurement itself is likely larger than the difference in molecular volume between force field optimization and DFT. For example, for **2** the *V*_vdW_ is 303 Å^3^ without coordinated THF as derived from the crystal structure. Using force field optimization, a *V*_vdW_ of 308 Å^3^ is obtained—a deviation of 1.6%.

For comparison, we applied both *Stalke's* density corrected model MD_w_ and our model on the three known compounds (**1**, **2**, **3**). The results in dependency of the number of coordinated THF molecules can be found in the Supporting Information (Supporting Information chapter 7). The best matching possible structures of *Stalke's* MD_w_ have one (for **1**) or two (for **2** and **3**) coordinated THF, with errors of −0.2% (**1**), −8.2% (**2**), and −5.9% (**3**). Our model suggests the same numbers of coordinated THF molecules with similar errors of ‐7.6% (**1**), 1.9% (**2**), and ‐5.1% (**3**).

The PyNHFu ligand in **4a** and **4b** has a higher flexibility with regards to the coordination mode compared to the ligands in **1**, **2**, and **3**. The potential coordination isomerism influences the *V*_vdW_ of the studied structural models, as can be seen from the *V*_vdW_ estimations for **4a** without coordinated THF (Figure 13). The deviations of the calculated *V*_vdW_ lie within 5%, which is within the 1σ range of our model. This might indicate that upon further validation with other systems, our model could be applied for studying the conformation of a given compound. As at this point the necessary reliability is not yet reached, we utilized only the isomers for which minima were obtained in DFT‐based geometry optimizations for the analysis of **4a** and **4b**.

**FIGURE 13:**
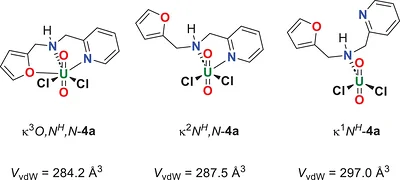
Coordination isomers of 4a with corresponding *V*_vdW_. Structures were derived by deleting coordinated THF from the DFT‐optimized structures.

The parent structure of complex **4b** without THF coordinated to uranium was obtained from x‐ray diffraction analysis of suitable crystals (see Supporting Information chapter 6) and used as a starting point for a DFT‐based geometry optimization of THF‐coordinated complexes of **4b** (see Supporting Information chapter 18) [39]. From the potential structures, κ^2^ *N^H^,N*‐**4b** with one THF coordinated was found to be the best match with a deviation of ‐1.5% (Figure S17). The MD_w_ model gave the same structural match with a slightly lower deviation (0.1%), but no information on the bonding isomerism. Interestingly, in the crystal structure of **4b**, THF is not coordinated to the uranium directly, but linked via a hydrogen bond to the secondary amine of the ligand — an isomer with a slightly larger volume and thus a slightly larger mismatch with the DOSY data and our model of analysis (2.7%, 389.3 Å^3^). This might indicate different structures of the compound in solution and solid state.

The structural model for **4a** was also determined using DFT methods, taking different coordination modes of the ligands into consideration (Figure 13 and Supporting Information chapter 18). In the analysis using our model, a κ^1^ *N^H^* **‐4a** structure with one THF bound via a hydrogen‐bond to the proton of the secondary amine and one THF bound to the uranium centre shows the highest degree of accuracy (2.3% deviation, Figure S15). MD_w_ yielded similarly a compound with two coordinated THF and similar accuracy (−1.2%).

For the uranyl complexes studied here, our model and the MD_W_ model performed very similarly. For the full dataset, however, our model shows a higher precision, making it more reliable for compounds outside the calibration range of the MD_W_ model.

Comparing the volumes of the best matching models of chloro complex **4a** [UO_2_κ^1^‐(PyNHFu)Cl_2_(thf)_2_] (439 Å^3^), and nitrate complex **4b** [UO_2_κ^2^‐(PyNHFu)(NO_3_)_2_(thf)] (394 Å^3^), a volume difference of ∼12% is found between the two. This size difference is well within the 2σ limit of our model, which indicates that the accuracy of the model is high enough to allow the study of the influence of small NMR‐silent groups, like in this case Cl^−^ versus NO_3_^−^.

## 3 Conclusion

Using a data set of 95 organic, organometallic and coordination compounds in three different solvents for which *Stalke*‐normalized diffusion constants are available, we elucidated the shortcomings of various models for analyzing diffusion constants of molecules. We reconfirmed that purely relying on the *Stokes‐Einstein* model of diffusion is not a valid approach when it comes to describing diffusion of smaller molecules and complexes.

Furthermore, evidence was found that microfriction theories based on the *Gierer* and *Wirtz* model are also not applicable for the studied data set. We found additional evidence that the hypothesis of *Zaccaria* is correct: the molecular weight does not describe diffusion directly, but through the introduction of density corrections, volume models are generated which then describe diffusion for small molecules. We therefore developed a semiempirical *Stokes*‐*Einstein* model of diffusion based on the *van‐der‐Waals* volume *V*_vdW_ of a molecule. This model overall outperforms the currently widely used models by a factor of two when it comes to accuracy, reducing the 2σ error for the studied data set to ∼12%. The sphericity and mass or density of the analyte molecule, which cause errors for most other models, could be ruled out as potential sources for error. Additionally, our model describes diffusion in a biunique nature, allowing for the determination of analytically useful results for *V*_vdW_ from *D* without the need for a structural model.

Finally, the application of the new model in the study of five uranyl complexes was demonstrated, with errors as low as ‐8% to 2%, enabling the determination of the number of coordinated THF molecules and small ionic ligands in solution.

Future work will investigate the limits of this model with regards to the size of molecules, as well as investigate the influence of structural flexibility and charges on the diffusion and its potential inclusion in the model. Furthermore, we plan to extend the model to other commonly used solvents. Additionally, we encourage others to test the model for questions on solution speciation of different types of molecules, such as metal complexes, main group and organic compounds.

## Conflicts of Interest

The authors declare no conflicts of interest.

## Supporting information



The authors have cited additional references within the Supporting Information [40, 41, 42, 43, 44, 45, 46, 47, 48, 49, 50, 51, 52, 53, 54, 55, 56, 57, 58, 59, 60, 61, 62, 63, 64, 65, 66, 67, 68, 69, 70, 71, 72, 73, 74, 75, 76, 77, 78, 79, 80, 81, 82, 83, 84, 85, 86, 87, 88, 89, 90, 91, 92, 93, 94, 95, 96, 97, 98, 99, 100].
[Correction updated on 5th August 2026 The supporting information was updated in this version.]
**Supporting File 1**: chem71471‐sup‐0001‐SuppMat.pdf

**Supporting File 2**: chem71471‐sup‐0002‐SuppMat.xlsx

## Data Availability

The data that supports the findings of this study are available in the Supporting Information of this article. Crystallographic data for 4b has been deposited at the CCDC under deposition number 2521038 and can be obtained free of charge from https://www.ccdc.cam.ac.uk/structures/.
