# Supplementary material for: Accurate Molecular Size Determination by Diffusion Ordered NMR Spectroscopy Based on an Improved Diffusion Model
Source: Chemistry. 2026 Jul 24;32(40):e71471. doi: 10.1002/chem.71471 (PMC13716011; doi:10.1002/chem.71471)
Supplement: Supplementary file 1 [file CHEM-32-e71471-s001.pdf]

Supporting Information for

Accurate Molecular Size Determination by Diffusion Ordered NMR Spectroscopy Based on an Improved Diffusion Model

Christian Urbank[a,b] and Lisa Vondung*[a]

[a]Department of Chemistry, University of Hamburg, Martin-Luther-King-Platz 6, 20146 Hamburg, Germany
[b]Laboratory for Zero-Carbon Energy, Institute of Integrated Research, Institute of Science Tokyo, N1-32, 2-12-1, Ookayama, Meguro-ku, Tokyo 152-8550, Japan

Table of Contents

1 General Information 3
2 Discussion of diffusion models 3
2.1 The Stokes-Einstein equation and modifications thereof 3
2.1.1 Microfriction: Gierer and Wirtz modification 3
2.1.2 Microfriction: Chen and Chen modification 4
2.1.3 The Evans model 4
2.2 Power laws: Stalke's external calibration 4
3 Synthesis of the Uranyl complexes 6
4 NMR Spectra 7
5 ATR-IR 9
6 Crystallographic Data 10
7 DOSY-Spectra 12
8 Benchmarking dataset 18
9 Holtz Viscosity Adjustment 23
10 Calculation of Errors 23
11 Power Laws inspired by the Gierer-Wirtz Model – Testing for the Right Volume Descriptor 24
12 Gierer-Wirtz Models 28
13 Chen and Chen Model 31
14 Spernol-Plot Power-Law fits 32
15 Hiller, Grabe and Schonert Model 33
16 Semi-empirical Stokes-Einstein Fits 34
17 Step-by-Step Guide 35
18 Computational Methods 36
18.1 Coordinates of Optimized Structures 36
18.1.1 Organic Molecules 36
18.1.2 Uranyl Coordination Compounds 52
19 References 68

1 General Information

The literature data was analysed using *Origin 2023b*. For the calculation of the radius of gyration *OVITO Basic 3.10.2* has been used.[1] MoloVol 1.1.1 has been used for calculating V_{vdW} and V_C.[2]

2 Discussion of diffusion models

The following sections provide a short overview of the historic development of different approaches to describe diffusion of molecules, their application and shortcomings. There is a plethora of different models and theories of diffusion, and therefore we will only discuss the most prominent ones. A more detailed discussion can be found in the review of Robert Evans.[3]

2.1 The Stokes-Einstein equation and modifications thereof

The *Stokes-Einstein* equation 1 is the most prominent diffusion model. It relies on the radius of a particle as the characteristic molecular parameter:

$$D = \frac{k_B T}{6\pi\eta a} = \frac{k_B T}{6\pi\eta r_H} \tag{1}$$

where k_B is the *Boltzmann* constant, T the temperature, η the dynamic viscosity and a the *Stokes* radius. Here, *Einstein* combined the notion of a statistical process of *Brownian* motion with the hydrodynamics of the *Stokes* law. While equation 1 can successfully be applied to larger systems, the hydrodynamics of the *Stokes* law have two major drawbacks: First, the *Stokes* law in the *Stokes-Einstein* equation itself is defined for a perfect sphere and therefore real predictive power of equation 1 only applies within these restrictions. Deriving the *Stokes* law for non-spherical particles would require a more complicated solution of the *Navier-Stokes* equation and thus it was mostly attempted to circumvent this problem for more practically useful methods.[3,4]

The most straightforward strategy in this regard was the redefinition of a as the hydrodynamic radius r_H, which is the radius a sphere would have that diffuses in the same manner as the analyte. Through this, equation 1 is commonly applied to obtain information on the general size of a molecule under the assumption that the radius of the hypothetical sphere somewhat resembles the size of the molecule. The second drawback of the *Stokes* law hydrodynamics is a possible collapse of the solvent continuum assumption, if the size of the analyte is comparable to the surrounding solvent molecules. Thus, the individual interactions between solvent and analyte molecules might have to be considered in a model.[5]

2.1.1 Microfriction: Gierer and Wirtz modification

The theory of microfriction introduced by *Gierer* and *Wirtz* tries to tackle the problem of interaction between solvent and analyte via the introduction of a microfriction correction factor f_t. As a basis of their model, they defined a more characteristic way to describe the analyte. For this, they utilize the radius r derived from the molar volume V_m of the analyte. V_m is converted to a radius r_m under the assumption of a perfect sphere. To correct for packing effects from the solid state from which V_m is derived, atomic packing factors are used to convert r_m to r. In their publication, *Gierer* and *Wirtz* already questioned the validity of this approach, but as no viable method for determining the real volume of a molecule was known at the time they proceeded.[6,7]

Their model of diffusion can be seen as a small molecule adapted version of the *Stokes-Einstein* equation. The microfriction correction factor f_t is incorporated as follows:

$$D = \frac{k_B T}{6\pi\eta r f_t} \tag{2}$$

where the *Gierer-Wirtz* model defines f_t as:

$$f_t = \left(\frac{3a_{GW}}{2} + \frac{1}{1 + a_{GW}}\right)^{-1} \tag{3}$$

with $a_{GW} = r_L/r$, r_L being the radius of the solvent. This model has been shown to be more accurate for small molecules in general compared to the original *Stokes-Einstein* model in equation 1. Nonetheless, it still deviates from the actual experimental data significantly.

2.1.2 Microfriction: Chen and Chen modification

A semiempirical model that seemingly compensates the errors of the *Gierer-Wirtz* model was introduced by *Chen* and *Chen*.[8] They determined f_t experimentally by rearranging equation 3 and fitting it against a reciprocal a_{GW} denominated as a_{CC}. They circumvented the size characterization problem of *Gierer* and *Wirtz* by generating a *van der Waals* radius of the analyte r_{BvdW} by using *Bondi*'s group contribution method.[9] In this method volume increments for the participating atoms V_{BvdW} are added together and then converted to r_{BvdW} by assuming a perfect sphere. Following this, equation 4 is obtained for f_t:

$$f_t = \left(1 + 0.695\left(\frac{\sqrt[3]{r_{\mathrm{BvdW}}^3 + N_S r_{\mathrm{BvdW;L}}^3}}{\sqrt[3]{n_{\mathrm{L}}} \cdot r_{\mathrm{BvdW;L}}}\right)^{2.234}\right)^{-1} \tag{4}$$

with N_S the number of solvent molecules coordinated to the analyte, $r_{BvdW;L}$ the *van der Waals* radius of the solvent and n_L the solvent molecular association number. This model was shown to be very effective in a wide range of applications over a range of solvents and temperatures.[10–12]

2.1.3 The Evans model

A second semi-empirical model based on *Gierer-Wirtz* theory was introduced by *Evans*.[13,14] In this model, the molecular weight *M* was correlated with *D* through the optimization of an empirical parameter called the effective density ρ_{eff}. For their optimization a set of different organic molecules was used as calibration points. This method generates accurate results (±15 %; 2σ error ±30 %) if the real molecular density ρ_M of the analyte does not deviate much from the ρ_M of the calibration dataset.
As the definitions of density parameters used in some of the diffusion models are somewhat arbitrary, we define the molecular density ρ_M as

$$\rho_{\mathrm{M}} = \frac{M}{V_{\mathrm{vdW}} \cdot N_{\mathrm{A}}} \tag{5}$$

with *M* the molar mass, V_{vdW} the *van-der-Waals* volume and N_A Avogadro's constant. This definition is more aligned with the classical definition of density and therefore should make this variable more comparable.
As the *Evans* model of diffusion utilizes the ratio of *M* and ρ_{eff}, it effectively evolves into a semiempirical model which uses the volume of the molecule.

2.2 Power laws: Stalke's external calibration

More recently, *Stalke*'s molecular weight correlation has become the most widely used diffusion model for small molecules.[15–18] Originally inspired by the internal standards of *Willard* and co-workers, the *Stalke* model is based on the power law in equation 6.[19–21] In this model, molecules are grouped in three classes according to their shape: compact sphere, dissipated sphere/ellipsoids, and expanded disc. The model only becomes viable if the *Stalke* normalization (see equation 2 in the main manuscript) is applied before, but if done, the external calibration curve of the *Stalke* model can generate highly accurate results (±10 %; 2σ error ±20 %).

$$D = \mathrm{A} \cdot M^{-\mathrm{a}} \tag{6}$$

with A and a the calibration parameters.

Additionally, a model for molecules of unknown geometry was generated with higher uncertainties, but still giving reasonable results. The early model was highly dependent on a parameter called the molar *van der Waals* density MD_W.[15] This parameter is defined as the ratio of M and the sum of the *van der Waals* volumes $V_{\Sigma vdW}$ of the atoms in the analyte. Thus, the original model could not be used for molecules with an MD_w deviating strongly from $5.2 \cdot 10^{29}$ g mol^{-1} m^{-3}. This drawback led to the development of a correction factor for the merged model that uses MD_W. With the merged model, an accurate data set of coordination compounds was generated (2σ error ±20 %).[17,18] *Note*: The revised parameters from the 2016 publication were used for the ECC_{merge} model both for the ECC_{merge} and MD_W models applied in this study, as these are the ones included in the software provided by Stalke and co-workers.[22]

The combination of a density and a mass parameter makes *Stalke*'s model at its core a volume-based model of diffusion. The elegance of this approach lies in the choice of appropriate calibration points. The external calibration curve can only give a rough estimation over a broad range, but if new calibration points are chosen which are closer to the target analyte, a deviation of only ±5 % is possible.

3 Synthesis of the Uranyl complexes

Caution: Compounds containing uranium in the natural isotope composition are radioactive and must therefore be handled following appropriate safety measures to prevent inhalation, ingestion and extended exposure.

All reactions that required inert atmosphere were conducted under argon in an MBraun Glovebox or using Schlenk techniques. THF and *n*-Pentane were dried over Na/K alloy, degassed and subsequently stored over 3 Å molecular sieves. $[UO_2Cl_2(thf)_2]$ was synthesized from $UO_2(NO_3)_2{\cdot}6\ H_2O$ using concentrated HCl and TMS-Cl according to a reported procedure.[23] UO_3 was synthesised according to Nipruk *et al.*.[24] $[UO_2$(salophen)(thf)], $[UO_2$(salen)(thf)] and $[UO_2(acac)_2(thf)]$ were synthesized following literature procedures.[25–27] *N*-(2-Furanylmethyl)-2-pyridinemethanamine (PyNHFu) was synthesized according to Jana *et al.*.[28] All organic reactants for the synthesis of PyNHFu were purified via distillation.

Synthesis of **$[UO_2(PyNHFu)Cl_2(thf)_2]$ (4a):** 20 mg PyNHFu (0.106 mmol) was dissolved in 5 ml THF. 51.5 mg $[UO_2Cl_2(thf)_2]$ (0.106 mmol) was added in 10 ml THF to the orange solution. The resulting yellow suspension was stirred at room temperature over night, after which a yellow suspension was obtained. The solid was filtered off through celite and THF was removed *in vacuo* from the yellow filtrate. The resulting yellow solid was dissolved in 2 ml THF and crystallised as a yellow solid and dried with the open vial, resulting in 43.6 mg of yellow crystalline material (61.1 %). **^{1}H NMR** (300 MHz, THF-d_8) δ = 10.39 (d, *J*=5.5, 1H, H-1), 8.19 – 8.08 (m, 1H, H-3), 7.80 (d, *J*=7.7, 1H, H-4), 7.73 (t, *J*=6.6, 1H, H-2), 7.58 (d, *J*=1.9, 1H, H-12), 6.43 (t, *J*=2.5, 1H, H-11), 6.38 (d, *J*=3.2, 1H, H-10), 6.32 (s, 1H, H-7), 5.31 (s, 1H, H-6''), 4.71 (d, *J*=2.7, 2H, H-8), 4.01 (s, 1H, H-6'). **^{13}C NMR** (151 MHz, THF-d_8) δ = 161.2 (C-5), 155.1 (C-1), 151.7 (C-9), 143.0 (C-12), 139.9 (C-3), 124.2 (C-4), 123.2 (C-2), 110.3 (C-10), 110.0 (C-11), 52.8 (C-8), 44.7 (C-6), 44.6 (C-6). **IR** (cm^{-1}): 3262(m), 3076(m), 2936(w), 1605(m), 1483(w), 1428(m), 1339(w), 1310(w), 1247(w), 1152(w), 1144(w), 1093(w), 1041(m), 1009(s), 913(s), 859(s), 830(m), 761(m), 748(s), 738(m), 673(m).

Synthesis of **$[UO_2(PyNHFu)(NO_3)_2(thf)]$ (4b):** 53.4 mg of $[UO_2(NO_3)_2{\cdot}6\ H_2O]$ (0.106 mmol) were dissolved in 10 ml MeOH. A solution of 20 mg PyNHFu (0.106 mmol) in 5 ml MeOH was added to the reaction mixture. After stirring overnight, the solvent was removed *in vacuo*. The solid was dissolved in 1.5 ml THF and filtered through celite. From the resulting clear solution, crystalline needles could be generated via vapour diffusion of 4 ml *n*-pentane. After drying the needles in vacuo, 49 mg of yellow crystalline product were obtained (70.6 %). **^{1}H NMR** (600 MHz, THF-d_8) δ = 9.87 (d, *J*=5.5, 1H, H-1), 8.32 (td, *J*=7.6, 1.6, 1H, H-3), 8.01 (d, *J*=7.8, 1H, H-4), 7.95 (t, *J*=6.6, 1H, H-2), 7.66 (d, *J*=1.8, 1H, H-12), 6.81 (s, 1H, H-7), 6.48 (t, *J*=2.5, 1H, H-11), 6.46 (d, *J*=3.2, 1H, H-10), 5.00 – 4.97 (m, 2H, H-6), 4.81 (d, *J*=66.7, 2H, H-8). **^{13}C NMR** (151 MHz, THF-d_8) δ = 161.9 (C-5), 153.0 (C-1), 151.9 (C-9), 144.7 (C-12), 142.3 (C-3), 126.3 (C-4), 125.8 (C-2), 111.9 (C-10), 111.7 (C-11), 54.4 (C-6), 46.6 (C-8). **IR** (cm^{-1}): 3157(w), 2972(w), 2867(w), 1606(m), 1529(s), 1498(s), 1440(m), 1354(m), 1315(w), 1272(s), 1246(s), 1223(m), 1193(m), 1159(m), 1144(s), 1005(m), 1048(m), 1025(s), 1007(s), 966(w), 938(s), 897(s), 874(s), 859(m), 835(m), 802(m), 759(s), 742(s).

4 NMR Spectra

NMR Spectra were recorded on a Bruker Avance III 600 MHz (600MHz) or a Bruker FourierHD 300 MHz.

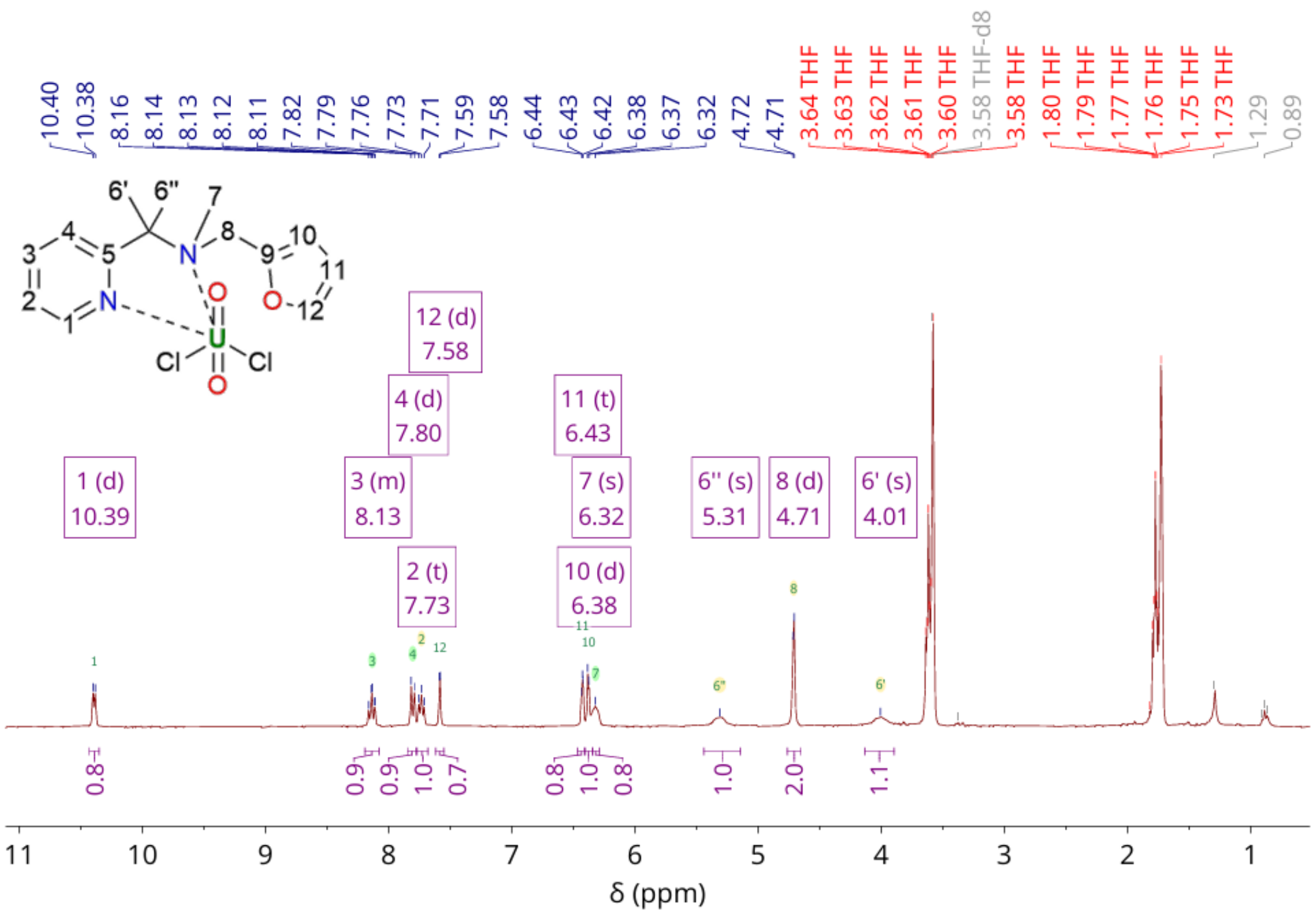

Figure SI-1: ^{1}H-NMR of **4a** in THF-d_8 at room temperature at 300 MHz.

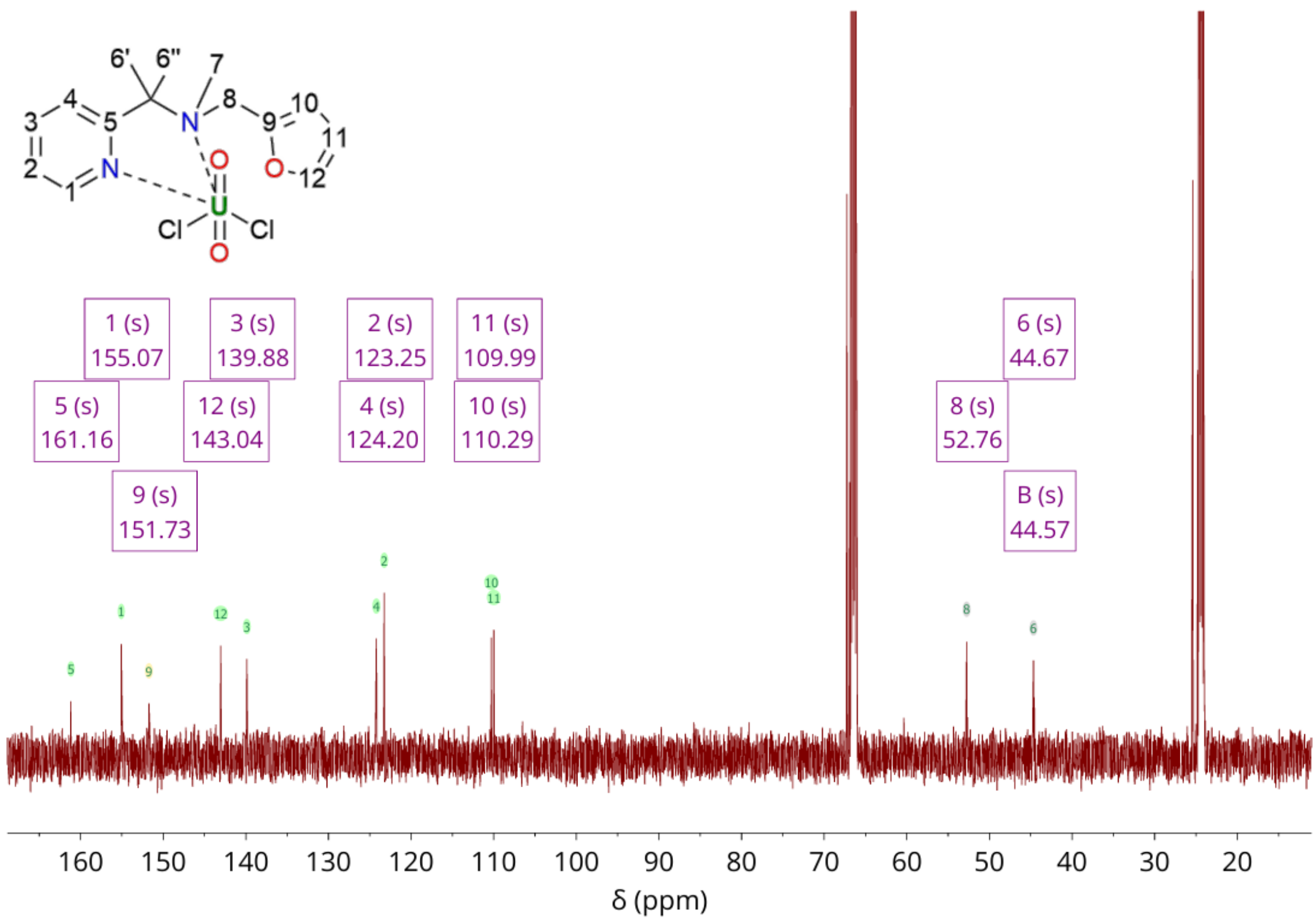

Figure SI-2: ^{13}C{^{1}H}-NMR of **4a** in THF-d_8 at room temperature at 600 MHz.

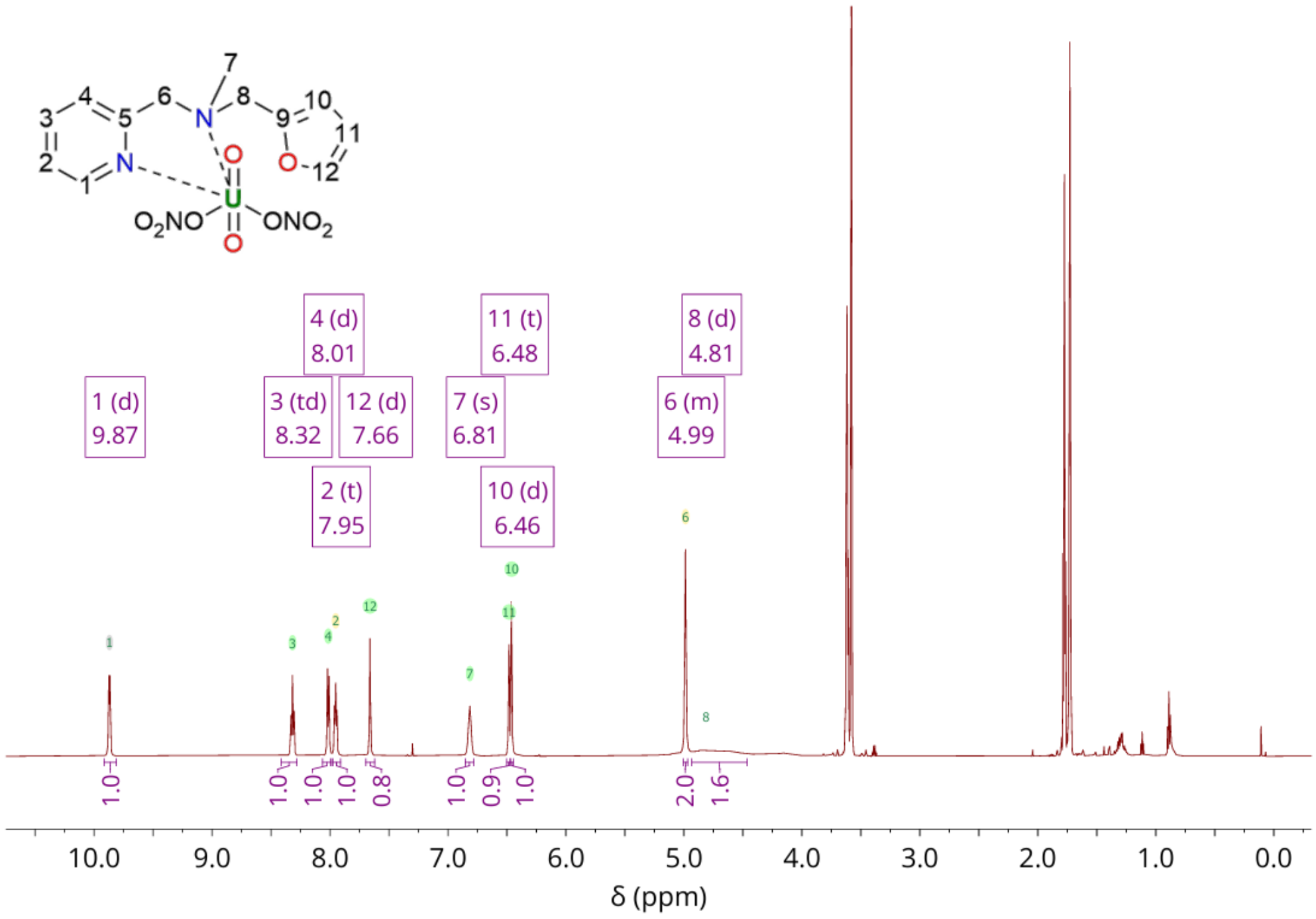

Figure SI-3: ^{1}H-NMR of **4b** in THF-d_8 at room temperature at 600 MHz.

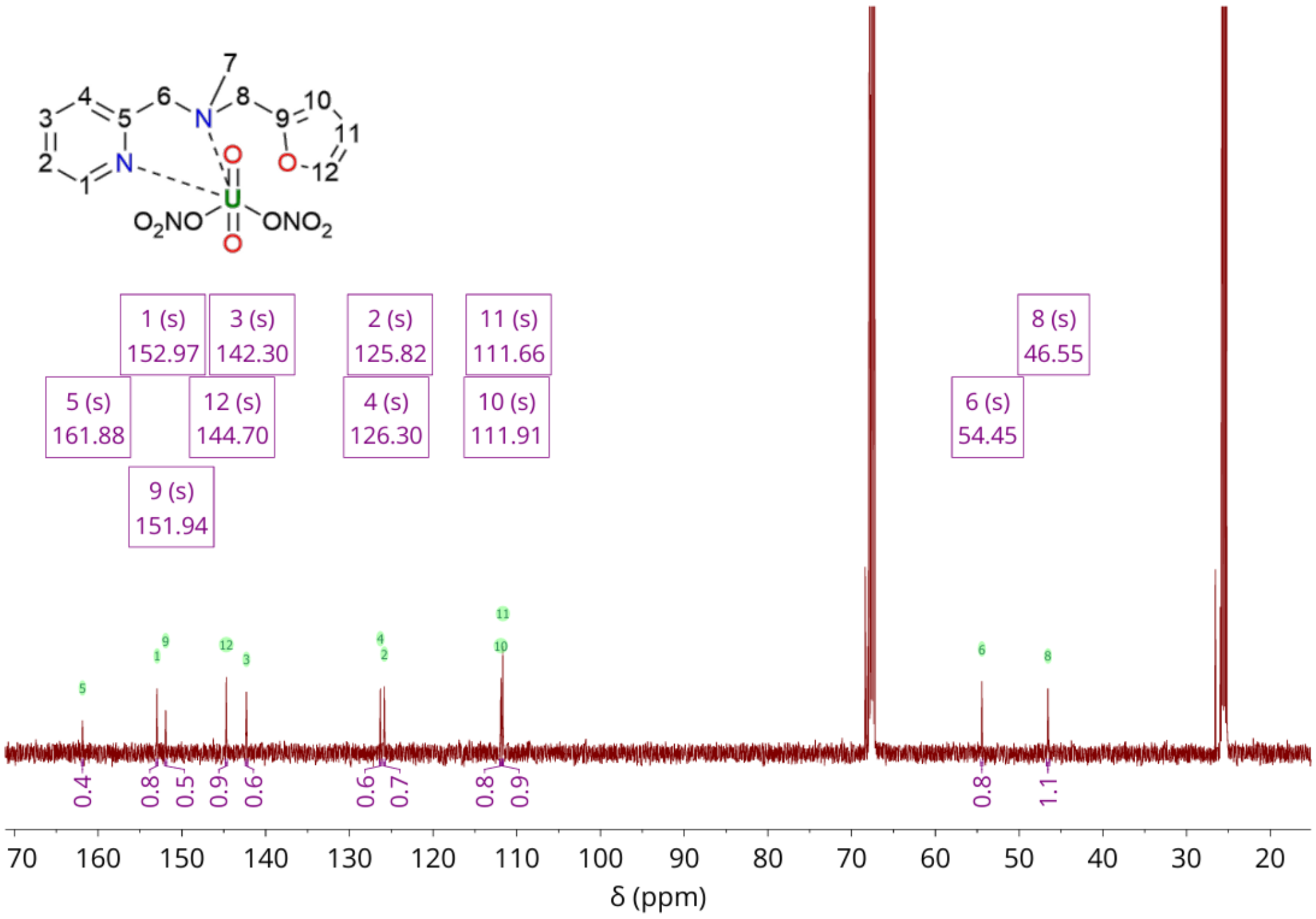

Figure SI-4: ^{13}C{^{1}H}-NMR of **4b** in THF-d_8 at room temperature at 600 MHz.

5 ATR-IR

All FT-IR spectra were recorded on a Cary 630 FT IR from Agilent Technologies in a nitrogen filled glovebox.

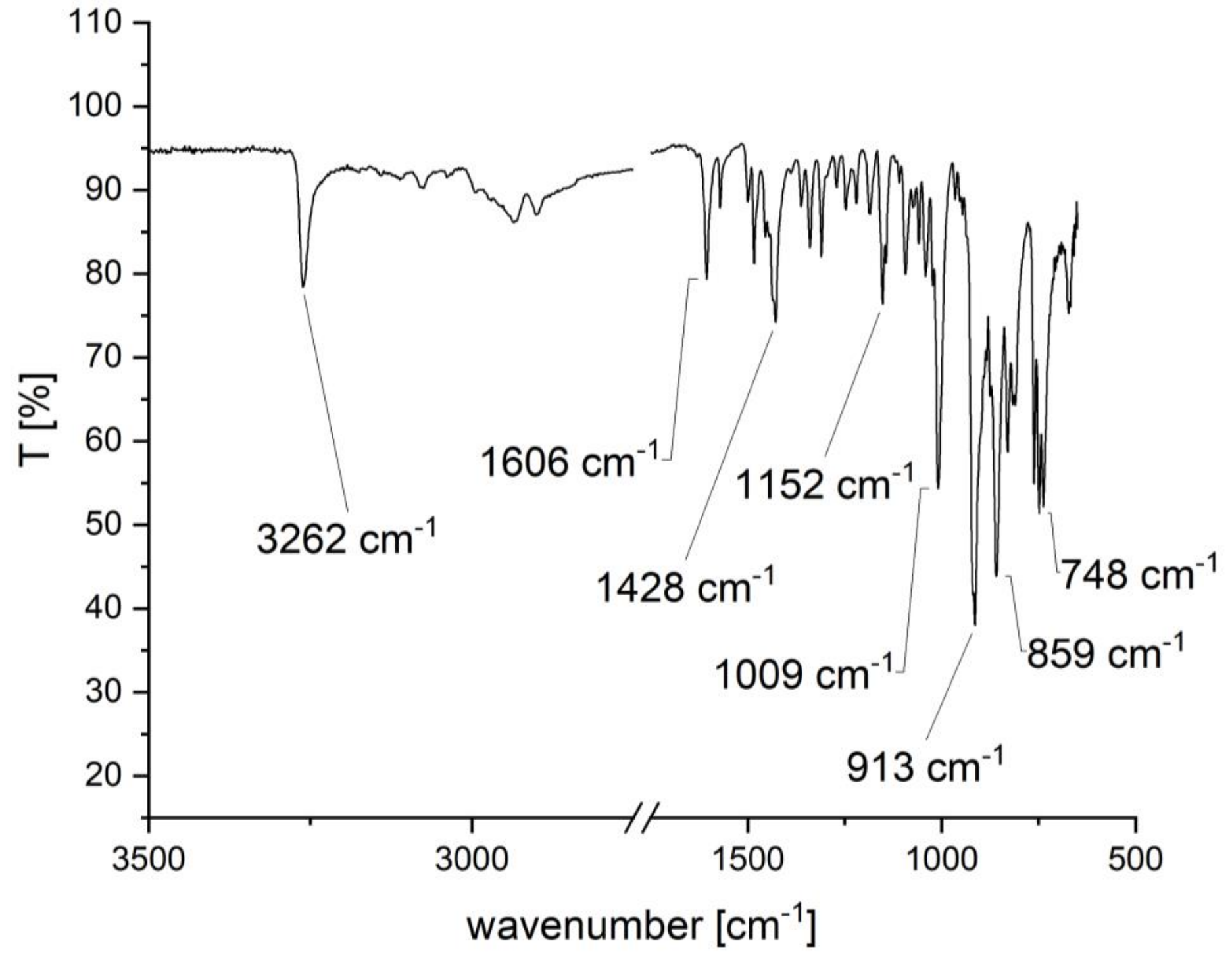

Figure SI-5: FT-IR of **4a**.

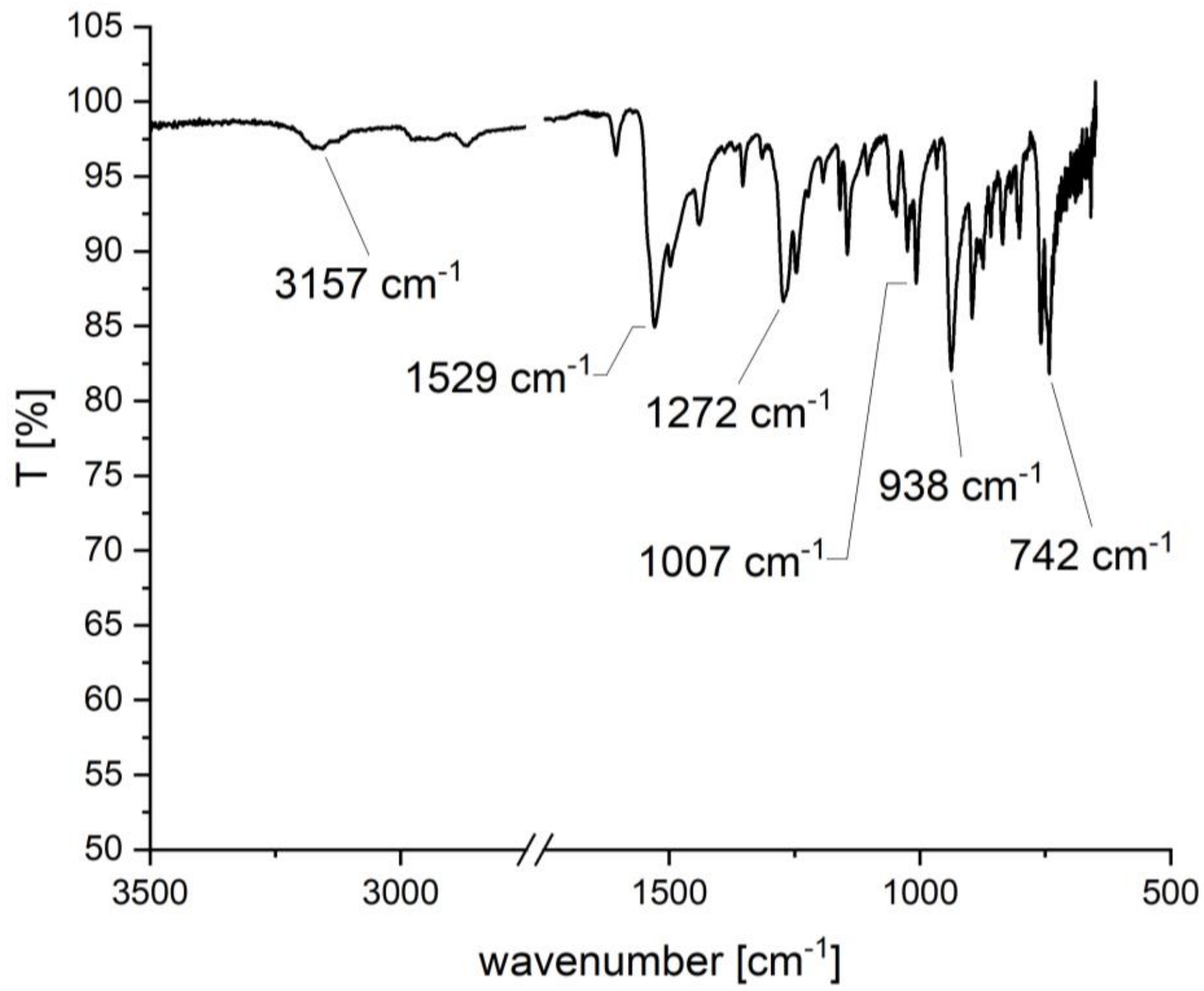

Figure SI-6: FT-IR of **4b**.

6 Crystallographic Data

The single crystal x-ray data was collected at 100 K on a SuperNova four-circle diffractometer in Kappa geometry with a 50 W Cu-Kα microfocus tube, an Atlas CCD detector and a Cryosteam 700 Plus cooler. The structure was solved with SHELXT and refined on F^2 using all reflections with SHELXL.[29,30] Non-hydrogen atoms were refined anisotropically. Hydrogen atoms were placed in calculated positions and assigned to an isotropic displacement parameter of 1.5/1.2 Ueq(C). The crystallographic data can be accessed under accession code CCDC 2521038 from the CCDC database (www.ccdc.cam.ac.uk/structures).

Table SI-1: Crystal data and refinement details for **4b**.

Compound	**4b**
Empirical formula	$C_{15}H_{20}N_4O_{10}U$
Formula weight	654.38
Temperature/K	100.15
Crystal system	monoclinic
Space group	$P2_1/n$
a/Å	13.18570(10)
b/Å	10.23950(10)
c/Å	15.43630(10)
α/°	90
β/°	101.6490(10)
γ/°	90
Volume/Å^3	2041.20(3)
Z	4
ρ_{calc} g/cm^3	2.129
μ/mm^{-1}	22.946
F(000)	1240
Crystal size/mm^3	0.22 × 0.08 × 0.03
Radiation	CuKα (λ = 1.54184)
2Θ range for data collection/°	8.056 to 152.388
Index ranges	$-16 \le h \le 16$, $-12 \le k \le 12$, $-19 \le l \le 19$
Reflections collected	73594
Independent reflections	4247 [R_{int} = 0.0674, R_{sigma} = 0.0166]
Data/restraints/parameters	4247/2/291
Goodness-of-fit on F^2	1.097
Final R indexes [I>=2σ (I)]	R_1 = 0.0213, wR_2 = 0.0585
Final R indexes [all data]	R_1 = 0.0215, wR_2 = 0.0588
Largest diff. peak/hole / e Å^{-3}	2.23/-1.18

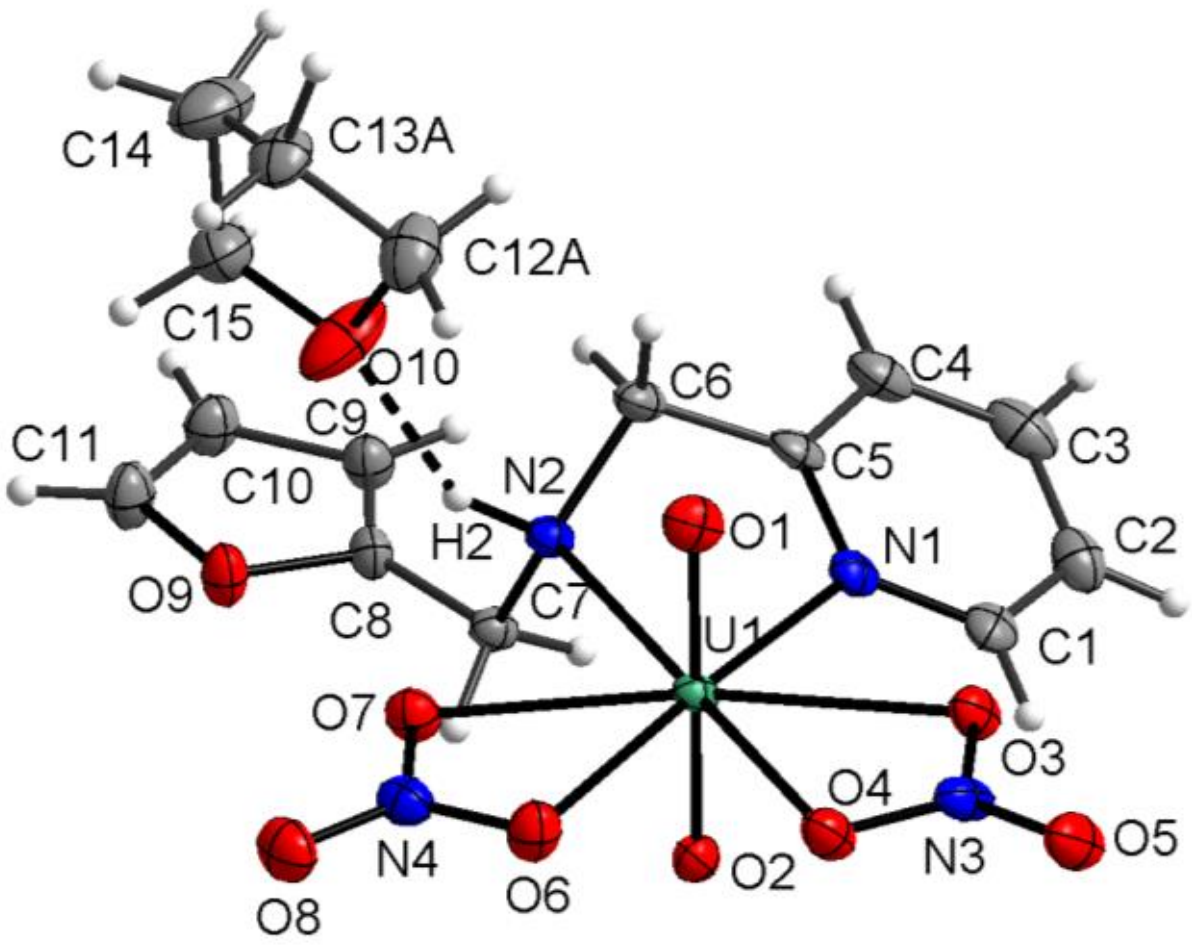

Figure SI-7: Molecular structure of **4b** obtained from single crystal structure x-ray diffraction. Ellipsoids are drawn at 50 % probability. Only one of the two disordered THF molecules is shown for clarity.

7 DOSY-Spectra

All DOSY experiments were recorded using the 600 MHz spectrometer using a double stimulated echo sequence (dstebpg3s). Data processing was conducted using TopSpin 4.3.0 and the Dynamics centre 2.8.4 (Bruker BioSpin GmbH). The solvent signals were used as an internal standard for the Stalke normalization.[15]
The spectra depicted in the following were generated using the Bayesian Method of MestreNova 14.1.0, the result of which was only used for displaying the DOSY spectra and not for the analysis of *D*.

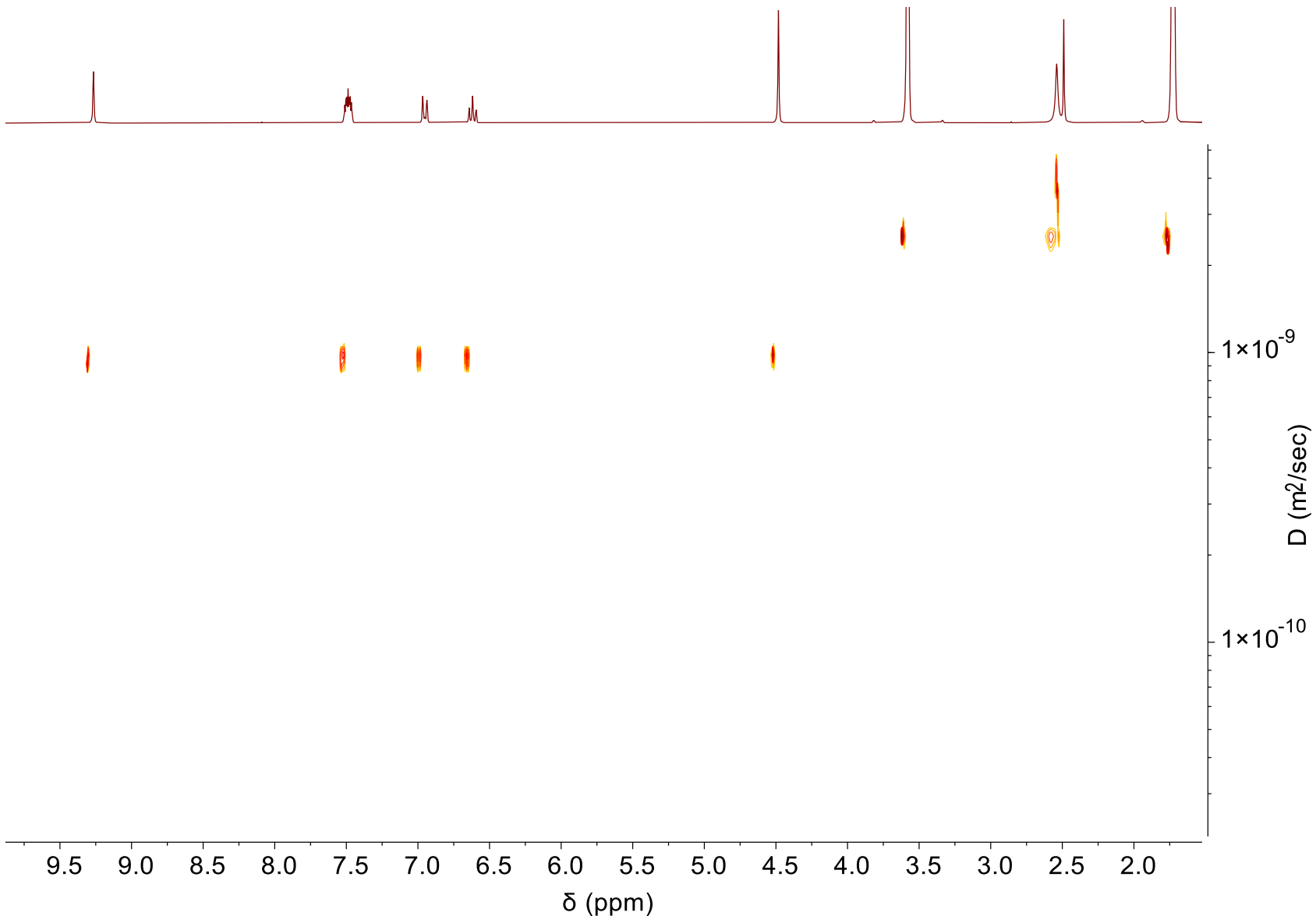

Figure SI-8: DOSY of **1** in THF-d_8 at room temperature.

Table SI-2: Experimental diffusion results for **1**. $V_{vdW,exp}$ is the resulting V_{vdW} according to the semi-empirical *Stokes-Einstein* model derived by us.

D_{ref} [m²/s]	D_x [m²/s]	$D_{x,norm}$ [m²/s]	$V_{vdW,exp}$ [Å³]
$2.573 \cdot 10^{-9}$	$1.006 \cdot 10^{-9}$	$9.095 \cdot 10^{-9}$	397.9

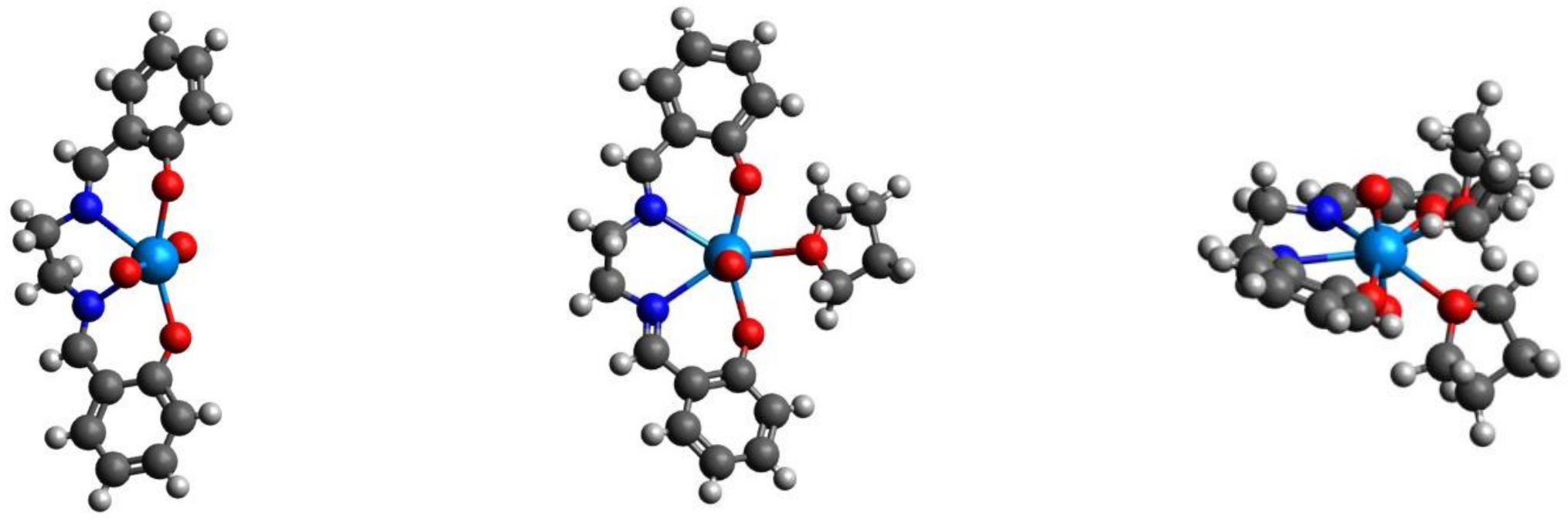

V_{vdW} = 301 Å³<br>Δ = -24.3 %	**V_{vdW} = 368 Å³**<br>**Δ = -7.6 %**	V_{vdW} = 437 Å³<br>Δ = 9.7 %
M_{Stalke} = 652 g/mol<br>Δ_{Stalke} = 21.6%	**M_{Stalke} = 607 g/mol**<br>**Δ_{Stalke} = -0.2 %**	M_{Stalke} = 575 g/mol<br>Δ_{Stalke} = -15.5 %

Figure SI-9: Model structures of **1** with none, one or two coordinated THF molecules, their V_{vdW} and the deviation Δ from $V_{vdW,exp}$. Below, their density corrected *Stalke* molar mass M_{Stalke} and the deviation from M Δ_{Stalke} based on the MD_w model.

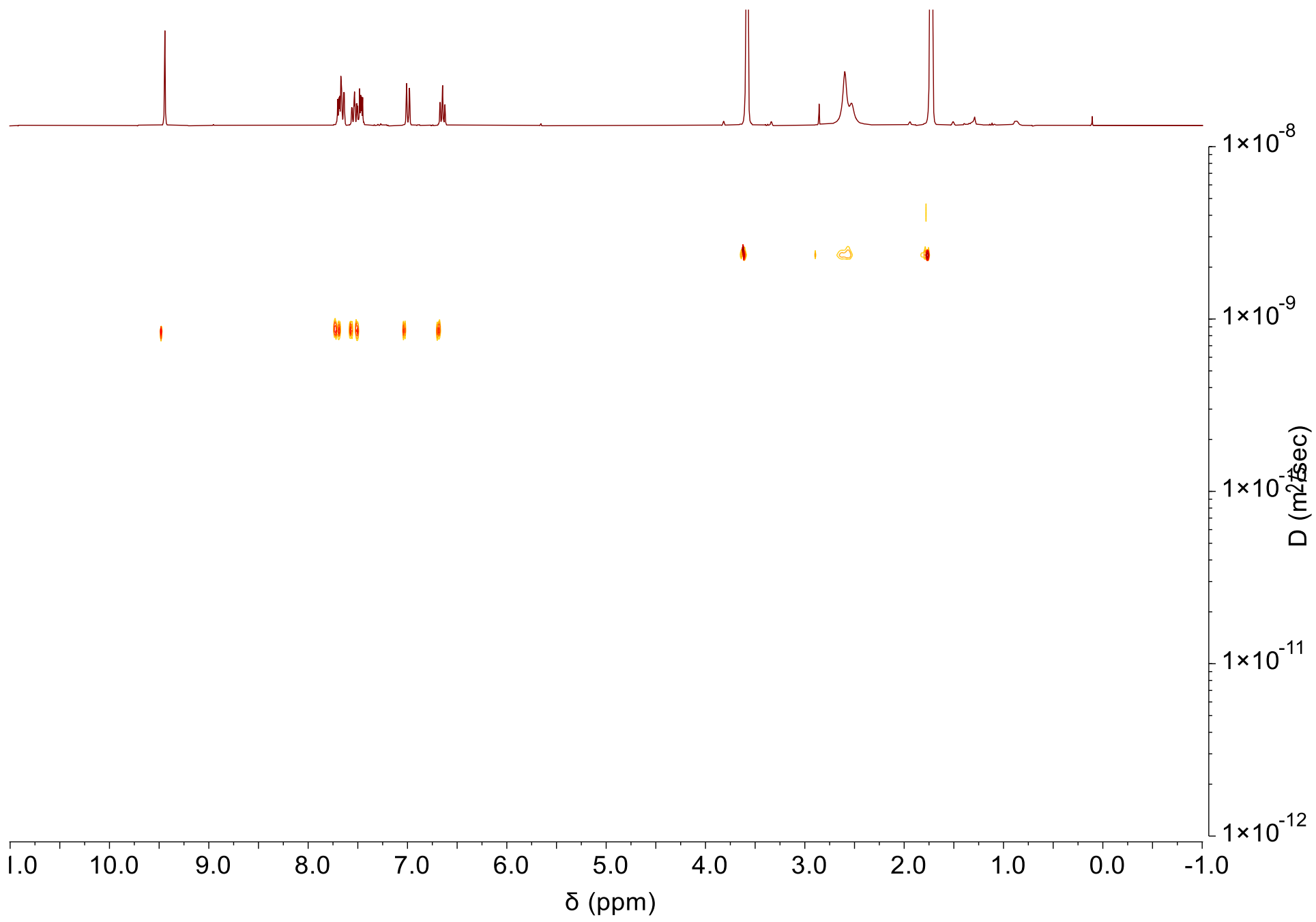

Figure SI-10: DOSY of **2** in THF-d_8 at room temperature.

Table SI-3: Experimental diffusion results for **2**. $V_{vdW,exp}$ is the resulting V_{vdW} according to the semi-empirical Stokes-Einstein model derived by us.

D_{ref} [m²/s]	D_x [m²/s]	$D_{x,norm}$ [m²/s]	$V_{vdW,exp}$ [Å³]
$2.554\cdot10^{-9}$	$9.102\cdot10^{-10}$	$8.288\cdot10^{-10}$	468.3

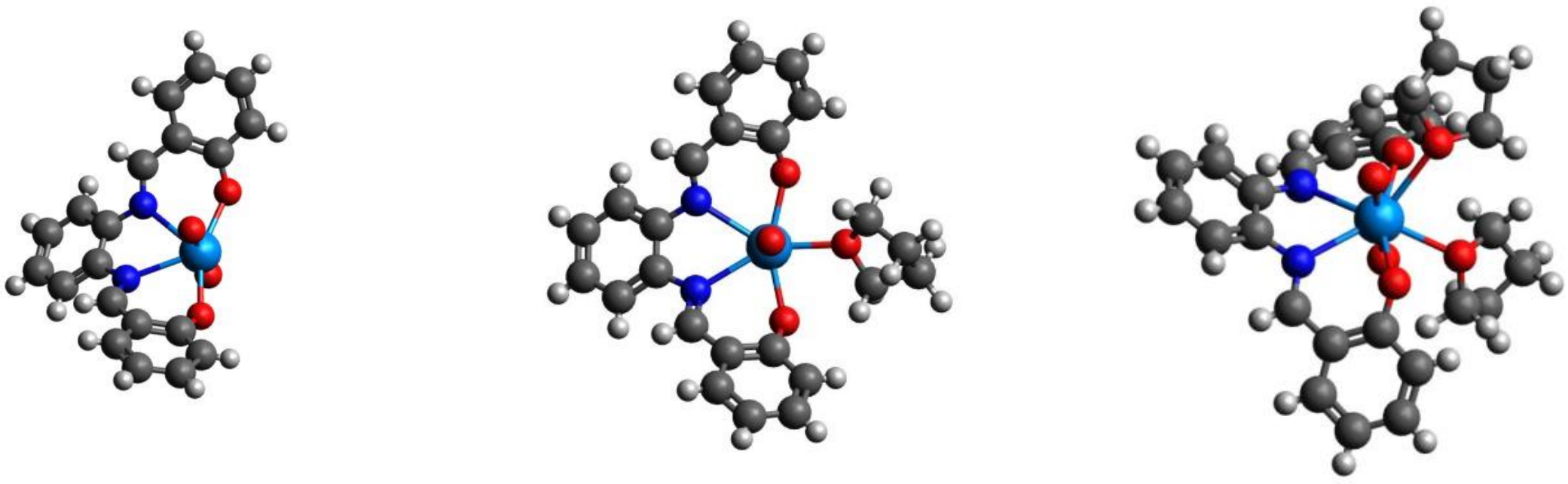

V_{vdW} = 340 Å^3<br>Δ = -27.3 %	V_{vdW} = 408 Å^3<br>Δ = -12.9 %	**V_{vdW} = 477 Å^3<br>Δ = 1.9 %**
M_{Stalke} = 746 g/mol<br>Δ_{Stalke} = 27.6%	M_{Stalke} = 736 g/mol<br>Δ_{Stalke} = 12.1 %	**M_{Stalke} = 669 g/mol<br>Δ_{Stalke} = -8.2 %**

Figure SI-11: Model structures of **2** with none, one or two coordinated THF molecules, their V_{vdW} and the deviation Δ from $V_{vdW,exp}$. Below, their density corrected *Stalke* molar mass M_{Stalke} and the deviation from M Δ_{Stalke} based on the MD$_w$ model.

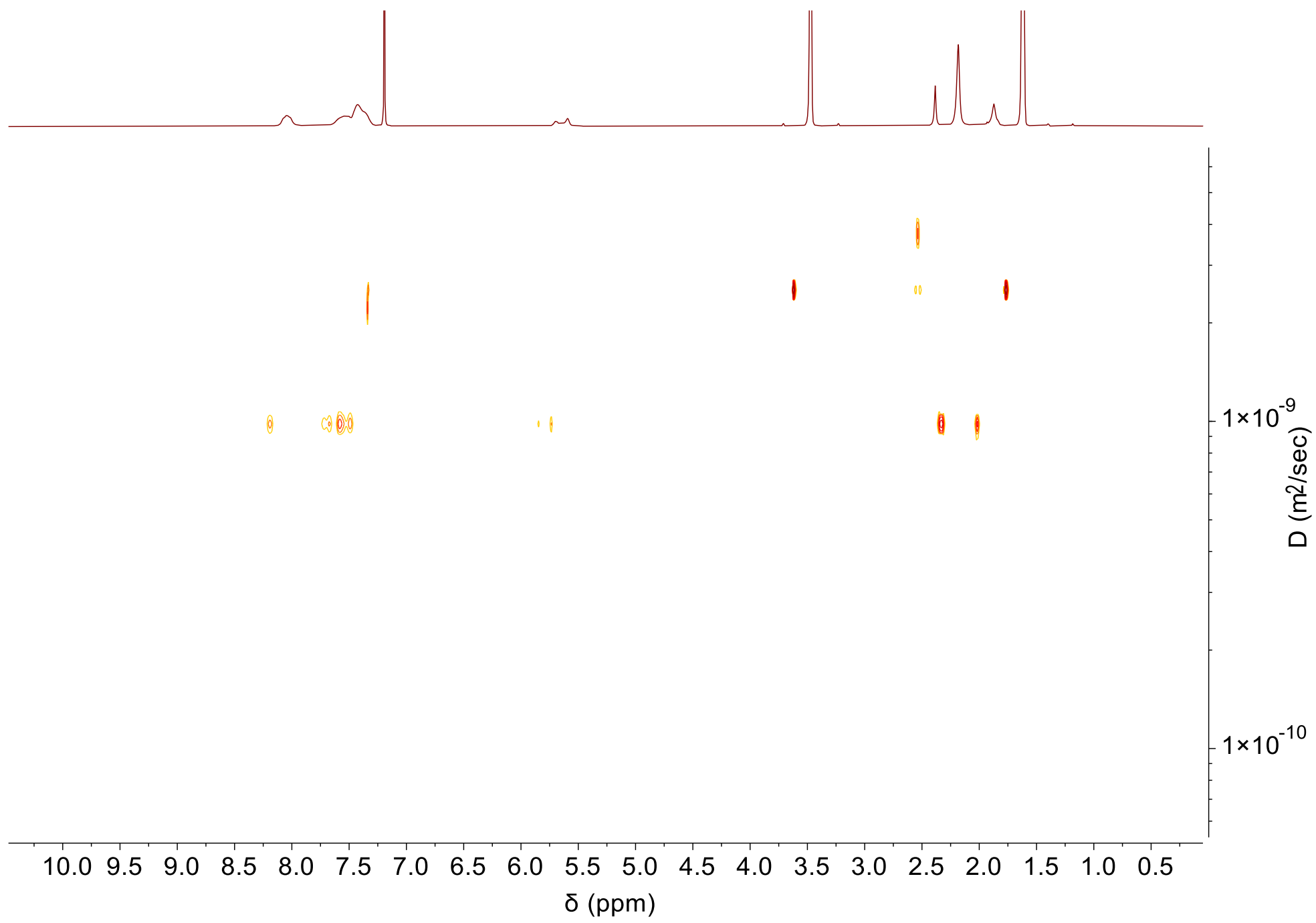

Figure SI-12: DOSY of **3** in THF-d_8 at room temperature.

Table SI-4: Experimental diffusion results for **3**. $V_{vdW,exp}$ is the resulting V_{vdW} according to the model semi-empirical Stokes-Einstein model derived by us.

D_{ref} [m^2/s]	D_x [m^2/s]	$D_{x,norm}$ [m^2/s]	$V_{vdW,exp}$ [Å^3]
$2.606 \cdot 10^{-9}$	$1.028 \cdot 10^{-9}$	$9.176 \cdot 10^{-10}$	391.8

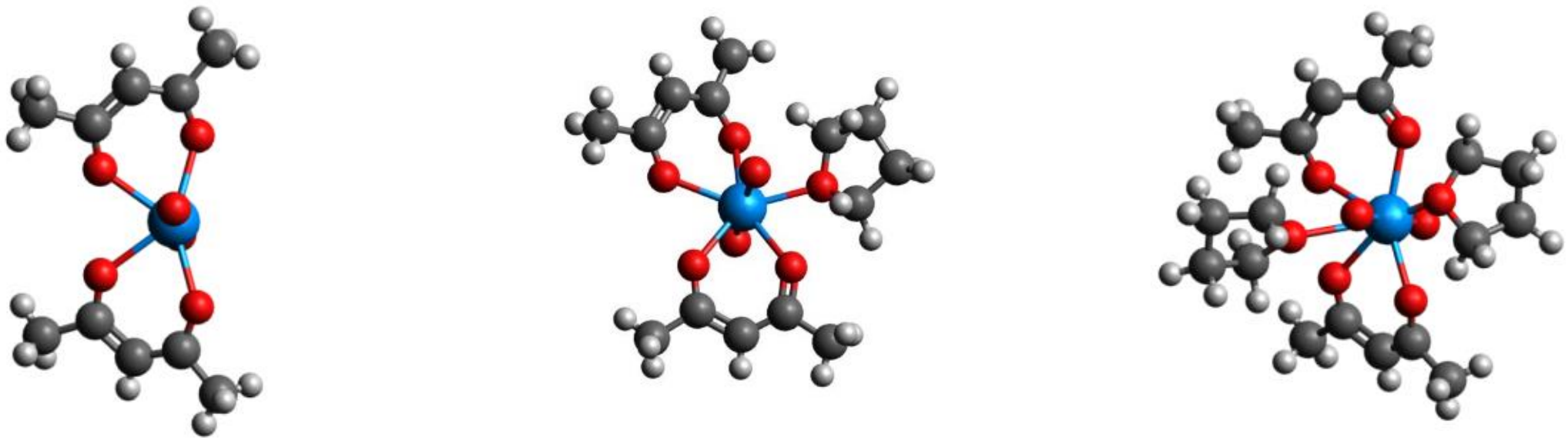

V_{vdW} = 247 Å^3<br>Δ = -37.0 %	V_{vdW} = 313 Å^3<br>Δ = -20.0 %	**V_{vdW} = 372 Å^3**<br>**Δ = -5.1 %**
M_{Stalke} = 676 g/mol<br>Δ_{Stalke} = 43.1%	M_{Stalke} = 619 g/mol<br>Δ_{Stalke} = 13.7 %	**M_{Stalke} = 580 g/mol**<br>**Δ_{Stalke} = -5.9 %**

Figure SI-13: Model structures of **3** with none, one or two coordinated THF molecules, their V_{vdW} and the deviation Δ from $V_{vdW,exp}$. Below, their density corrected *Stalke* molar mass M_{Stalke} and the deviation from M Δ_{Stalke} based on the MD_w model.

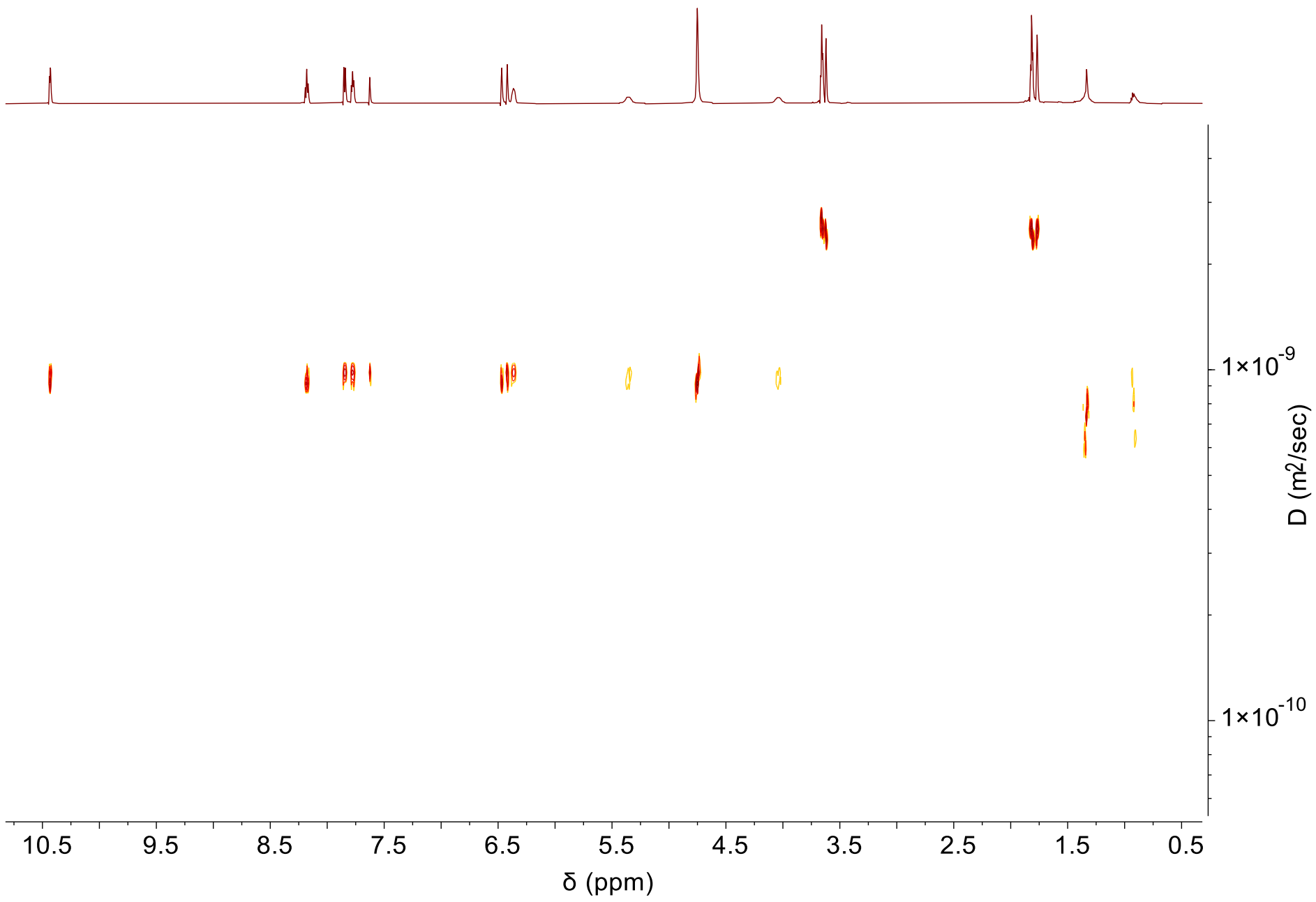

Figure SI-14: DOSY of **4a** in THF-d_8 at RT.

Table SI-5: Experimental diffusion results for **4a**. $V_{vdW,exp}$ is the resulting V_{vdW} according to the model semi-empirical Stokes-Einstein model derived by us.

D_{ref} [m^2/s]	D_x [m^2/s]	$D_{x,norm}$ [m^2/s]	$V_{vdW,exp}$ [Å^3]
$2.649 \cdot 10^{-9}$	$9.921 \cdot 10^{-10}$	$8.710 \cdot 10^{-10}$	429.2

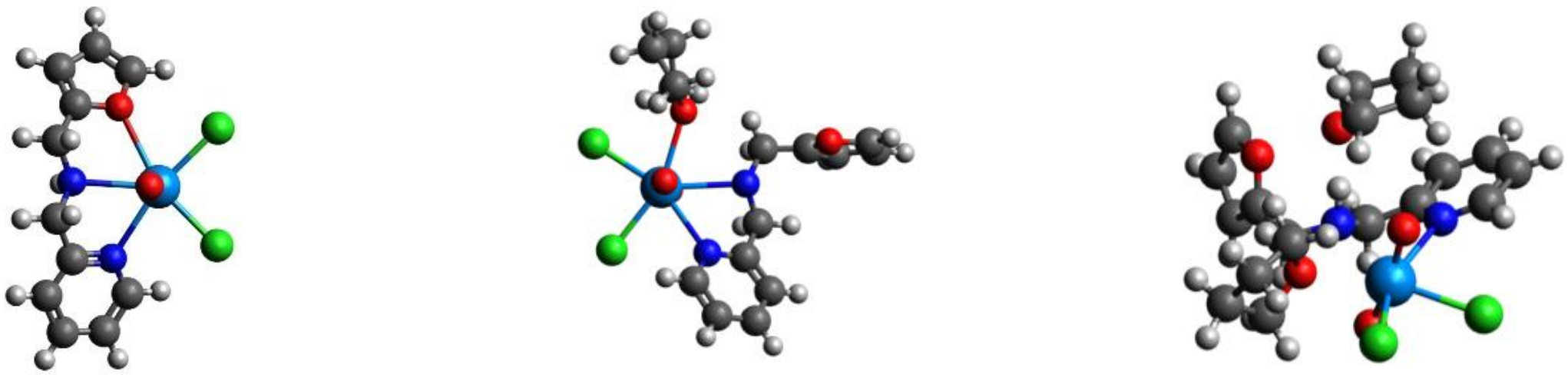

V_{vdW} = 285 Å^3<br>Δ = -33.7 %	V_{vdW} = 361 Å^3<br>Δ = -15.9 %	**V_{vdW} = 439 Å^3**<br>**Δ = 2.3 %**
M_{Stalke} = 774 g/mol<br>$Δ_{Stalke}$ = 46.3%	M_{Stalke} = 710 g/mol<br>$Δ_{Stalke}$ = 18.2 %	**M_{Stalke} = 665 g/mol**<br>**$Δ_{Stalke}$ = -1.2 %**

Figure SI-15: Model structures of **4a**, their V_{vdW} and the deviation Δ from $V_{vdW,exp}$. Below, their density corrected *Stalke* molar mass M_{Stalke} and the deviation from M $Δ_{Stalke}$ based on the MD_w model.

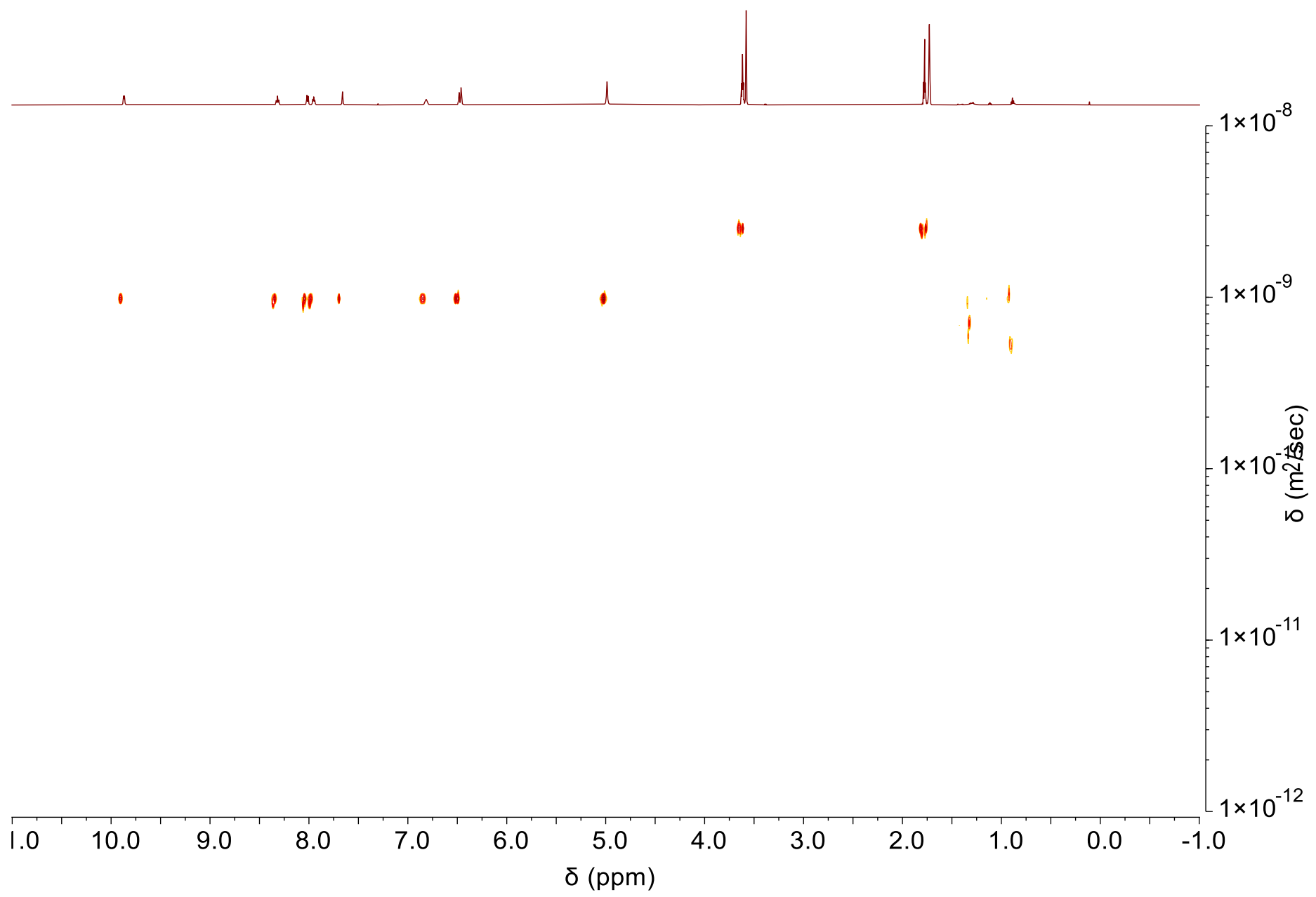

Figure SI-16: DOSY of **4b** in THF-d_8 at room temperature.

Table SI-6: Experimental diffusion results for **4b**. $V_{vdW,exp}$ is the resulting V_{vdW} according to the model semi-empirical Stokes-Einstein model derived by us.

D_{ref} [m^2/s]	D_x [m^2/s]	$D_{x,norm}$ [m^2/s]	$V_{vdW,exp}$ [Å^3]
$2.643 \cdot 10^{-9}$	$1.031 \cdot 10^{-9}$	$9.072 \cdot 10^{-10}$	399.7

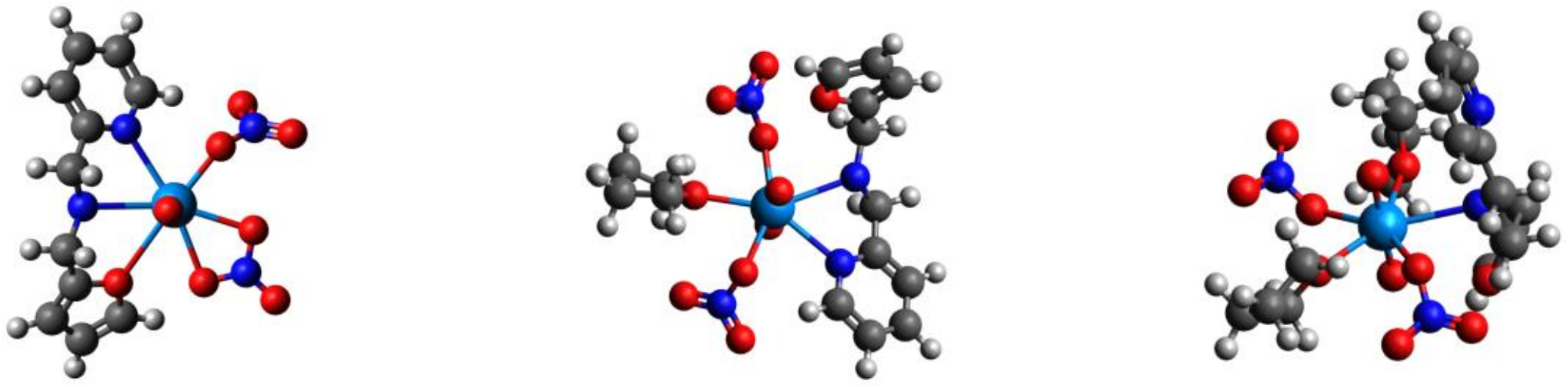

V_{vdW} = 315 Å³<br>Δ = -21.3 %	**V_{vdW} = 394 Å³<br>Δ = -1.5 %**	V_{vdW} = 471 Å³<br>Δ = 18.0%

M_{Stalke} = 707 g/mol<br>Δ_{Stalke} = 21.5%	**M_{Stalke} = 655 g/mol<br>Δ_{Stalke} = 0.1 %**	M_{Stalke} = 617 g/mol<br>Δ_{Stalke} = -15.1 %

Figure SI-17: Model structures of **4b** with none, one or two coordinated THF molecules, their V_{vdW} and the deviation Δ from $V_{vdW,exp}$. Below, their density corrected *Stalke* molar mass M_{Stalke} and the deviation from M Δ_{Stalke} based on the MD_w model.

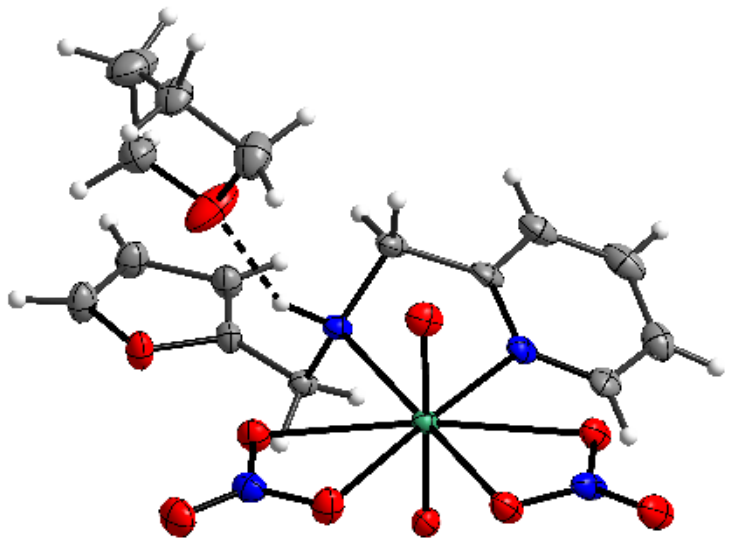

V_{vdW} = 389 Å³<br>Δ = -2.6 %

Figure SI-18: Crystal structure of **4b**, it's V_{vdW} and the deviation Δ from $V_{vdW,exp.}$

8 Benchmarking dataset

The .xyz files necessary as input for OVITO and MoloVol were generated from .cif files of the crystal structures if available or from force field geometry optimizations using Avogadro (section 18.1.1 Organic Molecules).

Table SI-7: Solvent independent properties; origin of the corresponding coordinates for the .xyz files is cited at the name of the molecule.

Molecule	r_g [Å]	V_{cov} [Å^3]	$V_{\Sigma vdW}$ [Å^3]	V_{vdW} [Å^3]
Cyclopentane[a]	1.42	10.4	188.5	87.9
Tetrahydrofuran[a]	1.34	7.5	165.0	79.0
Tetramethyl silane[a]	1.65	14.6	223.8	113.1
Methyl tert-butyl ether[a]	1.66	11.9	217.1	107.8
2,2,3,3-Tetramethylbutane[a]	1.93	17.0	316.1	150.5
Adamantane[a]	1.83	20.4	348.1	151.0
$N(SiMe_3)_3$ [31]	2.54	38.6	555.6	250.7
$Si(SiMe_3)_4$[a]	2.94	55.2	759.3	374.2
Toluene[a]	1.78	12.4	220.5	105.3
Diisopropyl ether[a]	2.00	14.0	254.8	125.2
Indene[32]	1.94	15.9	267.0	121.4
Naphthalene[33]	2.09	17.3	290.2	131.3
1,3-Indandione[a]	2.21	18.0	280.8	132.8
2-Phenylpyridine[a]	2.64	20.5	339.8	159.8
1,1,3,3-Tetramethoxypropane[a]	2.54	19.7	335.0	167.9
Diphenylacetylene[34]	3.51	23.6	397.6	180.8
Diphenyl sulfoxide[35]	2.71	26.9	393.5	188.2
1-Phenylnaphthaline[a]	2.96	27.6	458.5	208.9
Tri(o-tolyl)-phosphine[36]	3.21	42.6	668.5	302.1
Hexaphenylcyclotrisiloxane[37]	4.25	83.2	1227.8	572.1
BINAP[38]	4.36	85.8	1303.9	582.7
Anthracene[39]	2.73	24.1	397.6	173.1
Acridine[40]	2.69	23.8	386.3	170.0
9-Methylanthracene[41]	2.76	26.1	435.3	188.9
Pyrene[42]	2.60	27.3	444.0	188.4
Anthraquinone[43]	2.75	26.2	411.4	183.8
Triphenylene[44]	2.92	30.8	505.0	216.9
Tetraphenylnaphthaline[45]	3.94	58.4	963.5	411.6
$[Rh(CO)H(PPh_3)_3]$ [46]	4.30	123.7	1771.7	765.2
$[Rh(COD)Cl]_2$ [47]	3.04	63.6	717.6	313.8
$[Pd(OAc)_2(PPh_3)_2]$ [48]	4.09	96.4	1345.3	616.1
[(NHC)Pd(allyl)Cl] [49]	3.57	75.9	1113.3	496.1
$[RuCp(CO)_2]_2$ [50]	2.43	55.0	578.8	256.9
$[Li_2$(aminotrisphenolboryl)$]_2$ [51]	5.27	178.1	2552.9	1100.9
$[Sn(bpza)_2]$ [52]	3.32	71.7	1043.8	353.7
Bromoethane[a]	1.15	11.5	109.6	65.5
Allylbromide[a]	1.55	13.0	132.8	77.6
2-Bromopropene[a]	1.35	11.5	109.6	77.3
2-Bromopropane[a]	1.36	13.0	132.8	82.9
1-Bromopropane[a]	1.62	13.0	132.8	82.9
2-Bromo-2-butene (Z) [a]	1.57	13.5	140.1	94.9
2-Bromo-2-butene (E)[a]	1.70	13.5	140.1	94.8
2-Bromo-2-methylpropane[a]	1.49	15.0	170.5	117.3
1-Bromobutane[a]	2.05	15.0	170.5	100.3

Molecule	r_g [Å]	V_{cov} [Å^3]	$V_{\Sigma vdW}$ [Å^3]	V_{vdW} [Å^3]
2-Bromo-2-methylpropane[a]	1.83	15.7	185.0	117.3
1-Bromopentane[a]	2.49	15.7	185.0	117.4
Bromobenzene[a]	1.97	17.6	202.5	106.2
3-Bromopyridine[a]	1.96	17.4	191.2	102.6
Bromocyclohexane[a]	1.81	19.6	245.9	123.1
2-Bromostyrene[a]	2.19	21.1	263.4	135.6
2-(Bromoethyl)benzene[a]	2.82	21.8	277.9	140.7
4-Bromoanisole[a]	2.58	20.9	254.4	132.0
2-Bromomesitylene[a]	2.37	23.9	315.6	157.9
1-Bromonaphthalene[a]	2.43	24.4	309.9	152.9
6-Bromoquinoline[a]	2.70	24.2	298.6	148.6
1-Bromo-2,3,5,6-tetramethylbenzene[53]	2.40	26.0	353.3	165.4
1-Bromo-4-butylbenzene[a]	3.35	26.0	353.3	175.5
9-Bromophenanthrene[54]	2.88	31.2	417.3	191.9
9-Bromoanthracene[55]	2.75	31.2	417.3	192.8
Dibromomethane[a]	1.55	16.6	91.6	66.5
1,2-Dibromoethane[a]	2.16	18.7	129.3	83.8
1,2-Dibromopropane[a]	1.86	20.7	167.0	101.4
1,2-Dibromobenzene[a]	2.12	24.8	222.2	124.4
1,4-Dibromobenzene[a]	2.85	24.8	222.2	124.5
1,7-Dibromoheptane[a]	4.54	29.1	317.8	170.5
2,5-Dibromo-p-xylene[56]	2.84	27.3	297.6	152.9
Iodoethane[a]	1.08	15.6	118.2	73.8
Allyliodide[a]	1.47	17.0	141.4	85.8
2-Iodopropane[a]	1.29	17.6	155.9	91.0
1-Iodobutane[a]	1.96	19.7	193.6	108.6
2,2‘-Dimethyl-1-iodopropane[a]	1.79	20.0	208.1	125.5
1-Iodopentane[a]	2.41	20.0	208.1	125.8
Iodobenzene[a]	1.95	21.7	211.1	114.8
2-Iodotoluene[a]	2.02	23.7	248.8	131.9
4-Iodoaniline[57]	2.25	23.3	237.5	122.7
4-Iodoanisole[58]	2.56	24.9	263.0	138.0
6-Iodoquinoline[a]	2.74	28.2	307.2	157.0
$[(Cp^*)Rh(Cl)_2]_2$ [59]	3.17	76.8	904.4	431.3
$[Rh_2Cl_2(C_2H_4)_4]$ [60]	2.20	47.9	473.8	215.6
Grubbs 1st gen. $[RuCl_2(PCy_3)_2(CHPh)]$ [61]	4.28	111.2	1690.3	569.9
Grubbs 2nd gen. $[RuCl_2(PCy_3)(NHC)]$ [62]	4.27	114.8	1718.9	565.7
[(Zn(aminotrisphenolboryl))$_2$(tmeda)] [51]	4.84	148.1	2306.3	986.6
[Zn(aminobisphenolboryl)(^{n}PrAcAc)]$_2$ [51]	5.11	142.7	2203.3	964.0
[^{i}Bu$_2$Al(OC(Me)$_2$C(Me)$_2$O)B(H)(tmp)]$_2$ [63]	5.93	124.4	2026.5	884.4
[^{i}Bu$_2$Al(OC(Me)$_2$C(Me)$_2$O)B(H)(hmds)]$_2$ [63]	6.07	133.3	2063.1	926.6
Na-1,2-tBu-DH(DMAP)-Me$_6$TRE [64]	3.74	75.7	1069.3	505.5
[Al(Cp*)Ph$_2$] [65]	3.25	46.4	739.7	326.2
[Al(Cp*)(o-tolyl)$_2$] [65]	3.25	50.6	815.1	357.6
[Al(Cp*)(naphthyl)$_2$] [65]	3.66	59.9	954.5	414.6
[Al(Cp*)(thiophenyl)$_2$] [65]	3.13	49.1	674.4	312.6
[(o-tolyl)$_2$Al(CO$_2$)Cp*]$_2$ [65]	4.50	106.8	1733.2	759.9
[(PhF)$_2$Al(CO$_2$)Cp*]$_2$ [65]	4.84	103.5	1605.6	726.9
[(thiophenyl)$_2$Al(CO$_2$)Cp*]$_2$ [65]	4.34	106.2	1451.8	679.1
CpTePh$_3$ [66]	3.02	51.0	712.0	328.5

[a] *generated by universal force field calculations*

Table SI-8: Solvent dependent properties; source of the normalized *D* is given at the name of the molecule.

	in THF-d₈		*in C₆D₆*		*in Tol-d₈*	
**Molecule**	$D \cdot 10^{-9}$ **[m²/s]**	V_C **[Å³]**	$D \cdot 10^{-9}$ **[m²/s]**	V_C **[Å³]**	$D \cdot 10^{-9}$ **[m²/s]**	V_C **[Å³]**
Cyclopentane [15]	2.27	109.6			2.141	109.6
Tetrahydrofuran [15]	1.99	101.1			2.126	101.1
Tetramethylsilane [15]	1.80	113.1			1.801	136.3
Methyl tert-butyl ether [15]	2.00	128.8			1.797	128.8
2,2,3,3-Tetramethylbutane [15]	1.68	158.7			1.599	158.7
Adamantane [15]	1.55	157.0			1.428	157.0
$N(SiMe_3)_3$ [15]	1.24	237.3			1.129	237.3
$Si(SiMe_3)_4$ [15]	1.05	306.6			0.991	306.6
Toluene [15]	2.12	105.3				109.8
Diisopropyl ether [15]	1.89	105.3			1.773	151.2
Indene [15]	1.85	121.4			1.699	141.2
Naphthalene [15]	1.79	149.2			1.610	149.2
1,3-Indandione [15]	1.55	153.5			1.426	153.5
2-Phenylpyridine [15]	1.59	179.6			1.388	179.6
1,1,3,3-Tetramethoxypropane [15]	1.50	194.5			1.342	194.5
Diphenylacetylene [15]	1.40	207.4			1.232	194.3
Diphenyl sulfoxide [15]	1.33	204.6			1.186	204.6
1-Phenylnaphthaline [15]	1.31	222.3			1.207	222.3
Tri(o-tolyl)-phosphine [15]	1.01	296.2			0.904	296.2
Hexaphenylcyclotrisiloxane [15]	7.20	531.2			0.619	531.2
BINAP [15]	6.82	513.6			0.591	513.6
Anthracene [15]	1.54	187.4			1.389	187.4
Acridine [15]	1.51	186.0			1.319	186.0
9-Methylanthracene [15]	1.43	200.6			1.311	200.6
Pyrene [15]	1.43	196.6			1.271	196.6
Anthraquinone [15]	1.37	196.6			1.192	196.6
Triphenylene [15]	1.30	223.5			1.109	223.5
1,2,3,4-Tetraphenylnaphthalene [15]	7.85	387.4			0.682	387.4
$[Rh(CO)H(PPh_3)_3]$ [18]	0.661	590.3	0.583	590.3		

Molecule	in THF-d_8		in C_6D_6		in Tol-d_8	
	**$D \cdot 10^{-9}$ [m²/s]**	**V_C [Å³]**	**$D \cdot 10^{-9}$ [m²/s]**	**V_C [Å³]**	**$D \cdot 10^{-9}$ [m²/s]**	**V_C [Å³]**
$[Rh(COD)Cl]_2$ [18]	1.00	290.3	0.933	290.3		
$[Pd(OAc)_2(PPh_3)_2]$ [18]	0.697	527.3	0.666	527.3		
[(NHC)Pd(allyl)Cl] [18]	0.805	413.5	0.745	413.5		
$[RuCp(CO)_2]_2$ [18]	1.08[67]	242.0	0.107	242.0		
$[Li_2$(aminotrisphenolboryl)$]_2$ [51]	0.526	794.9				
$[Sn(bpza)_2]$ [51]	9.32	347.3				
Bromoethane [17]	2.68	89.7	2.55	89.7		
Allylbromide [17]	2.34	102.5	2.42	102.5		
2-Bromopropene [17]	2.44	101.7	2.34	101.7		
2-Bromopropane [17]	2.23	107.0	2.23	107.0		
1-Bromopropane [17]	2.33	108.6	2.32	108.6		
2-Bromo-2-butene (*Z*) [17]	2.15	120.2	2.15	120.2		
2-Bromo-2-butene (*E*) [17]	2.13	119.5	2.17	119.5		
2-Bromo-2-methylpropane [17]	1.96	136.7	1.90	121.3		
1-Bromobutane [17]	2.11	127.5	2.18	127.5		
2-Bromo-2-methylpropane [17]	1.81	136.7	1.90	136.7		
1-Bromopentane [17]	1.93	146.4	1.97	146.4		
Bromobenzene [17]	1.93	127.9	1.96	127.9		
3-Bromopyridine [17]	1.95	124.3	1.97	124.3		
Bromocyclohexane [17]	1.81	139.2	1.84	139.2		
2-Bromostyrene [17]	1.74	156.4	1.69	156.4		
2-(Bromoethyl)benzene [17]	1.64	163.7	1.57	163.7		
4-Bromoanisole [17]	1.82	157.0	1.76	157.0		
2-Bromomesitylene [17]	1.78	179.0	1.56	179.0		
1-Bromonaphthalene [17]	1.58	170.7	1.54	170.7		
6-Bromoquinoline [17]	1.53	168.5	1.58	168.5		
1-Bromo-2,3,5,6-tetramethylbenzene [17]	1.51	182.4	2.55	182.4		
1-Bromo-4-butylbenzene [17]	1.51	202.3	2.42	202.3		
9-Bromophenanthrene [17]	1.36	204.1	2.34	204.1		
9-Bromoanthracene [17]	1.39	204.9	2.23	204.9		
Dibromomethane [17]	2.29	89.8	2.32	89.8		
1,2-Dibromoethane [17]	1.98	108.5	2.15	108.5		
1,2-Dibromopropane [17]	1.85	124.8	2.17	124.8		
1,2-Dibromobenzene [17]	1.65	144.8	1.90	144.8		
1,4-Dibromobenzene [17]	1.74	147.5	2.18	147.5		

	in THF-d$_8$		*in C_6D_6*		*in Tol-d$_8$*	
**Molecule**	***D·10^{-9}* [m^2/s]**	***V$_C$* [Å^3]**	***D·10^{-9}* [m^2/s]**	***V$_C$* [Å^3]**	***D·10^{-9}* [m^2/s]**	***V$_C$* [Å^3]**
1,7-Dibromoheptane [17]	1.46	203.8	1.90	203.8		
2,5-Dibromo-p-xylene [17]	1.55	172.8	1.97	172.8		
Iodoethane [17]	2.40	96.6	1.96	96.6		
Allyliodide [17]	2.24	109.9	1.97	109.9		
2-Iodopropane [17]	2.20	113.3	1.84	113.3		
1-Iodobutane [17]	1.93	134.9	1.69	134.9		
2,2'-Dimethyl-1-iodopropane [17]	1.91	142.6	1.57	142.6		
1-Iodopentane [17]	1.83	153.9	1.76	153.9		
Iodobenzene [17]	1.79	135.9	1.56	135.9		
2-Iodotoluene [17]	1.69	152.0	1.54	152.0		
4-Iodoaniline [17]	1.54	143.9	1.58	143.9		
4-Iodoanisole [17]	1.63	160.0	1.49	160.0		
6-Iodoquinoline [17]	1.50	176.2	1.53	176.2		
$[(Cp^*)Rh(Cl)_2]_2$ [18]			1.32	366.2		
$[Rh_2Cl_2(C_2H_4)_4]$ [18]			1.35	215.6		
Grubbs 1st gen. $[RuCl_2(PCy_3)_2(CHPh)]$ [18]			2.35	569.9		
Grubbs 2nd gen. $[RuCl_2(PCy_3)(NHC)]$ [18]			2.12	565.7		
[(Zn(aminotrisphenolboryl))$_2$(tmeda)] [18]			1.98	731.7		
[Zn(aminobisphenolboryl)(^{n}PrAcAc)]$_2$ [18]			1.72	761.7		
[^{i}Bu$_2$Al(OC(Me)$_2$C(Me)$_2$O)B(H)(tmp)]$_2$ [63]			1.70	702.8		
[^{i}Bu$_2$Al(OC(Me)$_2$C(Me)$_2$O)B(H)(hmds)]$_2$ [63]			1.42	744.5		
Na-1,2-tBu-DH(DMAP)-Me$_6$TRE [64]			0.75	565.2		
[Al(Cp*)Ph$_2$] [65]			0.91	365.6		
[Al(Cp*)(o-tolyl)$_2$] [65]			0.92	407.4		
[Al(Cp*)(naphthyl)$_2$] [65]			0.81	475.0		
[Al(Cp*)(thiophenyl)$_2$] [65]			0.94	351.2		
[(o-tolyl)$_2$Al(CO$_2$)Cp*]$_2$ [65]			0.61	947.6		
[(PhF)$_2$Al(CO$_2$)Cp*]$_2$ [65]			0.63	904.2		
[(thiophenyl)$_2$Al(CO$_2$)Cp*]$_2$ [65]			0.65	825.8		
CpTePh$_3$ [66]			0.93	372.2		

9 Holtz Viscosity Adjustment

The viscosities of all solvents were adjusted using the method of Holtz *et al.* (eq. (5)).[68]

$$\frac{\eta_D}{\eta_H} = \frac{M_H + M_D}{2M_H} \tag{5}$$

with η_D and M_D being the dynamic viscosity of the deuterated solvents and its molar mass and η_H and M_H the respective value for non-deuterated solvents.

Table SI-9: Viscosity of the non-deuterated solvents and Holtz-adjusted viscosity of deuterated solvents.

Solvent	η_H [mPa·s]	η_D [mPa·s]
THF-d_8	0.460	0.486
C_6D_6	0.560	0.584
Tol-d_8	0.603	0.626

10 Calculation of Errors

The relative errors Δ_{rel} of the models are calculated using the following approach:

$$\Delta_{\mathrm{rel}} = \frac{x_m - x}{x} \tag{6}$$

with x_m the model determined value and x the expected (experimental) value. Based on the assumption of a dataset with normal-distributed errors, all models are evaluated using the 2σ error. σ is the standard deviation according to:

$$\sigma = \sqrt{\frac{1}{n}\sum_{i=1}^{n}(\Delta_{\mathrm{rel}} - \overline{\Delta}_{\mathrm{rel}})^2} \tag{7}$$

with $\overline{\Delta}_{\mathrm{rel}}$ the mean of the relative error.

11 Power Laws inspired by the Gierer-Wirtz Model – Testing for the Right Volume Descriptor

All scatter plots were fitted using the following power law relation:

$$D = ax^b \quad (8)$$

with *a* and *b* the fit-parameters, and *x* the independent variable. The Levenberg Marquardt iteration algorithm of Originlab 2023b was used for the fitting process.

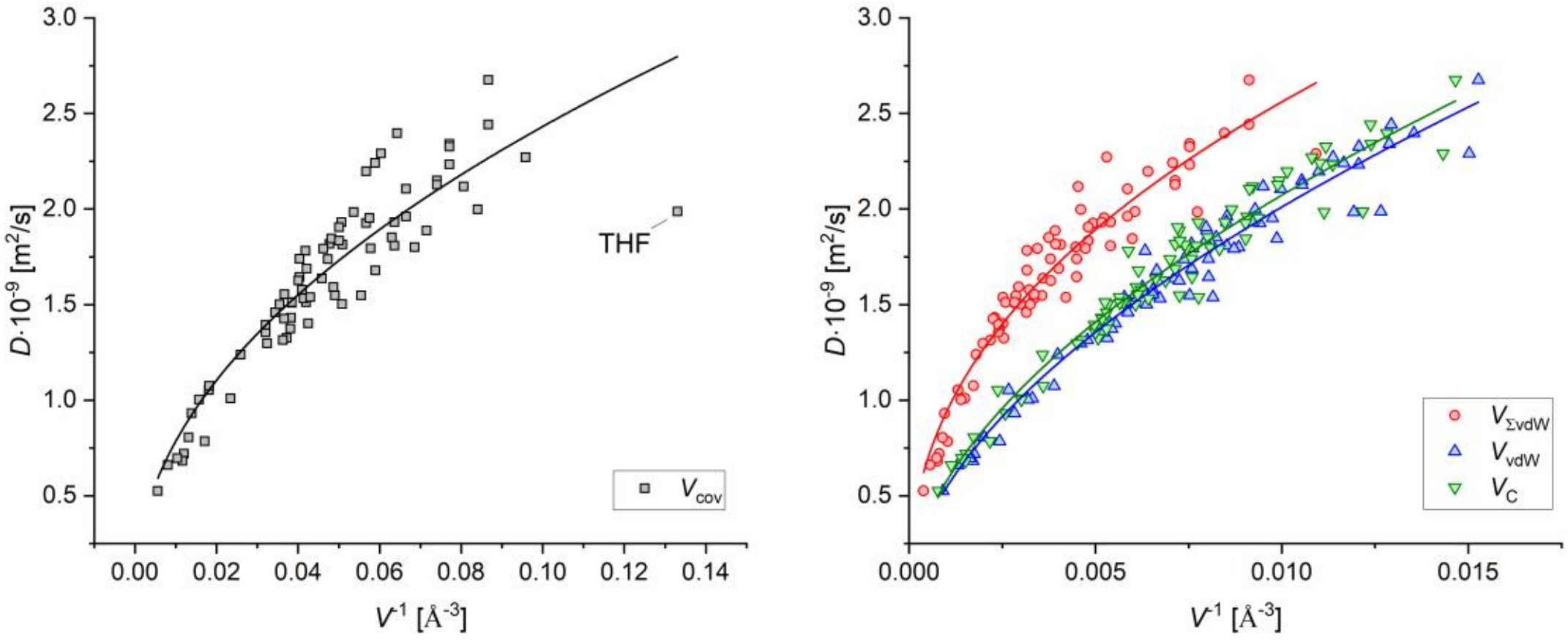

Figure SI-19: Scatter-plots in THF-d_8 for different volume descriptors. The lines represent the resulting fit-functions according to equation (8).

Table SI-10: Fitting parameters for the different volume descriptors in THF-d_8. $\bar{\Delta}_{rel}$ is the mean error and 2σ the double standard deviation of the error distribution.

	$a \cdot 10^{-9}$ [m²s⁻¹Å³]	b	R^2	$\bar{\Delta}_{rel}$	2σ
V_{cov}	7.53	0.49	0.845	0.02	0.22
$V_{\Sigma vdW}$	19.10	0.44	0.932	-0.01	0.16
V_{vdW}	27.67	0.57	0.958	0.00	0.11
V_C	26.99	0.56	0.954	-0.01	0.12

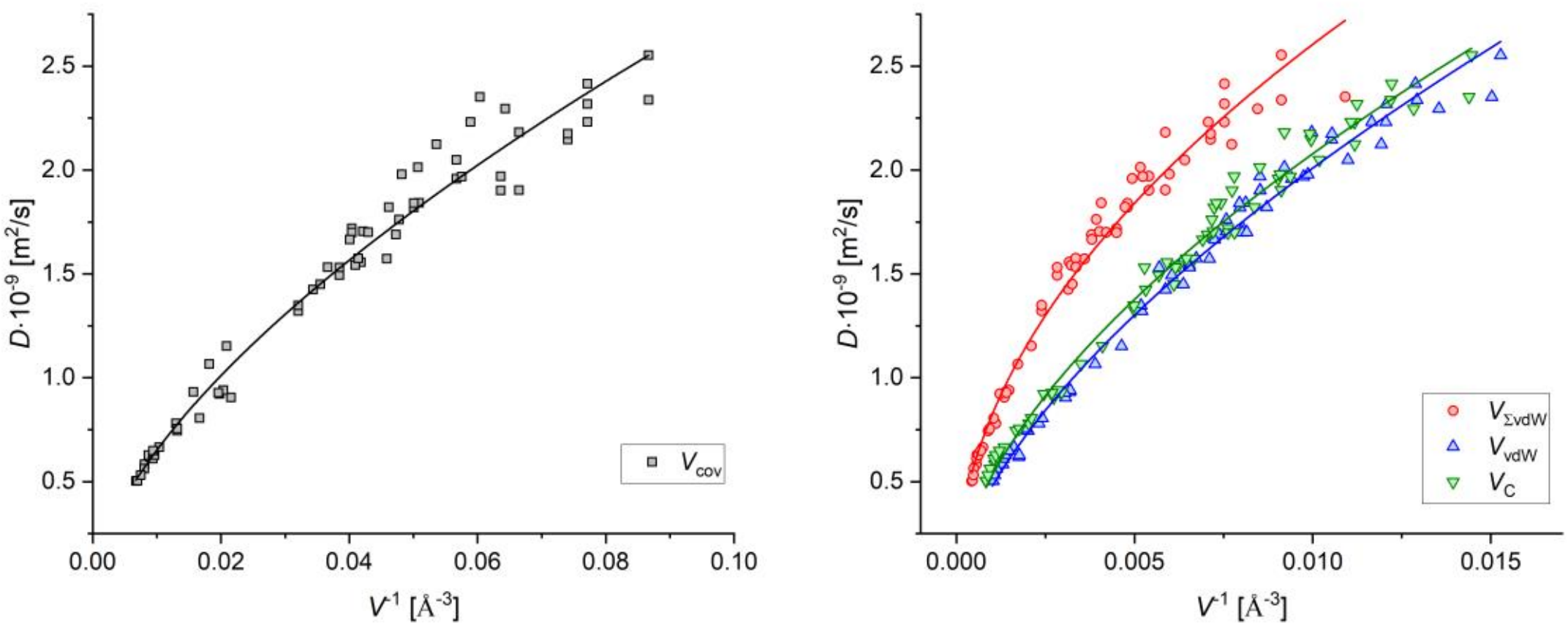

Figure SI-20: Scatter plots in C_6D_6 for the different volume descriptors. The lines represent the resulting fit-functions according to equation (8).

Table SI-11: Fitting parameters for the volume descriptors in C_6D_6. $\overline{\Delta}_{\mathrm{rel}}$ is the mean error and 2σ the double standard deviation of the error distribution.

	$a \cdot 10^{-8}$ [$m^2s^{-1}Å^3$]	b	R^2	$\overline{\Delta}_{\mathrm{rel}}$	2σ
V_{cov}	1.12	0.63	0.956	0.01	0.13
$V_{\Sigma\,\mathrm{vdW}}$	2.62	0.50	0.975	0.00	0.11
V_{vdW}	3.59	0.62	0.984	-0.03	0.09
V_{C}	2.94	0.58	0.982	0.01	0.10

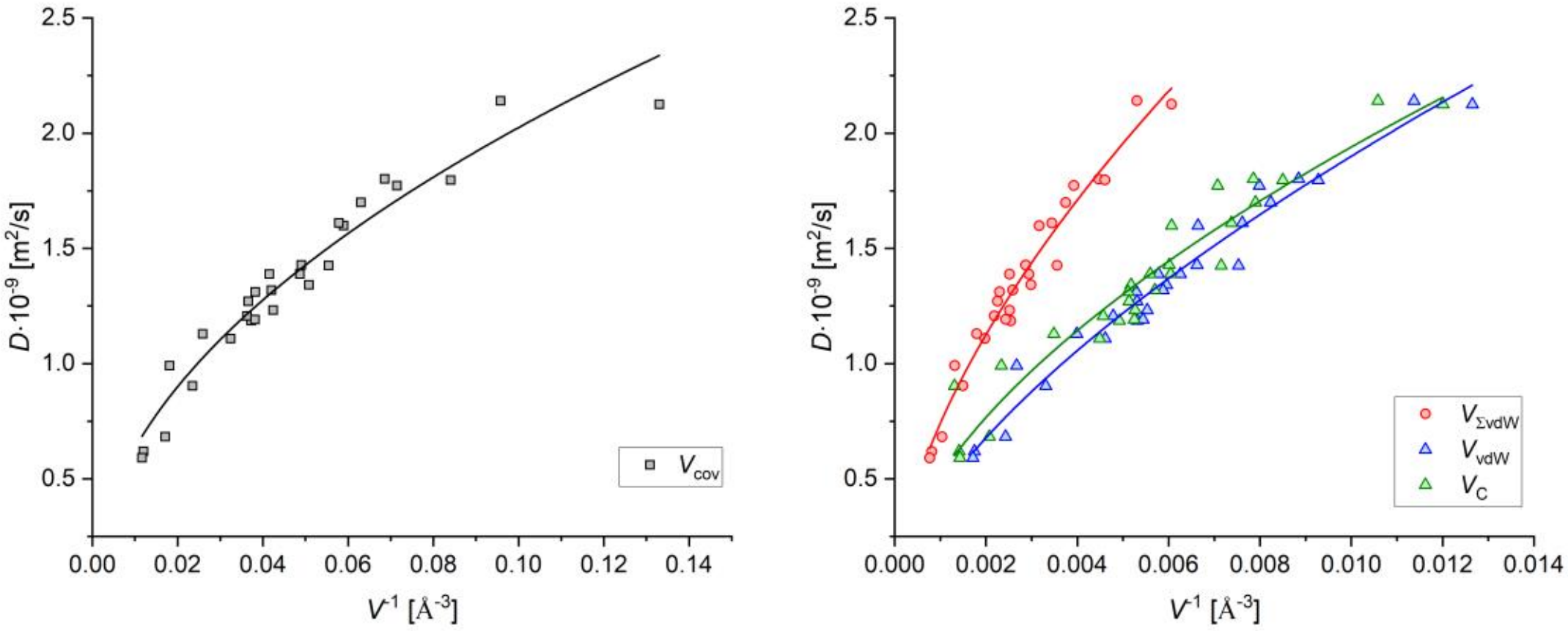

Figure SI-21: Scatter plots in Tol-*d*8 for the different volume descriptors. The lines represent the resulting fit-functions according to equation (8).

Table SI-12: Fitting parameters for the different volume descriptors in Tol-*d*8. $\overline{\Delta}_{\mathrm{rel}}$ is the mean error and 2σ the double standard deviation of the error distribution.

	$a \cdot 10^{-9}$ [$m^2s^{-1}Å^3$]	b	R^2	$\overline{\Delta}_{\mathrm{rel}}$	2σ
V_{cov}	6.46	0.50	0.946	0.03	0.16
$V_{\Sigma\,\mathrm{vdW}}$	47.57	0.60	0.962	0.02	0.12
V_{vdW}	36.27	0.64	0.963	0.01	0.12
V_{C}	27.66	0.58	0.923	0.00	0.14

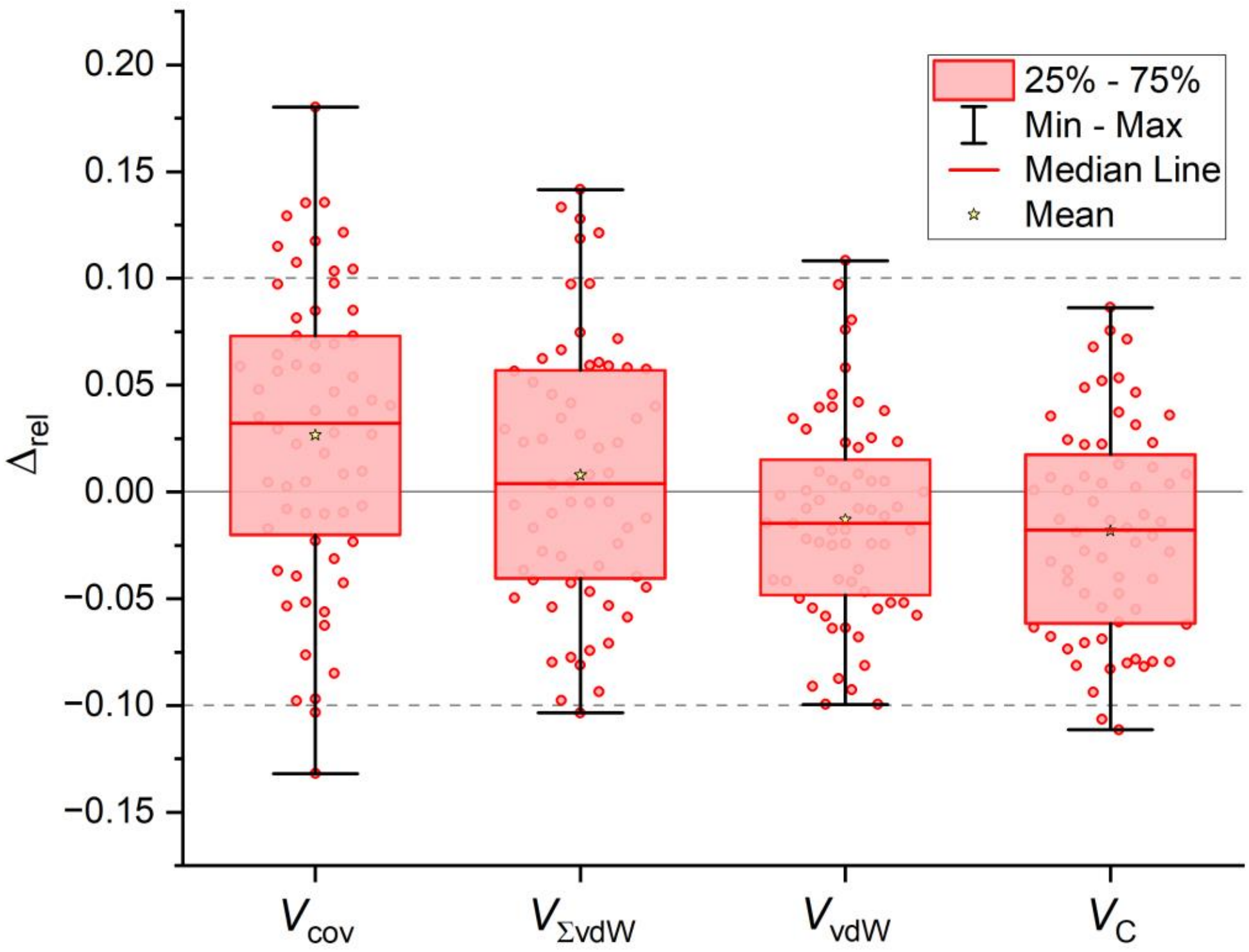

Figure SI-22: Box plots of relative error distributions of the empirical volume-based diffusion models based on different volume descriptors for diffusion in C_6D_6 at 25 °C.

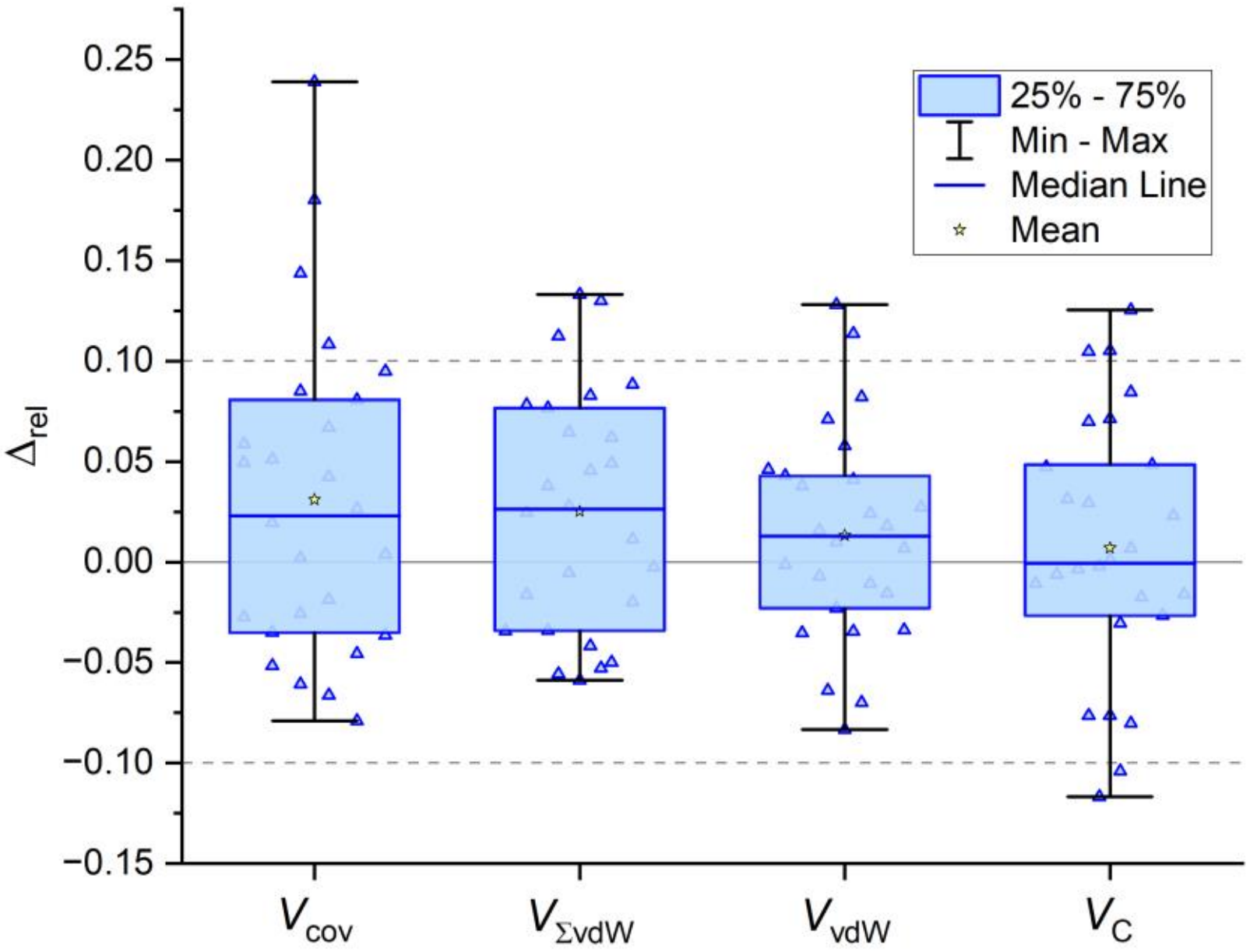

Figure SI-23: Box plots of relative error distributions of the empirical volume-based diffusion models based on different volume descriptors for diffusion in Tol-d_8 at 25 °C.

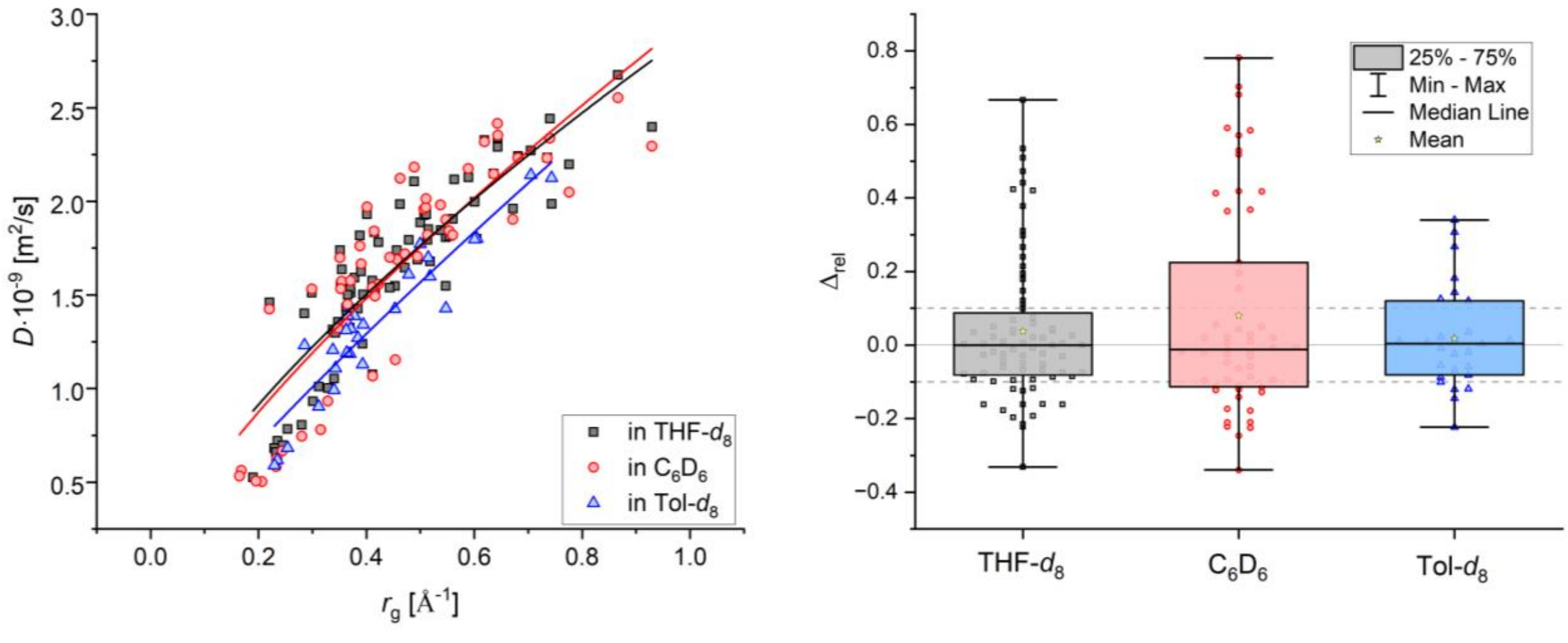

Figure SI-24: (Left) Empirical model of diffusion using a power law based on r_g at 25 °C. (Right) Box plot of error distribution of empirical diffusion models using r_g as a characteristic variable at 25 °C.

Table SI-13: Parameters of the r_g fits at 25 °C. $\overline{\Delta}_{rel}$: mean error, 2σ: double standard deviation of the error distribution.

	$a\cdot10^{-9}$ [$m^2s^{-1}Å^3$]	b	R^2	$\overline{\Delta}_{rel}$	2σ
THF-d_8	2.90	0.72	0.78	0.04	0.39
C_6D_6	2.97	0.76	0.76	0.08	0.56
Tol-d_8	2.85	0.87	0.88	0.02	0.27

12 Gierer-Wirtz Models

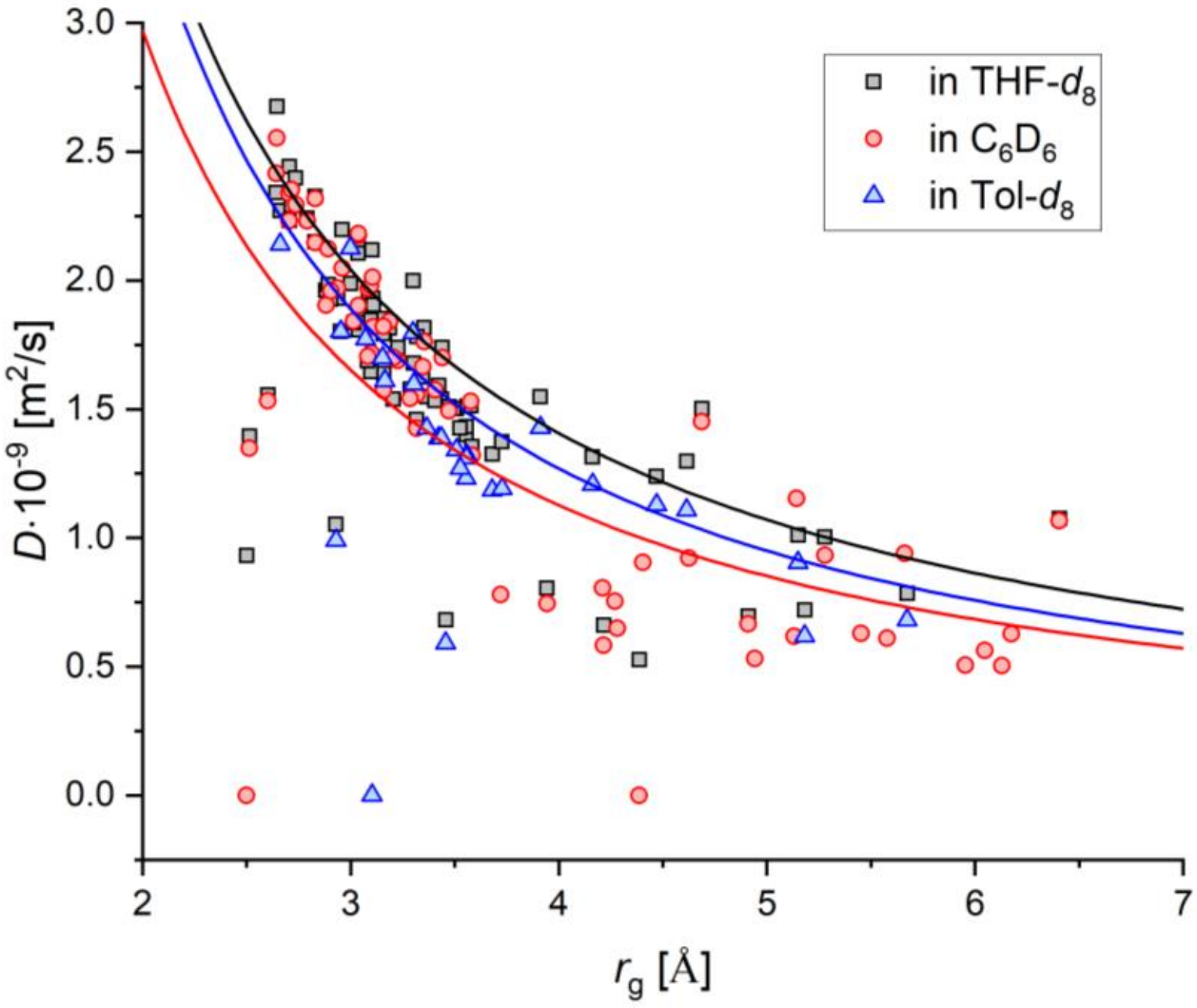

Figure SI-25: Normalized experimental *D* vs the r_g in the respective solvents. Solid lines depict the *Gierer-Wirtz* model based on r_g for the given conditions.

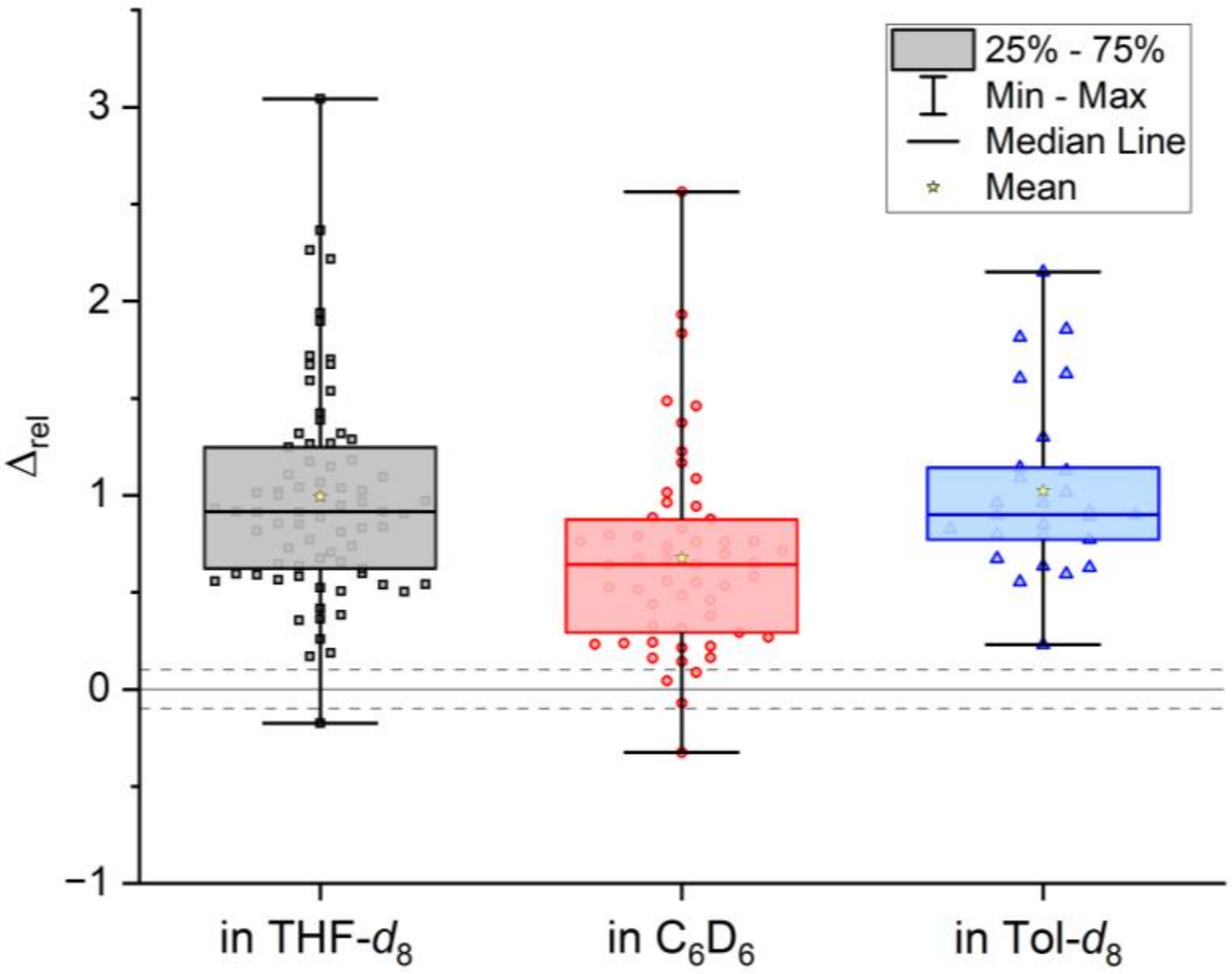

Figure SI-26: Box plot of error distribution of Gierer-Wirtz model based on r_g for diffusion at 25 °C.

Table SI-14: $\overline{\Delta}_{\mathrm{rel}}$: mean error and 2σ: double standard deviation of the error distribution of the Gierer-Wirtz models based on r_g.

	$\overline{\Delta}_{\mathrm{rel}}$	2σ
THF-d_8	0.99	1.08
C_6D_6	0.67	1.02
Tol-d_8	1.02	0.87

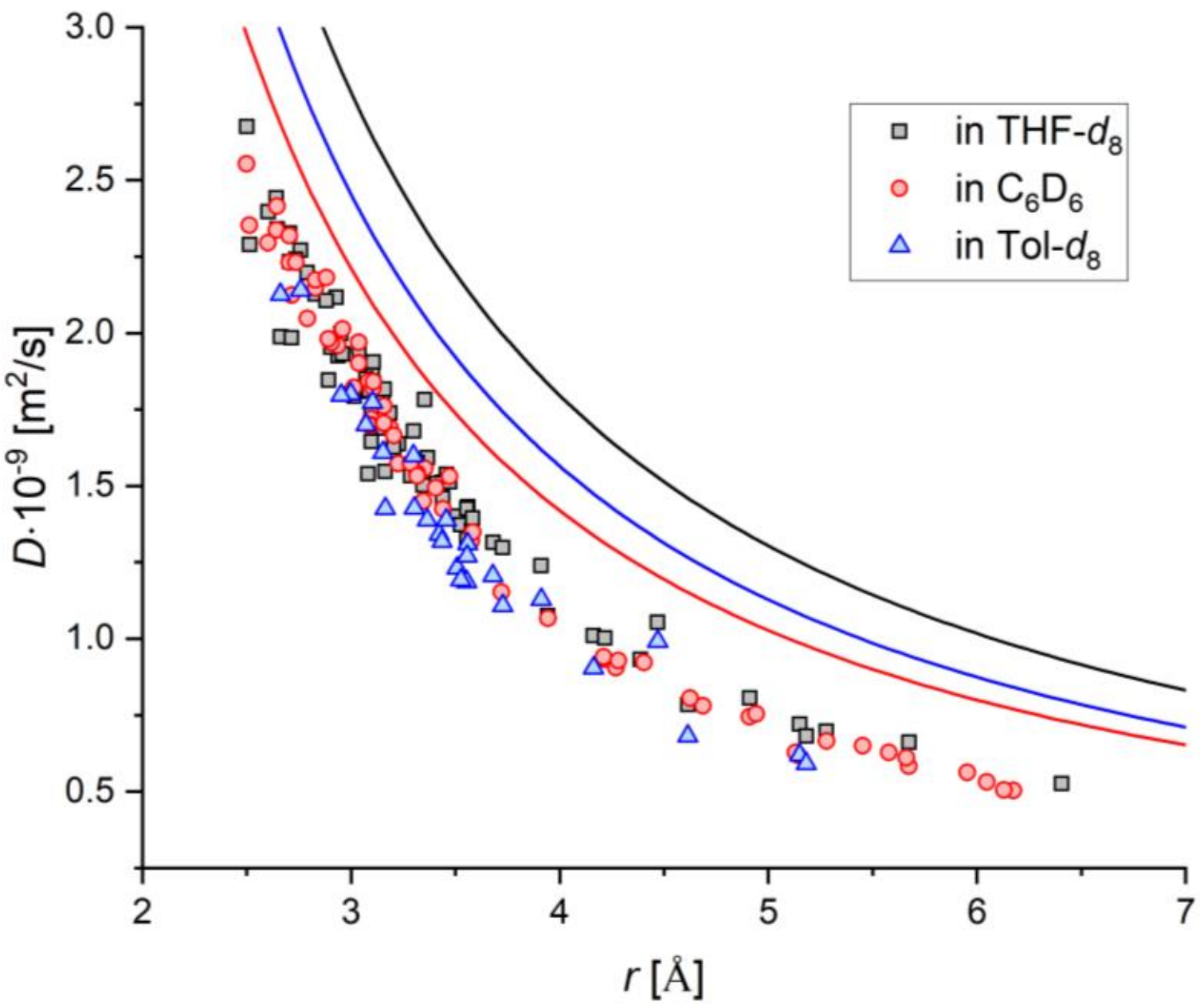

Figure SI-27: Normalized experimental *D* vs the radius parameter *r* in the respective solvents. *r* is derived by the assumption of a sphere and V_{vdW}. Solid lines depict the *Gierer-Wirtz* model based on *r* for the given conditions.

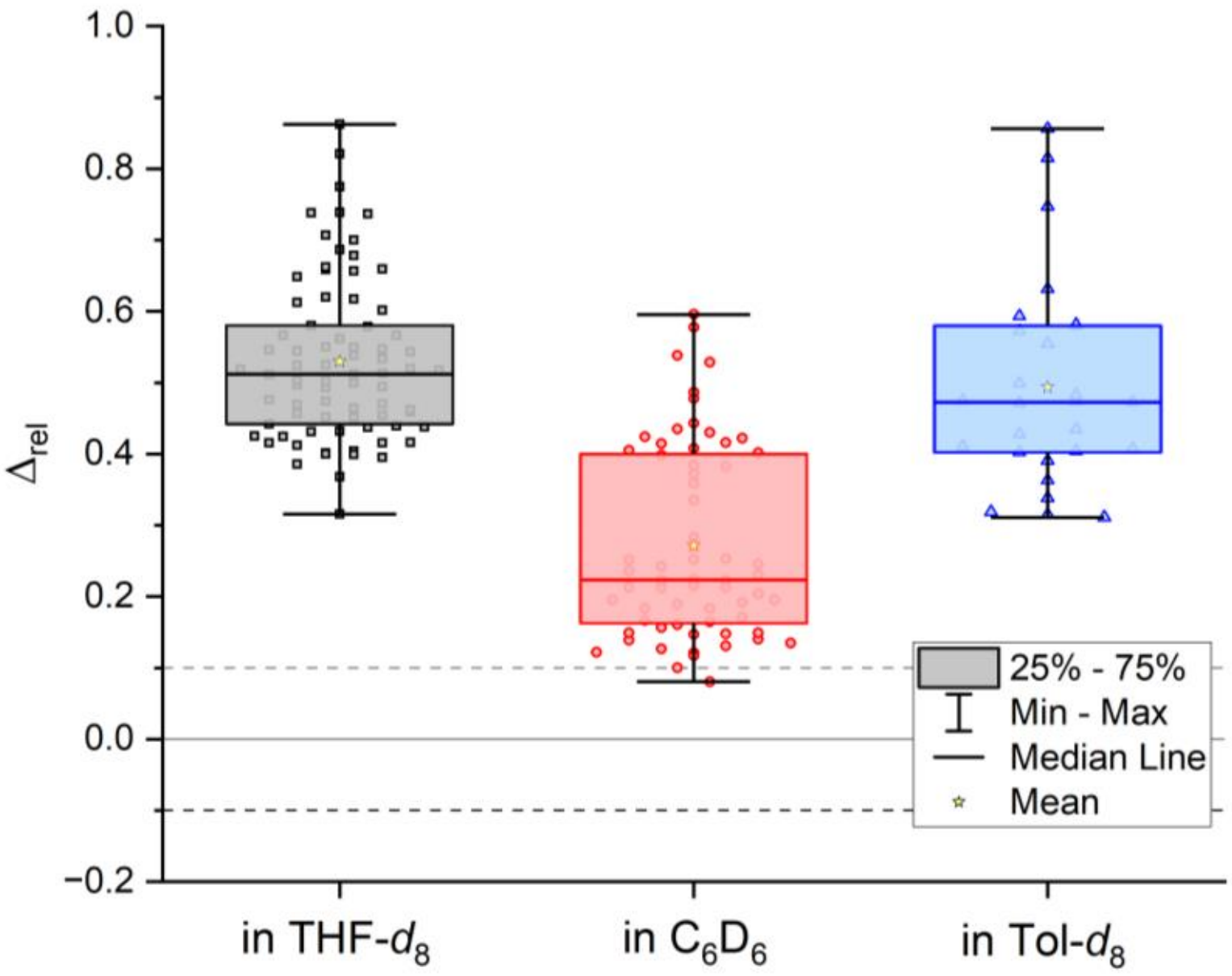

Figure SI-28: Box plot of error distribution of Gierer-Wirtz model based on V_{vdW} for diffusion at 25 °C.

Table SI-15: $\overline{\Delta}_{rel}$: mean error and 2σ: double standard deviation of the error distribution of the Gierer-Wirtz models based on V_{vdW}.

	$\overline{\Delta}_{rel}$	2σ
THF-d_8	0.53	0.22
C_6D_6	0.27	0.27
Tol-d_8	0.49	0.28

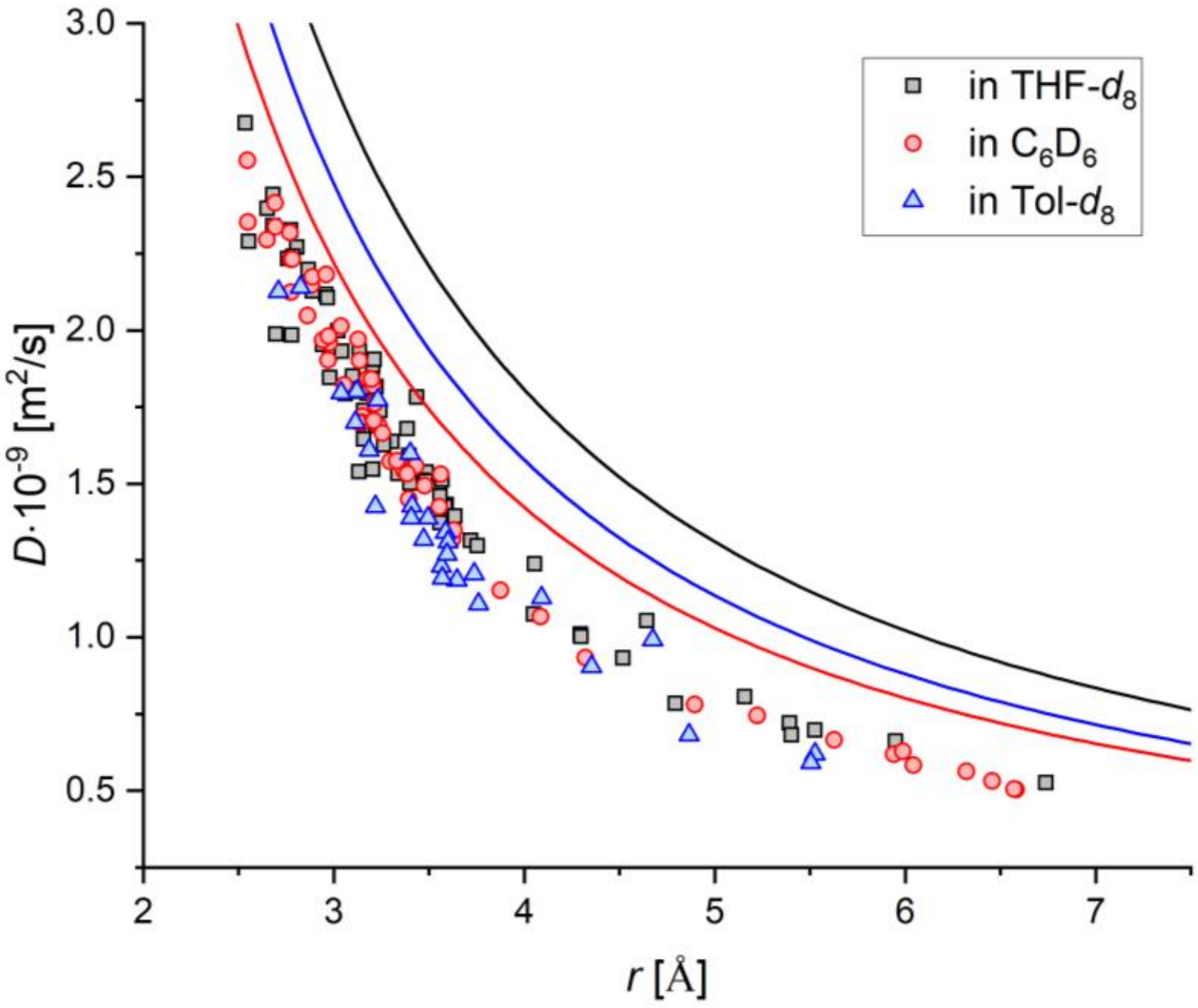

Figure SI-29: Normalized experimental *D* vs the radius parameter *r* in the respective solvents. *r* is derived by the assumption of a sphere and V_C. Solid lines depict the *Gierer-Wirtz* model based on *r* for the given conditions.

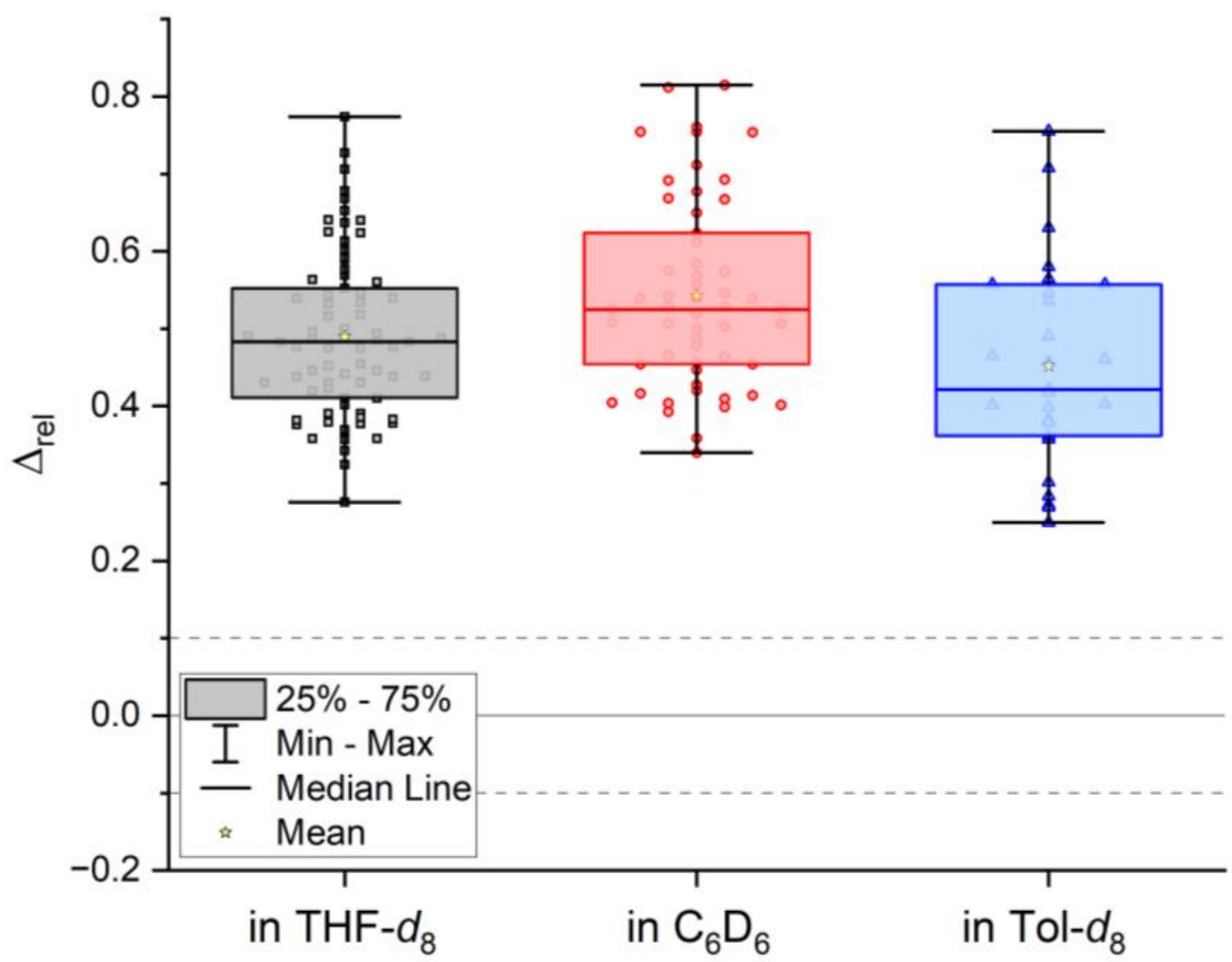

Figure SI-30: Box plot of error distributions of Gierer-Wirtz model based on V_C for diffusion at 25 °C.

Table SI-16: $\overline{\Delta}_{\mathrm{rel}}$: mean error and 2σ: double standard deviation of the error distribution of the *Gierer-Wirtz* models based on V_C.

	$\overline{\Delta}_{\mathrm{rel}}$	2σ
THF-d_8	0.49	0.21
C_6D_6	0.20	0.19
Tol-d_8	0.45	0.26

13 Chen and Chen Model

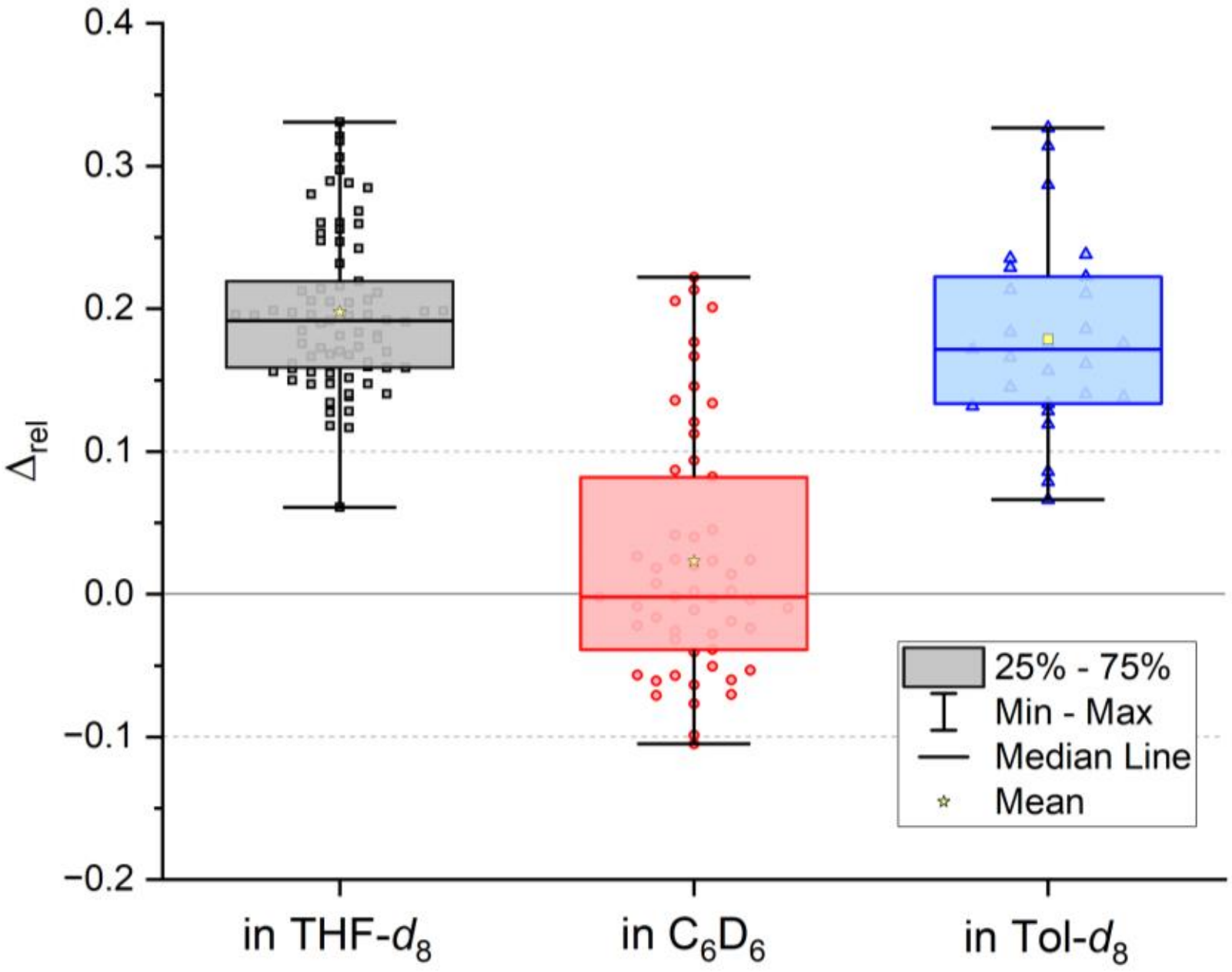

Figure SI-31: Box plot of error distributions of the *Chen-Chen* model based on V_{vdW} for diffusion at 25 °C.

Table SI-17: $\overline{\Delta}_{rel}$: mean error and 2σ: double standard deviation of the error distributions of the *Chen-Chen* models based on V_{vdW}.

	$\overline{\Delta}_{rel}$	2σ
THF-d_8	0.20	0.11
C_6D_6	0.02	0.17
Tol-d_8	0.18	0.13

14 Spernol-Plot Power-Law fits

The *Spernol*-type scatter plots were fitted using the following power-law relation:

$$f_{\mathrm{t}} = a \cdot a_{\mathrm{GW}}{}^{b} \tag{9}$$

with *a* and *b* the fit-parameters. The Levenberg Marquardt iteration algorithm of Originlab 2023b was used for the fitting process. f_{t} was derived from experimental *D* as follows:

$$f_{\mathrm{t}} = \frac{k_{\mathrm{B}}T}{6\pi\eta r D} \tag{10}$$

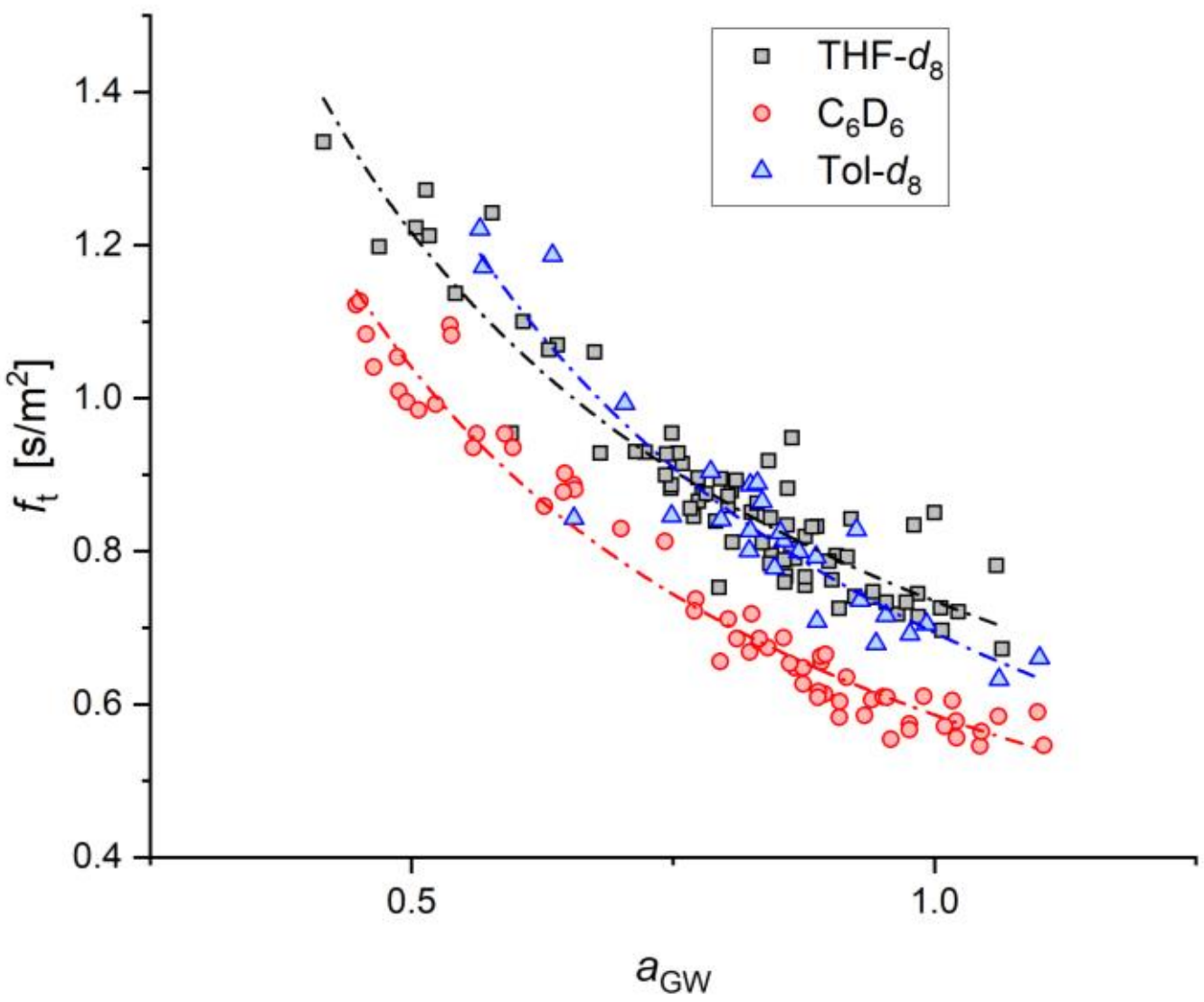

Figure SI-32: Scatterplots of f_{t} vs a_{GW}. a_{GW} is based on V_{vdW}. The lines represent the resulting fit-functions according to equation (9).

Table SI-18: Parameters of the *Spernol* type power-law at 25°C. $\overline{\Delta}_{\mathrm{rel}}$: mean error, 2σ: double standard deviation of the error distribution.

	a·[m^{2}s^{-1}]	*b*	R^2	$\overline{\Delta}_{\mathrm{rel}}$	2σ
THF-d_8	0.735	-073	0.882	0.00	0.11
C_6D_6	0.586	-0.83	0.959	0.00	0.09
Tol-d_8	0.694	-0.94	0.857	0.22	0.46

15 Hiller, Grabe and Schonert Model

The molar mass correlation by *Hiller*, *Grabe* and *Schonert* is based on the *Mark-Houwink* relation and thus can be seen as standing in the tradition of the *Stalke* models.[69]

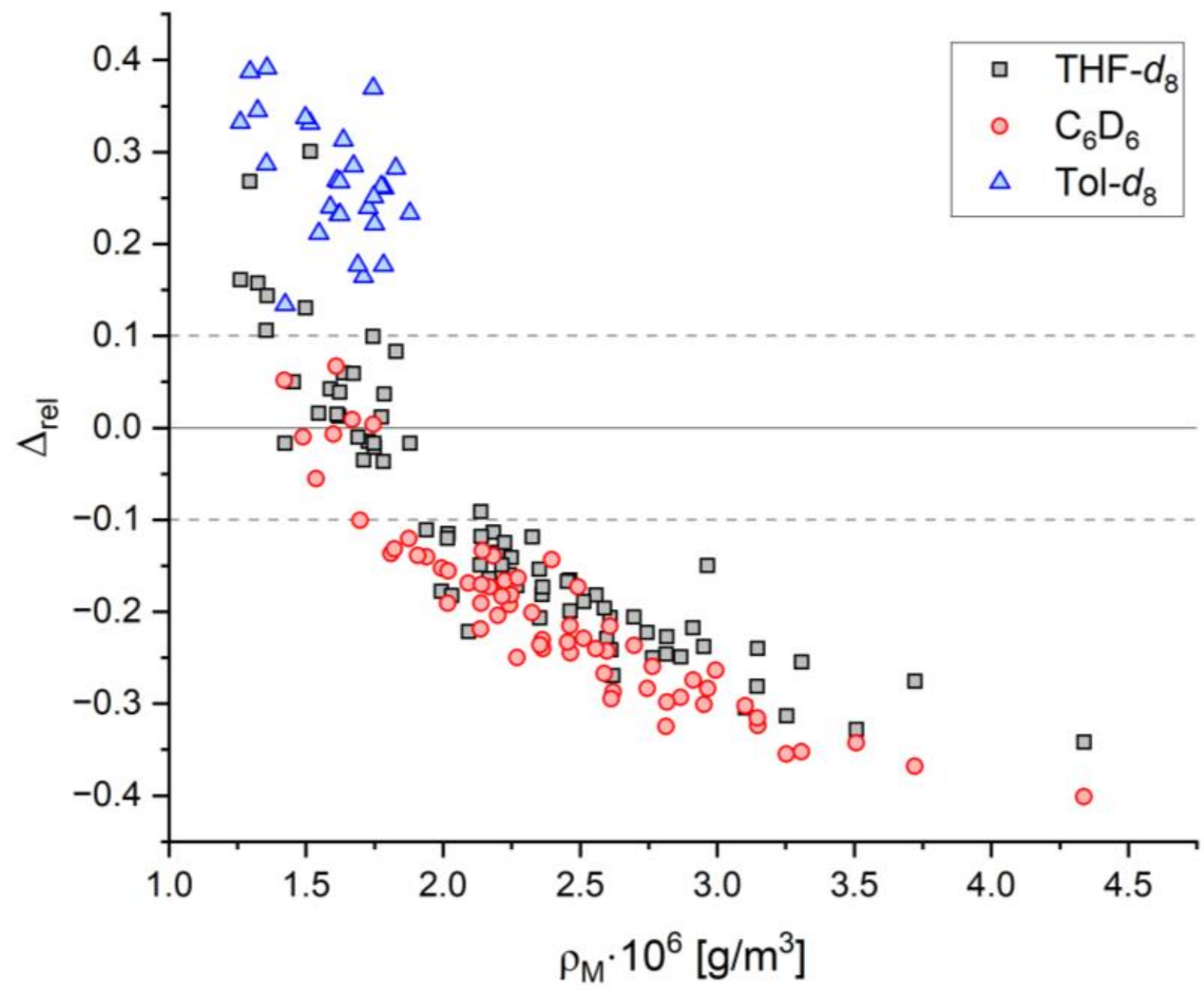

Figure SI-33: Relative errors for our dataset for the model by *Hiller*, *Grabe* and *Schonert* plotted against the molecular density.

16 Semi-empirical Stokes-Einstein Fits

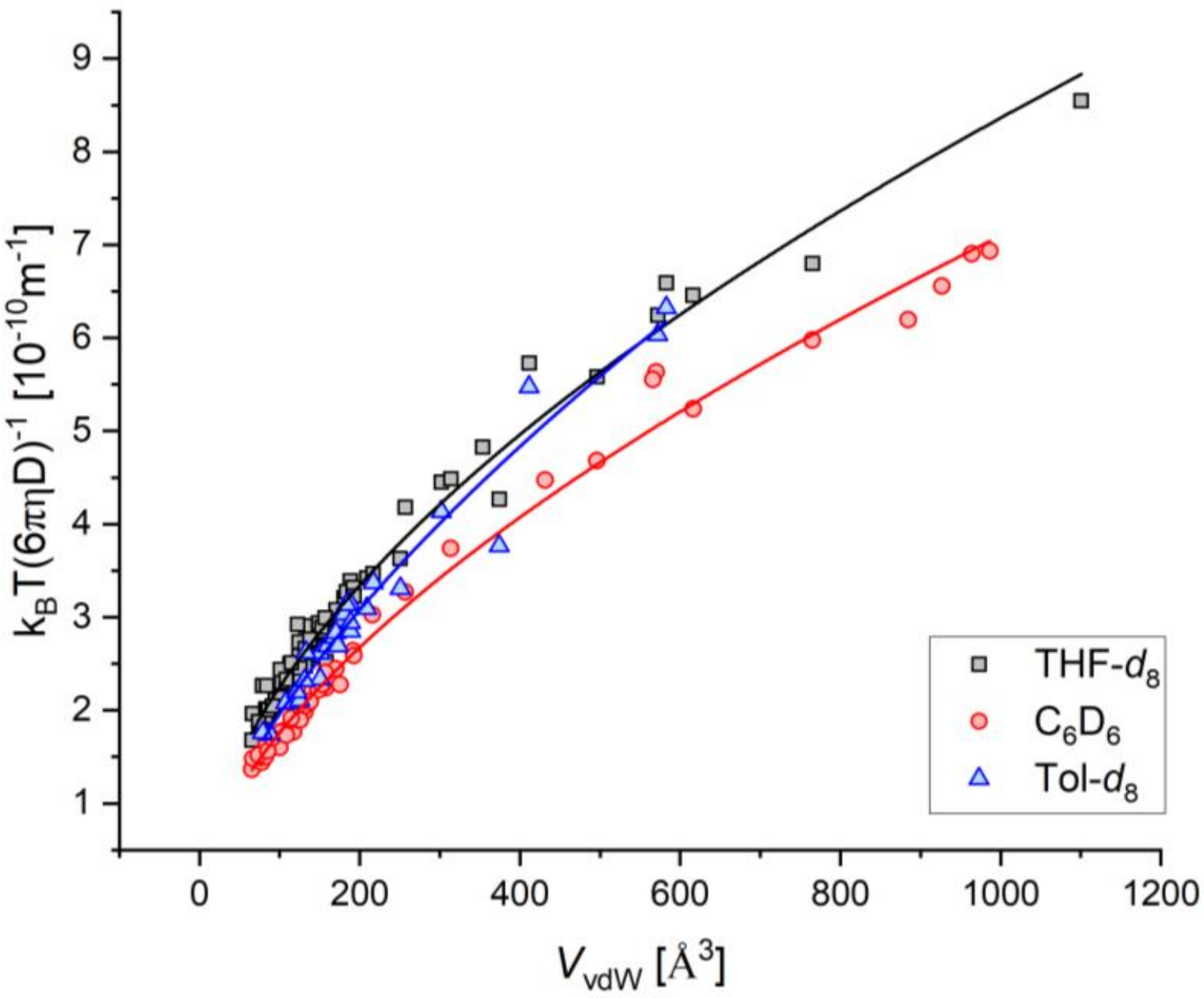

Figure SI-34: Scatter-plots of r_H^{-1} vs. V_{vdW}. The lines represent the resulting fit-functions according to equation (8). The function parameters can be found in table 1 in the main paper.

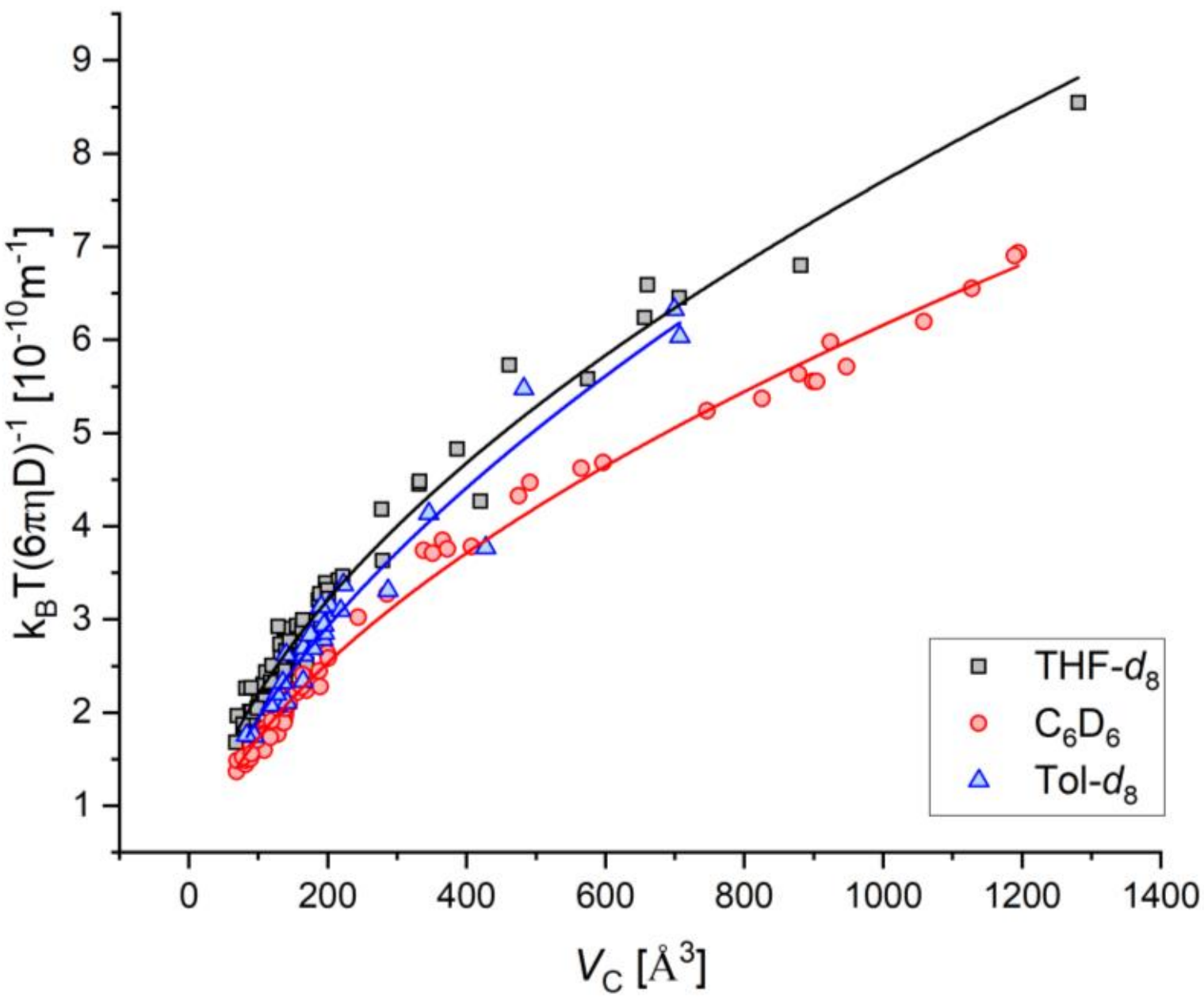

Figure SI-35: Scatter-plots of r_H^{-1} vs. V_C. The lines represent the resulting fit-functions according to equation (8). The function parameters are listed in table SI-19.

Table SI-19: Fitted parameter for the semi-empirical *Stokes-Einstein* model based on V_C, with Å$^{(1-3b)}$ being the unit of a, and b being the fit parameter b.

	a [Å$^{(1-3b)}$]	*b*	R^2	$\bar{\Delta}_{rel}$	2σ
THF-d_8	0.179	0.54	0.978	0.00	0.12
C_6D_6	0.135	0.55	0.992	-0.01	0.10
Tol-d_8	0.126	0.59	0.952	0.01	0.15

17 Step-by-Step Guide

Table SI-20: Step-by-Step Guide for the analysis of DOSY diffusion constants.

Step	Comment
**1**. DOSY Measurement	Follow the common procedures for NMR sample preparation (concentration, homogeneity, …). Deviations caused by differing pulse sequences can be minimized by the *Stalke* normalization.[15]
**2**. DOSY Analysis*	TopSpin in combination with Bruker's Dynamic centre are beginner friendly but powerful tools, that can be used in this endeavour.
**3**. *Stalke* Normalization*	Normalize diffusion constants *D* from signals which can be clearly assigned to only one species. Follow equation 2 in the main paper.[15]
**4**. Determine experimental V_{vdW}*	Use equation 6 and 7 and table 1 from the main paper to determine V_{vdW} of the species in question.
**5**. Model Generation	Use a software such as Avogadro (force field optimization) to generate a simple structural model that could fit the species in question. Save the results as an .xyz file.<br>Crystal structures can also be used, simply convert them to an .xyz file.
**6**. Determine theoretical V_{vdW}	Use software like molovol to determine the V_{vdW} of your model. Simple upload the .xyz into the software and follow their guidelines.

** Step 2, 3 and 4 can be done using the attached excel document.*

18 Computational Methods

DFT Methods: The geometries of [UO_2Cl_2(pynhfu)], [UO_2Cl_2(pynhfu)(thf)], [UO_2Cl_2(pynhfu)(thf)$_2$], [UO_2Cl_2(pynhfu)(thf)](thf), [$UO_2(NO_3)_2$(pynhfu)], [$UO_2(NO_3)_2$(pynhfu)(thf)], [$UO_2(NO_3)_2$(pynhfu)(thf)$_2$] and [$UO_2(NO_3)_2$(pynhfu)(thf)](thf) were optimized and minima were confirmed with numerical ([$UO_2(NO_3)_2$(pynhfu)(thf)$_2$] and [$UO_2(NO_3)_2$(pynhfu)(thf)](thf)) or analytical (other uranyl complexes) frequency calculations using Orca 5.0.4.[70–72] In the case of [$UO_2(NO_3)_2$(pynhfu)(thf)](thf), a small imaginary mode of -17 cm^{-1} remained despite various attempts at removing it. The PBE functional and ZORA-def2-TZVP basis set were employed for most structures.[73,74] All attempts at reaching a minimum structure for [$UO_2(NO_3)_2$(pynhfu)(thf)$_2$], [$UO_2(NO_3)_2$(pynhfu)(thf)](thf), [UO_2Cl_2(pynhfu)(thf)$_2$] and [UO_2Cl_2(pynhfu)(thf)](thf) using PBE failed and thus B3LYP with the ZORA-def2-SVP for all atoms except uranium was used for the geometry optimizations and frequency calculations due to limited memory capacity.[75–78] For uranium, the SARC-ZORA-TZVP basis set was used in all cases.[79] Scalar relativistic effects were taken into account by using the zero order relativistic approximation (ZORA).[80–82] Dispersion corrections were included using Grimme's D3 dispersion correction with Becke-Johnson damping.[83,84]

Force Field Methods: The structures of organic molecules, for which no crystal structure data was available, were obtained using universal force field calculations as implemented in *Avogadro 1.2.0*. [85] For the optimization of [UO_2(acac)$_2$(thf)] and [UO_2(acac)$_2$(thf)$_2$] the crystal structure of *Taylor* and *McLaren* was used as a starting point and one or two THF molecules were additionally included in the coordination sphere of uranium.[27] [UO_2(salophen)(thf)] and [UO_2(salophen)(thf)$_2$] were generated using the crystal structure of *Takao* and *Ikeda* with a THF molecule added at the uranium centre.[86] [UO_2(salen)(thf)] and [UO_2(salen)(thf)$_2$] were obtained by adding one or two THF molecules to the coordination sphere based on the crystal structure of *Hardwick et al.*.[87] The structures were then optimized using the force field simulation of Avogadro while constraining everything but the THF.[85]

18.1 Coordinates of Optimized Structures

18.1.1 Organic Molecules

Benzene

C	-3.77492	2.53515	0.00000
C	-4.01974	1.15769	0.00000
C	-2.94924	0.25694	-0.00000
C	-1.63392	0.73365	0.00000
C	-1.38910	2.11110	0.00000
C	-2.45960	3.01185	0.00000
H	-2.27019	4.07749	0.00000
H	-4.60309	3.23199	0.00000
H	-0.80575	0.03680	0.00000
H	-0.37153	2.47989	0.00000
H	-5.03731	0.78890	-0.00000
H	-3.13865	-0.80870	-0.00000

Cyclopentane

C	-7.96973	4.52801	0.09747
C	-8.32870	3.10694	-0.30799
C	-7.18357	2.32568	0.32663
H	-8.27535	3.00917	-1.41661
H	-9.33897	2.79230	0.03072
C	-5.99117	3.13246	-0.16052
H	-7.14538	1.25698	0.02705
H	-7.25285	2.38930	1.43693
C	-6.43799	4.59410	-0.02178

H	-5.90675	2.91274	-1.25073
H	-5.00856	2.89745	0.30016
H	-6.08467	5.15850	-0.91188
H	-6.04330	5.07431	0.90051
H	-8.21608	4.59506	1.18282
H	-8.52000	5.34360	-0.41696

Tetrahydrofuran

C	-6.21026	3.84263	-0.15843
C	-6.75916	2.51488	0.24970
C	-5.53719	1.66037	-0.03366
H	-7.68140	2.19484	-0.28002
H	-6.95380	2.52675	1.34753
O	-4.40206	2.41297	0.35530
H	-5.45716	1.46902	-1.12772
H	-5.60038	0.69063	0.50362
C	-4.85032	3.74777	0.50882
H	-4.14207	4.48032	0.06743
H	-4.95157	3.94694	1.59971
H	-6.81454	4.72397	0.14431
H	-6.07746	3.85348	-1.26546

Tetramethylsilane

C	-5.14079	4.94993	1.48156
Si	-6.82030	4.18080	1.75178
H	-4.62944	5.07461	2.45727
H	-5.26241	5.94096	0.99751
H	-4.53419	4.29080	0.82672
C	-6.61759	2.51241	2.56463
H	-6.01049	1.85411	1.90941
H	-7.61629	2.05579	2.72380
H	-6.10698	2.63588	3.54087
C	-7.84303	5.28931	2.85203
H	-8.84131	4.83174	3.01111
H	-7.96372	6.28022	2.36750
H	-7.33303	5.41415	3.82842
C	-7.67973	3.97156	0.10899
H	-7.07182	3.31281	-0.54468
H	-7.80009	4.96310	-0.37388
H	-8.67775	3.51450	0.26978

Methyl tert-butyl ether

C	-6.59493	4.01564	1.76901
O	-7.78164	3.24786	1.81143
H	-6.19616	4.08894	2.80138
H	-6.80859	5.05108	1.42898
H	-5.79275	3.54590	1.16518
C	-8.22858	2.77442	0.54051
C	-8.23496	3.86784	-0.55864
H	-8.84498	4.73861	-0.23557
H	-8.66473	3.46893	-1.50300
H	-7.20835	4.21987	-0.79236
C	-9.68083	2.30905	0.74145
H	-10.31583	3.15695	1.07807

H	-9.73019	1.51466	1.51739
H	-10.10713	1.90349	-0.20203
C	-7.39372	1.55838	0.08534
H	-6.33701	1.83055	-0.10797
H	-7.80256	1.13571	-0.85799
H	-7.41233	0.76464	0.86273

2,2,3,3-Tetramethylbutane

C	-1.30717	-1.66829	-0.89919
C	-1.30747	-2.33092	-2.36649
C	-0.58163	-1.43541	-3.40619
H	-1.07417	-0.44930	-3.52016
H	-0.59488	-1.91331	-4.41016
H	0.48404	-1.27810	-3.14619
C	-0.59317	-3.70909	-2.37939
H	0.47212	-3.62704	-2.08541
H	-0.60620	-4.14617	-3.40180
H	-1.09443	-4.44243	-1.71686
C	-2.74795	-2.56298	-2.89653
H	-3.31218	-1.61419	-2.99364
H	-3.32053	-3.25838	-2.25112
H	-2.72229	-3.02064	-3.90965
C	-2.02149	-0.29013	-0.88629
H	-1.52024	0.44322	-1.54883
H	-2.00845	0.14695	0.13612
H	-3.08678	-0.37219	-1.18026
C	0.13330	-1.43622	-0.36916
H	0.70587	-0.74081	-1.01457
H	0.69755	-2.38501	-0.27206
H	0.10765	-0.97857	0.64396
C	-2.03300	-2.56381	0.14050
H	-3.09867	-2.72113	-0.11949
H	-2.01976	-2.08591	1.14447
H	-1.54045	-3.54992	0.25448

Adamantane

C	-4.69869	5.92179	-7.13959
C	-3.66860	4.92895	-6.55481
C	-3.96142	7.02515	-7.93195
H	-5.29320	6.37851	-6.31799
H	-5.40603	5.38367	-7.80828
C	-3.15562	6.38063	-9.08264
C	-2.99706	7.77881	-6.98813
H	-4.70288	7.73884	-8.35224
C	-1.96573	6.78733	-6.40324
H	-3.57069	8.25832	-6.16468
H	-2.47276	8.58480	-7.54721
C	-2.12441	5.38813	-8.49927
H	-2.63327	7.16946	-9.66742
H	-3.84403	5.84814	-9.77519
C	-2.86302	4.28596	-7.70651
H	-3.54783	3.72777	-8.38217
H	-2.12888	3.55866	-7.29524
C	-2.70445	5.68414	-5.61200

H	-4.19954	4.13569	-5.98508
H	-1.96836	4.97401	-5.17502
H	-3.27448	6.13794	-4.77164
C	-1.16139	6.14298	-7.55504
H	-1.27249	7.33005	-5.72455
H	-0.40636	5.43848	-7.14191
H	-0.61455	6.92889	-8.12107
H	-1.54525	4.92497	-9.32741

Si(SiMe$_3$)$_4$

Si	0.86470	8.57858	-3.74669
Si	0.04642	10.30046	-2.54398
Si	-0.45545	12.01002	-3.92475
Si	-1.81519	9.64063	-1.45769
C	0.88219	7.01272	-2.72991
H	-0.15376	6.74931	-2.43948
H	1.49917	7.15115	-1.82135
H	1.30587	6.18793	-3.33844
C	-0.18088	8.27755	-5.26400
H	-1.22606	8.05761	-4.97324
H	0.23236	7.41528	-5.82597
H	-0.15635	9.17456	-5.91339
C	2.61152	8.93304	-4.30259
H	3.26376	9.05526	-3.41570
H	2.64053	9.85455	-4.91519
H	2.98187	8.07969	-4.90653
C	-0.71601	13.58534	-2.95702
H	-1.53418	13.45476	-2.22294
H	-0.97937	14.40389	-3.65753
H	0.21953	13.85268	-2.42766
C	0.92573	12.29798	-5.14765
H	0.66131	13.15445	-5.80082
H	1.05762	11.39488	-5.77530
H	1.87010	12.52186	-4.61533
C	-2.01691	11.64833	-4.88242
H	-2.86404	11.53369	-4.17803
H	-1.89828	10.72107	-5.47522
H	-2.22958	12.49553	-5.56587
C	-1.38085	8.54632	-0.00859
H	-0.82246	7.65500	-0.35367
H	-2.31399	8.22173	0.49536
H	-0.76275	9.11632	0.71251
C	-2.76058	11.11571	-0.81237
H	-3.08174	11.74925	-1.66232
H	-2.12622	11.70855	-0.12575
H	-3.65957	10.76225	-0.26742
C	-2.93929	8.68030	-2.59796
H	-2.41907	7.76640	-2.94594
H	-3.22388	9.30067	-3.46943
H	-3.85370	8.38485	-2.04419
Si	1.59161	10.97262	-1.04680
C	2.95878	11.92347	-1.89106
C	2.35161	9.50477	-0.17855
C	0.82167	12.08003	0.24429

H	2.86767	8.86363	-0.91987
H	1.56961	8.91769	0.34023
H	3.09293	9.86437	0.56389
H	3.44455	11.29526	-2.66222
H	3.71286	12.22517	-1.13579
H	2.53897	12.83346	-2.36289
H	0.36410	12.96742	-0.23371
H	1.60599	12.41041	0.95547
H	0.04715	11.51613	0.80024

Toluene

C	1.86011	7.18295	3.94305
C	1.41065	6.04565	3.26612
C	0.21659	6.09004	2.54101
C	-0.52986	7.27172	2.49167
C	-0.08470	8.41791	3.16904
C	1.11652	8.36671	3.89621
C	-0.89410	9.67936	3.10857
H	-1.90592	9.49384	3.52655
H	-0.99192	10.00967	2.05301
H	-0.41874	10.49789	3.68996
H	2.78495	7.14734	4.50424
H	1.47804	9.23930	4.42592
H	1.98733	5.13049	3.30346
H	-0.13068	5.20887	2.01714
H	-1.45365	7.29436	1.92659

Diisopropyl ether

C	3.12721	4.23447	4.28746
C	2.36293	5.08106	3.27090
C	1.65933	4.18392	2.24848
H	3.64226	4.89695	5.01533
H	3.89193	3.61143	3.77600
H	2.43117	3.56939	4.84249
H	2.40173	3.55477	1.71262
H	1.12296	4.80377	1.49995
H	0.92284	3.52330	2.75414
O	1.40348	5.85870	3.96788
H	3.12510	5.70799	2.74903
C	1.20441	7.12686	3.36522
C	2.12283	8.16228	4.02072
H	1.92504	8.22007	5.11254
H	1.95793	9.16396	3.56954
H	3.18610	7.88225	3.86827
C	-0.26032	7.52175	3.54646
H	1.39111	7.11214	2.26466
H	-0.91472	6.76961	3.05656
H	-0.45466	8.51273	3.08306
H	-0.52015	7.57010	4.62582

1,3-Indandione

C	1.72290	-0.06340	0.02688
C	0.36620	0.21221	0.29361
C	0.00916	1.05054	1.36352

C	1.02967	1.59518	2.14454
C	2.35188	1.32658	1.88458
C	2.73205	0.49739	0.82819
C	3.17840	2.03061	2.86038
C	2.31906	2.80884	3.81741
C	0.94567	2.48418	3.29935
H	-1.03009	1.26677	1.57452
H	1.98880	-0.71010	-0.79912
H	-0.40441	-0.22393	-0.32861
H	3.77566	0.29049	0.62968
H	2.52693	3.89752	3.74977
H	2.45079	2.44873	4.85952
O	-0.09450	2.90623	3.77897
O	4.39802	1.99359	2.89572

2-Phenylpyridine

C	1.47234	0.22802	0.39165
C	0.18871	0.60046	0.78760
C	0.01028	1.45105	1.88226
C	1.11791	1.94735	2.60476
C	2.41105	1.55709	2.18627
C	2.58075	0.70559	1.08983
C	0.88719	2.85690	3.77252
H	-1.00176	1.71733	2.15883
H	1.60834	-0.43077	-0.45632
H	-0.67169	0.22961	0.24547
H	3.57683	0.41527	0.78087
H	3.30384	1.89444	2.68705
C	1.93779	3.39594	4.54674
C	1.64439	4.23791	5.62222
C	0.32028	4.54410	5.92909
C	-0.69915	4.00030	5.15084
N	-0.39551	3.18312	4.10835
H	2.97600	3.18666	4.34621
H	2.44636	4.65364	6.21874
H	0.08673	5.19579	6.76106
H	-1.73315	4.22671	5.37396

1,1,3,3-Tetramethoxypropane

O	9.67244	3.90176	4.48995
C	8.50924	3.12593	4.24524
O	8.52156	2.69810	2.89694
C	10.24179	3.57113	5.73691
H	10.62501	2.52690	5.73423
H	9.52220	3.74014	6.56841
H	11.10828	4.24039	5.91468
C	9.39998	1.60551	2.74345
H	10.43778	1.86754	3.04663
H	9.43702	1.33722	1.66781
H	9.02773	0.71150	3.29058
C	7.27339	4.01138	4.48571
H	8.40162	2.24415	4.93150
C	5.94054	3.26845	4.28391
H	7.31227	4.89505	3.81117

H	7.30955	4.37835	5.53459
O	4.87816	4.16777	4.56213
C	4.31341	3.90006	5.82616
H	3.53199	4.66075	6.02874
H	5.07190	3.98705	6.63541
H	3.81713	2.90463	5.83671
O	5.84051	2.84686	2.93730
H	5.97052	2.37904	4.96810
C	4.84242	1.85839	2.81206
H	5.12975	0.92779	3.34927
H	4.74368	1.59764	1.73846
H	3.84955	2.23311	3.14581

Bromoethane

C	-7.18412	2.44137	0.01592
C	-5.66541	2.37054	0.03685
H	-7.53927	3.21211	0.73220
H	-7.60850	1.45740	0.30672
H	-7.53927	2.69891	-1.00426
H	-5.31637	2.10697	1.05747
Br	-4.90678	4.08006	-0.46838
H	-5.31637	1.59451	-0.67652

Allylbromide

C	-7.07632	2.43192	0.01871
C	-5.57778	2.44675	0.01433
C	-7.73715	1.31961	0.34745
H	-5.20054	2.20376	1.03036
Br	-4.89922	4.18744	-0.50011
H	-5.20054	1.69048	-0.70638
H	-7.63522	3.32552	-0.24538
H	-7.19790	0.41596	0.61451
H	-8.82100	1.30693	0.35119

2-Bromopropene

C	-7.00047	2.41033	0.02509
C	-5.50188	2.49038	0.00143
C	-7.63828	1.28187	0.35860
H	-5.08802	2.27811	1.00977
H	-5.18109	3.51004	-0.29992
H	-5.08802	1.76408	-0.72952
Br	-8.02678	3.95962	-0.43278
H	-7.09764	0.37874	0.62551
H	-8.72350	1.25519	0.36649

2-Bromopropane

C	-7.19050	2.49903	0.26810
C	-5.69708	2.37495	-0.02608
C	-7.87741	1.13633	0.20937
H	-5.21290	1.70804	0.71885
H	-5.21706	3.37459	0.03338
H	-5.53357	1.95922	-1.04337
Br	-8.01151	3.69985	-1.01762
H	-7.32435	2.92789	1.28463

H	-8.95838	1.24921	0.43740
H	-7.43052	0.44824	0.95833
H	-7.76965	0.68893	-0.80189

1-Bromopropane

C	-7.24290	2.48766	0.20285
C	-5.74996	2.32069	-0.07193
C	-7.96390	1.14352	0.16864
H	-5.28933	1.66351	0.69626
Br	-4.86619	4.04400	-0.02113
H	-5.59222	1.87108	-1.07550
H	-7.68716	3.15664	-0.56563
H	-7.38464	2.94933	1.20397
H	-9.04495	1.29586	0.37130
H	-7.54926	0.46313	0.94279
H	-7.85241	0.67087	-0.83052

2-Bromo-2-butene (Z)

C	-7.92443	2.91784	-0.00353
C	-6.71735	3.51797	0.03139
C	-8.09504	1.51261	0.49702
Br	-9.50284	3.78253	-0.68065
H	-5.86395	2.97349	0.42696
C	-6.44886	4.91785	-0.44565
H	-7.34894	5.42885	-0.83863
H	-5.68877	4.88572	-1.25344
H	-6.05099	5.51814	0.39853
H	-7.14289	1.07928	0.87168
H	-8.47131	0.87374	-0.32873
H	-8.83393	1.50685	1.32504

2-Bromo-2-butene (E)

C	-8.16871	3.01066	-0.29802
C	-6.97439	3.57726	-0.04260
Br	-9.55885	4.10546	-1.03509
C	-5.78617	2.85602	0.53363
C	-8.46814	1.56162	-0.03442
H	-7.78738	0.91777	-0.63018
H	-9.51337	1.32510	-0.32297
H	-8.34527	1.33801	1.04623
H	-5.97914	1.78410	0.73812
H	-5.49375	3.34391	1.48642
H	-4.93921	2.92621	-0.17988
H	-6.84415	4.63246	-0.26377

2-Bromo-2-methylpropane

C	-7.71729	2.65548	-0.39648
C	-7.53617	4.04001	0.23982
Br	-7.98093	2.87375	-2.31195
C	-8.94929	1.95956	0.19724
H	-9.09514	0.96314	-0.27229
H	-9.86202	2.56674	0.01599
H	-8.83074	1.81919	1.29342
C	-6.46497	1.80007	-0.16287

H	-8.43357	4.66976	0.05904
H	-6.65502	4.55557	-0.19876
H	-5.57220	2.29132	-0.60582
H	-6.58386	0.80192	-0.63631
H	-6.28529	1.65577	0.92445
H	-7.38285	3.95082	1.33705

1-Bromobutane

C	-7.07437	1.86202	-2.36282
C	-5.54864	1.83979	-2.35970
H	-7.45289	2.50346	-1.53857
H	-7.46200	0.83161	-2.21807
H	-7.45289	2.25154	-3.33190
H	-5.19791	1.42601	-1.38901
C	-4.97463	3.24715	-2.55740
H	-5.19791	1.17458	-3.17881
H	-5.32806	3.90992	-1.73729
H	-5.32806	3.65831	-3.52838
C	-3.44745	3.22209	-2.55388
H	-3.07212	2.82878	-1.58508
Br	-2.75036	5.01220	-2.80535
H	-3.07212	2.57708	-3.37682

2-Bromo-2-methylpropane

C	-5.45284	1.72308	-2.40928
H	-5.11669	1.28955	-1.44246
C	-4.91988	3.16003	-2.57768
H	-5.08592	1.06870	-3.22955
C	-5.46042	4.02969	-1.42442
C	-5.41736	3.72060	-3.92557
C	-3.37067	3.12044	-2.54612
H	-3.02096	2.69043	-1.58266
Br	-2.51918	4.86131	-2.74661
H	-2.99030	2.47034	-3.36351
H	-6.52832	3.72256	-3.96196
H	-5.04309	3.10128	-4.76917
H	-5.07250	4.76456	-4.07937
H	-6.57196	4.03580	-1.42728
H	-5.11660	5.08113	-1.51771
H	-5.11757	3.63588	-0.44327
H	-6.56428	1.71014	-2.42682

1-Bromopentane

C	-7.81114	6.19582	-3.65906
C	-6.28606	6.14588	-3.64762
H	-8.21437	5.90893	-2.66447
H	-8.21436	5.50472	-4.42971
H	-8.14811	7.22715	-3.89521
C	-5.78089	4.73505	-3.32457
H	-5.90956	6.86159	-2.88447
H	-5.90956	6.45820	-4.64617
H	-6.16409	4.42769	-2.32635
C	-4.24861	4.68936	-3.31411
H	-6.16409	4.02394	-4.08959

H	-3.86215	5.39733	-2.54852
H	-3.86214	4.99365	-4.31148
C	-3.74329	3.28427	-2.99237
H	-4.10502	2.96593	-1.99141
Br	-1.80607	3.25732	-2.98619
H	-4.10502	2.56208	-3.75507

Bromobenzene

C	-2.05039	3.23376	-2.18810
C	-2.80552	4.05621	-3.03091
C	-4.19464	3.89773	-3.10354
C	-4.82655	2.91534	-2.33175
C	-4.07044	2.09345	-1.48928
C	-2.68266	2.25258	-1.41743
Br	-5.22623	5.02019	-4.25393
H	-5.90024	2.78861	-2.38415
H	-4.55945	1.33413	-0.89283
H	-2.09804	1.61647	-0.76548
H	-0.97650	3.35670	-2.13232
H	-2.31198	4.81419	-3.62548

3-Bromopyridine

C	-2.04813	3.23528	-2.18910
C	-2.79946	4.05788	-3.03146
C	-4.18616	3.89805	-3.10253
C	-4.79998	2.91151	-2.32430
N	-4.04917	2.12115	-1.51052
C	-2.69868	2.26109	-1.42741
Br	-5.22986	5.01348	-4.24856
H	-2.13026	1.61647	-0.77042
H	-0.97384	3.35114	-2.12700
H	-2.30580	4.81581	-3.62596
H	-5.87205	2.77221	-2.36533

Bromocyclohexane

C	-2.01958	3.12488	-2.47091
C	-2.84153	4.37084	-2.84138
C	-4.28265	3.99371	-3.23169
C	-4.94932	3.20313	-2.09067
C	-4.12702	1.95736	-1.72033
C	-2.69893	2.36074	-1.32508
Br	-4.32133	2.98864	-4.90368
H	-2.10786	1.45316	-1.07722
H	-2.73571	3.00823	-0.42116
H	-4.86328	4.92295	-3.41653
H	-5.97970	2.90854	-2.38392
H	-5.02355	3.85809	-1.19435
H	-0.99790	3.43155	-2.16005
H	-1.92170	2.45771	-3.35423
H	-2.35263	4.91793	-3.67572
H	-2.87306	5.04945	-1.96026
H	-4.08894	1.25706	-2.58235
H	-4.61307	1.42875	-0.87249

2-Bromostyrene

C	-1.83308	3.26625	-2.02834
C	-1.89645	3.21341	-3.43988
C	-3.17488	3.15835	-4.05182
C	-4.33362	3.15711	-3.26512
C	-4.24640	3.20978	-1.87523
C	-2.99886	3.26421	-1.25869
Br	-3.42029	3.08295	-5.95605
H	-2.93346	3.30507	-0.17889
H	-0.89453	3.30959	-1.49870
H	-5.30867	3.11505	-3.73377
H	-5.14738	3.20839	-1.27537
C	-0.66158	3.21450	-4.28425
C	0.59382	3.26362	-3.81679
H	-0.76226	3.17288	-5.36248
H	0.83732	3.30795	-2.76438
H	1.42314	3.25952	-4.51719

2-(Bromoethyl)benzene

C	-1.88160	3.28721	-2.07096
C	-1.96449	3.19607	-3.47776
C	-3.23575	3.12636	-4.07490
C	-4.39394	3.14683	-3.29242
C	-4.29827	3.23721	-1.90285
C	-3.04413	3.30725	-1.29355
H	-3.32967	3.05582	-5.15186
H	-2.97193	3.37730	-0.21577
H	-0.93078	3.34339	-1.56054
H	-5.36641	3.09254	-3.76456
H	-5.19580	3.25297	-1.29813
C	-0.75201	3.16885	-4.38843
C	0.60325	3.24772	-3.68097
H	-0.82652	4.02663	-5.09177
H	-0.78129	2.22659	-4.97777
H	0.68498	4.19632	-3.10884
Br	2.03714	3.20128	-4.98351
H	0.73054	2.38412	-2.99406

4-Bromoanisole

C	0.18896	0.04556	-0.04510
C	-0.93419	0.49819	0.66013
C	-0.92053	1.74389	1.29491
H	-1.82232	-0.11875	0.71652
C	0.21903	2.55097	1.23051
C	1.34594	2.11067	0.53005
C	1.33571	0.86520	-0.10606
O	0.12514	-1.17173	-0.64417
C	1.13436	-1.76494	-1.37381
H	1.39289	-1.13689	-2.25290
H	0.78636	-2.75397	-1.73594
H	2.03112	-1.91688	-0.73598
Br	0.23639	4.25235	2.09804
H	2.23033	2.73270	0.47784
H	2.22184	0.55057	-0.64062

H	-1.79555	2.08038	1.83603

2-Bromomesitylene

C	0.17503	0.01420	-0.03803
C	-0.94805	0.46192	0.66384
C	-0.94662	1.71430	1.29704
H	-1.82640	-0.17117	0.71514
C	0.20258	2.53447	1.22580
C	1.34158	2.08251	0.51506
C	1.31286	0.82595	-0.10873
C	0.14653	-1.32948	-0.70598
Br	0.18931	4.25493	2.10212
C	2.60627	2.89189	0.38949
H	2.18473	0.48040	-0.65257
C	-2.17469	2.16221	2.04341
H	2.56071	3.86982	0.90372
H	2.81642	3.08476	-0.68378
H	3.45446	2.32170	0.82390
H	-2.98440	1.40282	2.00174
H	-2.56316	3.10218	1.59816
H	-1.92313	2.33675	3.11063
H	-0.67113	-1.35566	-1.45679
H	-0.03207	-2.11985	0.05323
H	1.10432	-1.55075	-1.22331

1-Bromonaphthalene

C	4.88102	3.16727	0.59992
C	6.05092	2.73174	0.09857
C	6.10843	1.42586	-0.61971
C	4.92312	0.66946	-0.75851
C	3.65768	1.18931	-0.18989
C	3.64101	2.36876	0.45082
C	7.30749	0.93011	-1.16021
C	7.32496	-0.29782	-1.82729
C	6.15078	-1.04189	-1.96196
C	4.95102	-0.56180	-1.42976
Br	7.60902	3.83036	0.33736
H	4.83419	4.11701	1.12344
H	6.17159	-1.99218	-2.47987
H	4.04775	-1.14896	-1.54133
H	8.23174	1.48364	-1.07157
H	8.25243	-0.67264	-2.24070
H	2.73740	0.62056	-0.28765
H	2.71176	2.74606	0.86598

6-Bromoquinoline

N	0.28932	0.14570	-0.03465
C	-0.83672	0.61846	0.56355
C	-0.82576	1.86357	1.19764
H	-1.74232	0.02698	0.54710
C	0.34958	2.61795	1.21220
C	1.50352	2.12060	0.59258
C	1.44951	0.85644	-0.03764
H	0.35664	3.58319	1.70421

C	2.70153	2.85042	0.58740
C	2.58972	0.33977	-0.66281
H	-1.72157	2.24049	1.67395
C	3.78028	1.07336	-0.66371
C	3.83725	2.32558	-0.04053
H	2.55604	-0.62805	-1.14773
H	4.65876	0.66801	-1.14910
H	2.75584	3.82051	1.06695
Br	5.46804	3.31972	-0.04725

1-Bromo-4-butylbenzene

C	0.73806	0.19472	-0.19768
C	2.12140	2.27466	0.45370
C	2.01997	1.00476	-0.15519
C	3.33799	2.96442	0.45438
C	3.16455	0.45325	-0.75740
C	4.37809	1.14676	-0.75398
C	4.46597	2.40233	-0.14843
Br	6.12484	3.34876	-0.14453
H	3.40252	3.93735	0.92462
H	1.27395	2.74542	0.93093
H	5.24916	0.70685	-1.22242
H	3.11621	-0.51937	-1.23209
C	-0.47811	0.84899	0.47655
H	0.93226	-0.78154	0.29854
H	0.48806	0.00338	-1.26442
H	-0.25098	1.02888	1.55055
C	-1.71386	-0.05319	0.37472
H	-0.69799	1.81871	-0.02217
H	-1.50802	-1.02699	0.87041
H	-1.95294	-0.24089	-0.69486
C	-2.92414	0.59267	1.04311
H	-2.72064	0.77148	2.12047
H	-3.80221	-0.08118	0.95429
H	-3.16648	1.55921	0.55202

Dibromomethane

Br	8.43444	5.24655	0.91891
C	10.36781	5.25304	0.93305
H	10.73340	6.29567	1.03328
Br	11.02692	4.49499	-0.71895
H	10.73340	4.64910	1.78884

1,2-Dibromoethane

Br	8.38674	5.25327	0.93643
C	10.32231	5.22093	0.86289
H	10.70176	6.26029	0.95769
C	10.78747	4.61645	-0.45406
H	10.70298	4.61603	1.71284
H	10.40679	5.22134	-1.30401
H	10.40801	3.57709	-0.54886
Br	12.72304	4.58411	-0.52760

1,2-Dibromopropane

Br	10.90567	7.09023	-1.18877
C	10.53754	5.43210	-0.24786
H	10.98963	5.48158	0.76574
C	11.07558	4.21877	-1.01410
H	9.43473	5.35029	-0.14110
H	10.62914	4.20384	-2.03182
C	10.70258	2.92066	-0.30018
Br	13.01199	4.31098	-1.18311
H	9.59839	2.82957	-0.21859
H	11.14305	2.89629	0.71957
H	11.08363	2.05051	-0.87551

1,2-Dibromobenzene

C	13.65422	2.72793	-2.72311
C	13.12621	1.45753	-2.47044
C	11.75811	1.30341	-2.21267
C	10.92260	2.42668	-2.20926
C	11.44801	3.69743	-2.46152
C	12.81998	3.85286	-2.72012
Br	13.58212	5.57604	-3.07085
Br	10.26553	5.20121	-2.44581
H	14.71139	2.84931	-2.92258
H	13.77923	0.59252	-2.47474
H	11.34074	0.32301	-2.01596
H	9.86463	2.31187	-2.01048

1,4-Dibromobenzene

C	13.64252	2.84085	-2.73535
C	13.10862	1.57334	-2.48203
C	11.74295	1.42343	-2.22522
C	10.90664	2.54334	-2.22124
C	11.43154	3.81514	-2.47354
C	12.80426	3.96818	-2.73197
Br	13.52940	5.70024	-3.07738
H	10.76970	4.67606	-2.46719
H	14.70533	2.94226	-2.93328
H	13.75579	0.70945	-2.48545
Br	11.02430	-0.30127	-1.88188
H	9.85172	2.42877	-2.02302

1,7-Dibromoheptane

C	14.29752	-4.22475	-1.78617
H	15.40817	-4.24839	-1.78148
H	13.93087	-4.61015	-2.76144
C	13.79882	-2.79871	-1.56080
Br	13.64912	-5.38819	-0.37923
C	14.30008	-1.85890	-2.66339
H	14.16349	-2.43932	-0.57361
H	12.68678	-2.80093	-1.55319
H	15.41258	-1.86142	-2.66977
C	13.79885	-0.42777	-2.43608
H	13.93571	-2.22307	-3.64945
H	14.16318	-0.06374	-1.44997
H	12.68637	-0.42538	-2.42962

C	14.30009	0.51239	-3.53876
H	15.41259	0.51015	-3.54524
C	13.79884	1.94320	-3.31130
H	13.93572	0.14850	-4.52493
H	14.16350	2.31145	-2.32738
H	12.68680	1.94984	-3.30696
C	14.29755	2.88074	-4.40920
H	15.40819	2.90176	-4.42099
Br	13.64915	4.67930	-4.09569
H	13.93090	2.54000	-5.40096

Iodoethane

C	6.30585	7.72110	-0.13206
H	7.41613	7.71461	-0.14577
C	5.81128	8.02350	1.27329
I	5.59231	5.81303	-0.77697
H	5.93968	8.49944	-0.83424
H	6.17949	7.25055	1.98037
H	4.70109	8.03641	1.29099
H	6.18809	9.01646	1.59712

Allyliodide

C	6.29397	7.63092	-0.20938
H	7.40324	7.59798	-0.24787
C	5.84970	8.00659	1.17161
I	5.53765	5.71356	-0.77313
H	5.92479	8.38387	-0.93727
C	6.21975	9.17263	1.70725
H	5.22263	7.33263	1.74811
H	6.84638	9.86327	1.15073
H	5.89718	9.44278	2.70699

2-Iodopropane

C	6.00891	7.57270	-0.59862
C	5.90405	7.73198	0.91633
I	5.25184	5.65409	-1.17607
H	6.50478	6.95263	1.43203
H	6.27160	8.73421	1.22378
H	4.84405	7.63438	1.23233
C	7.45465	7.71737	-1.06802
H	8.09472	6.93764	-0.60267
H	7.84900	8.71934	-0.79486
H	7.50426	7.60932	-2.17204
H	5.38922	8.35441	-1.08861

1-Iodobutane

C	9.16914	6.58436	-5.95731
C	9.39494	6.92366	-4.48567
H	8.57386	5.65098	-6.04987
H	10.14288	6.44703	-6.47413
I	8.10554	8.16487	-6.92520
C	10.15716	5.80137	-3.77258
H	8.40910	7.07312	-3.99346
H	9.97705	7.86863	-4.41744

H	9.57707	4.85501	-3.83854
C	10.38394	6.13829	-2.30174
H	11.14396	5.64998	-4.26223
H	9.41137	6.27353	-1.78220
H	10.98133	7.07006	-2.20672
H	10.93695	5.30984	-1.81101

2,2'-Dimethyl-1-iodopropane

C	9.19823	6.49550	-6.03610
C	9.44292	6.87418	-4.55332
H	8.63789	5.53740	-6.09537
H	10.16999	6.35015	-6.55575
I	8.08822	7.93630	-7.18656
C	10.24548	5.74145	-3.88226
C	8.10179	7.04054	-3.81084
C	10.25379	8.18218	-4.45751
H	9.68596	4.78201	-3.92839
H	11.22364	5.59774	-4.39046
H	10.44178	5.97673	-2.81367
H	11.22810	8.07819	-4.98210
H	10.45670	8.43923	-3.39529
H	9.70257	9.03225	-4.91061
H	7.50614	6.10368	-3.86367
H	7.49823	7.86284	-4.24823
H	8.27542	7.28206	-2.73983

1-Iodopentane

C	7.91317	5.57908	-8.88490
C	6.72164	5.49347	-7.93549
H	7.85872	4.77229	-9.64663
H	8.86484	5.48403	-8.31975
H	7.90506	6.56011	-9.40497
C	6.69773	4.15038	-7.19692
H	5.78544	5.61350	-8.52330
H	6.78952	6.32382	-7.19908
C	5.49931	4.06886	-6.24455
H	7.63833	4.03529	-6.61399
H	6.63339	3.32433	-7.93940
C	5.47336	2.73163	-5.50756
H	4.55696	4.18063	-6.82414
H	5.56168	4.89146	-5.49898
H	5.39390	1.89407	-6.23303
I	3.79398	2.64726	-4.18906
H	6.39932	2.60542	-4.90691

Iodobenzene

C	7.56910	6.92120	-9.67239
C	8.62271	6.07942	-10.04354
C	8.55093	4.70877	-9.77412
C	7.42565	4.17940	-9.13350
C	6.37065	5.02107	-8.76165
C	6.44342	6.39273	-9.03174
I	4.67382	4.22364	-7.79579
H	7.37407	3.11827	-8.92687

H	7.62482	7.98179	-9.88096
H	5.63035	7.04752	-8.74621
H	9.49351	6.48866	-10.53921
H	9.36631	4.05755	-10.06139

2-Iodotoluene

C	-2.94574	3.39715	0.00000
C	-3.29024	2.04082	0.00000
C	-2.29161	1.06695	-0.00000
C	-0.94917	1.44395	-0.00000
C	-0.58795	2.79587	0.00000
C	-1.59380	3.79179	0.00000
C	-1.26899	5.26313	0.00000
H	-0.18149	5.46255	0.00000
H	-1.69607	5.74509	0.90435
H	-1.69607	5.74509	-0.90435
I	1.49509	3.21069	-0.00000
H	-3.73688	4.13816	0.00000
H	-4.33098	1.74167	0.00000
H	-2.55511	0.01697	-0.00000
H	-0.18224	0.68102	-0.00000

6-Iodoquinoline

C	7.57827	7.10603	-9.64592
C	8.65725	6.26433	-10.00028
C	8.57630	4.88675	-9.74947
C	7.43089	4.35189	-9.15100
C	6.36306	5.18625	-8.80052
C	6.43624	6.56180	-9.04752
I	4.64070	4.36888	-7.89858
H	5.60767	7.20356	-8.77469
H	9.39411	4.22748	-10.01473
H	7.37339	3.28820	-8.95959
C	9.79029	6.83124	-10.59818
C	9.83572	8.20731	-10.83278
C	8.74761	9.00419	-10.46735
N	7.65299	8.44290	-9.88760
H	10.63461	6.21448	-10.88199
H	10.70725	8.65331	-11.29424
H	8.77364	10.07094	-10.64508

18.1.2 Uranyl Coordination Compounds

[UO_2Cl_2(pynhfu)]

C	-3.786484000	-6.464022000	7.607525000
C	-4.536587000	-7.597011000	7.298724000
C	-5.172749000	-7.664713000	6.061015000
C	-5.023323000	-6.604947000	5.169960000
N	-4.287222000	-5.516768000	5.459313000
C	-3.684475000	-5.442047000	6.662535000
C	-2.896993000	-4.189282000	6.921197000
N	-3.612766000	-3.027990000	6.369075000

C	-2.877171000	-1.763472000	6.588064000
C	-3.608376000	-0.643493000	5.955240000
C	-4.049105000	0.589801000	6.335538000
C	-4.685668000	1.171824000	5.188029000
C	-4.604009000	0.257418000	4.182882000
O	-3.942420000	-0.861923000	4.634049000
H	-2.690976000	-4.081914000	8.001599000
H	-1.928818000	-4.247762000	6.395443000
H	-1.884620000	-1.895592000	6.123985000
H	-2.723165000	-1.545091000	7.659447000
H	-3.926233000	1.037765000	7.316753000
H	-5.147389000	2.151463000	5.122396000
H	-4.925512000	0.207004000	3.148908000
H	-3.285810000	-6.364913000	8.571389000
H	-5.778048000	-8.525542000	5.778388000
H	-5.480700000	-6.614576000	4.180191000
H	-4.629753000	-8.410581000	8.019658000
U	-4.019732000	-3.503641000	3.719271000
O	-5.788216000	-3.323815000	3.973199000
O	-2.229546000	-3.617893000	3.865609000
Cl	-3.968824000	-1.928832000	1.649467000
Cl	-4.203100000	-5.559361000	2.148770000
H	-4.539170000	-2.955924000	6.803834000

[UO_2Cl_2(pynhfu)(thf)]

C	-4.048563000	-6.142709000	7.692023000
C	-4.739922000	-7.298779000	7.336940000
C	-5.193714000	-7.434853000	6.025863000
C	-4.920333000	-6.420769000	5.111769000
N	-4.238394000	-5.312336000	5.448448000
C	-3.819645000	-5.167668000	6.718876000
C	-3.109732000	-3.882098000	7.019323000
N	-3.802777000	-2.767391000	6.342256000
C	-3.238305000	-1.454801000	6.745408000
C	-3.310597000	-1.172327000	8.203776000
C	-2.382235000	-1.054155000	9.201688000
C	-3.104039000	-0.797546000	10.412385000
C	-4.424689000	-0.784615000	10.072283000
O	-4.573986000	-1.008851000	8.729321000
H	-3.039473000	-3.729157000	8.109726000
H	-2.083434000	-3.915809000	6.615018000
H	-3.777752000	-0.690375000	6.170949000
H	-2.186858000	-1.434588000	6.426633000
H	-1.306251000	-1.138959000	9.082627000
H	-2.693141000	-0.642886000	11.404838000
H	-5.342658000	-0.633044000	10.628882000
H	-3.692651000	-5.985912000	8.710913000
H	-5.748899000	-8.315843000	5.704816000
H	-5.227685000	-6.487497000	4.068264000
H	-4.931261000	-8.077569000	8.076795000
U	-3.721695000	-3.387650000	3.634063000
O	-5.518497000	-3.446905000	3.534221000
O	-1.988161000	-3.163639000	4.074739000
Cl	-3.489170000	-2.346302000	1.233633000

Cl	-3.272402000	-5.660686000	2.436344000
H	-4.791293000	-2.787054000	6.617034000
O	-4.044109000	-0.831820000	3.907555000
C	-5.278367000	-0.232679000	3.383631000
H	-5.862571000	0.122154000	4.249443000
H	-5.833868000	-1.018063000	2.859486000
C	-4.827008000	0.910129000	2.488016000
H	-4.618766000	0.528036000	1.479068000
H	-5.581344000	1.704996000	2.421780000
C	-3.527089000	1.363200000	3.162545000
H	-3.739228000	1.990915000	4.042067000
H	-2.866906000	1.925316000	2.489341000
C	-2.909129000	0.036498000	3.575677000
H	-2.267539000	0.091278000	4.464836000
H	-2.358967000	-0.434270000	2.748542000

[UO_2Cl_2(pynhfu)(thf)$_2$]

C	-4.599690000	-6.045605000	5.897276000
C	-5.831654000	-6.568815000	5.506192000
C	-6.968988000	-5.766053000	5.614347000
C	-6.815337000	-4.467770000	6.107224000
N	-5.640498000	-3.963837000	6.489432000
C	-4.551143000	-4.736833000	6.390918000
C	-3.250965000	-4.106761000	6.819822000
N	-3.048191000	-2.821471000	6.129492000
C	-1.878046000	-2.080626000	6.659336000
C	-2.092716000	-1.536459000	8.028598000
C	-1.601503000	-1.826261000	9.272245000
C	-2.249677000	-0.928973000	10.189714000
C	-3.091759000	-0.163530000	9.435672000
O	-3.004528000	-0.521012000	8.126734000
H	-3.271123000	-3.952508000	7.911725000
H	-2.410068000	-4.760475000	6.572338000
H	-1.648891000	-1.263788000	5.967971000
H	-1.015227000	-2.753964000	6.644393000
H	-0.863707000	-2.586639000	9.502944000
H	-2.105570000	-0.865689000	11.262339000
H	-3.781182000	0.637701000	9.670509000
H	-3.680177000	-6.615891000	5.786948000
H	-7.950362000	-6.128391000	5.314268000
H	-7.681494000	-3.807124000	6.190455000
H	-5.900982000	-7.580020000	5.107964000
U	-3.114961000	-2.776465000	3.461018000
O	-4.406240000	-3.996357000	3.373063000
O	-1.863064000	-1.517916000	3.607119000
Cl	-4.371261000	-1.325354000	1.547045000
Cl	-1.343490000	-4.677465000	4.158568000
H	-3.889348000	-2.260998000	6.276715000
O	-4.675106000	-1.122539000	4.620799000
C	-6.117569000	-1.253938000	4.478246000
H	-6.506939000	-1.626129000	5.433370000
H	-6.313644000	-1.985762000	3.690729000
C	-6.603498000	0.150298000	4.145963000
H	-6.509920000	0.322724000	3.067019000

H	-7.645770000	0.311473000	4.443621000
C	-5.606958000	1.028295000	4.915345000
H	-5.865406000	1.062605000	5.982465000
H	-5.555138000	2.056857000	4.541467000
C	-4.295893000	0.278827000	4.705474000
H	-3.586539000	0.383732000	5.532509000
H	-3.813942000	0.553540000	3.760546000
H	-0.500913000	-2.786130000	0.595295000
O	-2.250343000	-3.619397000	1.324906000
C	-2.574459000	-4.948550000	0.845437000
C	-1.827114000	-5.075226000	-0.479571000
C	-1.784854000	-3.622517000	-0.975720000
C	-1.561618000	-2.843362000	0.316073000
H	-2.260095000	-5.661318000	1.613983000
H	-3.663341000	-5.002833000	0.720737000
H	-2.332631000	-5.755747000	-1.173845000
H	-0.810189000	-5.452938000	-0.310530000
H	-2.748634000	-3.339761000	-1.418963000
H	-0.997706000	-3.438186000	-1.715234000
H	-2.002639000	-1.842659000	0.314332000

[UO_2Cl_2(pynhfu)(thf)](thf)

C	-4.618459000	-6.030488000	7.913608000
C	-5.433573000	-7.121986000	7.611867000
C	-5.938925000	-7.249614000	6.315793000
C	-5.589674000	-6.291548000	5.364921000
N	-4.793774000	-5.253438000	5.657247000
C	-4.323093000	-5.108467000	6.903383000
C	-3.466900000	-3.897284000	7.139628000
N	-4.022182000	-2.739157000	6.428151000
C	-3.297075000	-1.496801000	6.746688000
C	-3.179490000	-1.212102000	8.207811000
C	-2.117639000	-1.045015000	9.055020000
C	-2.669109000	-0.814624000	10.362636000
C	-4.025183000	-0.867602000	10.212109000
O	-4.344077000	-1.106757000	8.912097000
H	-3.369157000	-3.714509000	8.219609000
H	-2.454325000	-4.084708000	6.750148000
H	-3.827255000	-0.685103000	6.241295000
H	-2.288782000	-1.554014000	6.319115000
H	-1.069726000	-1.083513000	8.778711000
H	-2.127235000	-0.635596000	11.284398000
H	-4.856189000	-0.756381000	10.897384000
H	-4.218390000	-5.884529000	8.914765000
H	-6.586233000	-8.077809000	6.037141000
H	-5.925726000	-6.359122000	4.332940000
H	-5.679175000	-7.855605000	8.378149000
U	-4.162032000	-3.420291000	3.802141000
O	-5.948877000	-3.354306000	3.774190000
O	-2.404218000	-3.362437000	4.108010000
Cl	-4.014224000	-2.385906000	1.364523000
Cl	-3.983924000	-5.765259000	2.622758000
H	-5.010212000	-2.606340000	6.685720000
O	-4.291623000	-0.870929000	4.022450000

C	-5.545434000	-0.211151000	3.694843000
H	-6.015938000	0.094215000	4.640377000
H	-6.187424000	-0.932769000	3.184790000
C	-5.155356000	0.978213000	2.824323000
H	-5.091094000	0.657404000	1.777769000
H	-5.873266000	1.802622000	2.898934000
C	-3.759513000	1.330194000	3.354905000
H	-3.832306000	1.901583000	4.290915000
H	-3.160785000	1.911400000	2.644984000
C	-3.165289000	-0.050817000	3.604506000
H	-2.412389000	-0.084618000	4.397632000
H	-2.754786000	-0.488717000	2.687918000
H	-7.479960000	-2.110245000	5.153611000
O	-6.762228000	-2.277964000	7.065724000
C	-7.145374000	-3.006599000	8.227851000
C	-8.000957000	-4.192932000	7.725867000
C	-8.338216000	-3.813614000	6.260649000
C	-7.851943000	-2.364935000	6.148053000
H	-6.232015000	-3.304706000	8.754337000
H	-7.726096000	-2.352623000	8.899334000
H	-8.900466000	-4.315149000	8.340306000
H	-7.442105000	-5.132782000	7.768872000
H	-9.404213000	-3.913956000	6.028086000
H	-7.778604000	-4.436184000	5.555249000
H	-8.638930000	-1.649417000	6.443699000

[$UO_2(NO_3)_2$(pynhfu)]

C	-3.301213000	-6.364246000	6.846037000
C	-3.983054000	-7.567708000	6.689539000
C	-4.754654000	-7.754775000	5.544619000
C	-4.799774000	-6.741238000	4.591780000
N	-4.128831000	-5.581474000	4.729644000
C	-3.396966000	-5.393129000	5.847867000
C	-2.700611000	-4.066996000	5.982186000
N	-3.454778000	-3.036190000	5.253331000
C	-3.058347000	-1.654368000	5.650362000
C	-4.043766000	-0.654955000	5.178828000
C	-4.004761000	0.385633000	4.294756000
C	-5.318317000	0.953567000	4.273780000
C	-6.070169000	0.216707000	5.140521000
O	-5.310033000	-0.768116000	5.713216000
H	-2.592990000	-3.813982000	7.053307000
H	-1.684271000	-4.124199000	5.556869000
H	-2.077390000	-1.425329000	5.219288000
H	-2.972286000	-1.615277000	6.754169000
H	-3.142215000	0.692511000	3.711708000
H	-5.659959000	1.794720000	3.679637000
H	-7.108015000	0.249991000	5.451622000
H	-2.701692000	-6.167500000	7.735724000
H	-5.317924000	-8.673056000	5.381068000
H	-5.376481000	-6.854522000	3.674437000
H	-3.921691000	-8.342134000	7.455434000
U	-3.655213000	-3.810864000	2.706893000
O	-5.195402000	-2.911139000	2.855262000

O	-2.124383000	-4.741680000	2.703935000
O	-2.190309000	-1.830932000	2.958435000
O	-4.141480000	-4.486755000	0.366702000
H	-4.447948000	-3.134389000	5.497056000
N	-2.210072000	-1.458701000	1.717131000
O	-2.883193000	-2.236495000	0.949411000
O	-1.646594000	-0.457518000	1.331713000
N	-4.834710000	-5.530659000	0.643106000
O	-5.338834000	-6.244689000	-0.192419000
O	-4.950616000	-5.759836000	1.917735000

[$UO_2(NO_3)_2$(pynhfu)(thf)]

C	-2.273618000	-6.340737000	5.396528000
C	-2.702788000	-7.667581000	5.366853000
C	-3.904517000	-7.999975000	5.988617000
C	-4.627771000	-6.986494000	6.621347000
N	-4.221433000	-5.713456000	6.664915000
C	-3.065120000	-5.399796000	6.056589000
C	-2.707461000	-3.935750000	6.124724000
N	-3.802224000	-3.114223000	5.541238000
C	-3.950746000	-1.823508000	6.260130000
C	-5.170177000	-1.088310000	5.844339000
C	-5.429918000	0.202837000	5.476945000
C	-6.836357000	0.283156000	5.222959000
C	-7.336295000	-0.964891000	5.450640000
O	-6.336781000	-1.813027000	5.841845000
H	-2.575240000	-3.662732000	7.188537000
H	-1.765832000	-3.732674000	5.599447000
H	-3.070207000	-1.198394000	6.063015000
H	-3.975785000	-2.028374000	7.349366000
H	-4.703074000	1.003797000	5.389297000
H	-7.398786000	1.153271000	4.900846000
H	-8.323270000	-1.406237000	5.374687000
H	-1.361819000	-6.029944000	4.886677000
H	-4.285102000	-9.021712000	5.977212000
H	-5.580529000	-7.205219000	7.111538000
H	-2.115208000	-8.426183000	4.847563000
U	-3.735177000	-3.102988000	2.825080000
O	-5.003035000	-1.844232000	2.747840000
O	-2.390624000	-4.281688000	2.979572000
O	-2.386778000	-1.786506000	1.189546000
O	-5.181350000	-4.822616000	1.770239000
H	-4.665186000	-3.648277000	5.722829000
N	-2.832927000	-2.357952000	0.113379000
O	-3.696900000	-3.279289000	0.337646000
O	-2.459549000	-2.041434000	-0.994199000
N	-5.734611000	-5.260659000	2.844640000
O	-6.548781000	-6.155767000	2.862555000
O	-5.337389000	-4.654536000	3.917749000
O	-2.068456000	-1.380954000	3.785825000
C	-0.631038000	-1.661870000	3.684597000
H	-0.463174000	-2.228661000	2.758633000
H	-0.355697000	-2.282711000	4.547580000
C	0.049964000	-0.301574000	3.652638000

H	0.239271000	0.065996000	4.672646000
H	1.007149000	-0.338771000	3.117204000
C	-0.998432000	0.569083000	2.949291000
H	-0.988807000	0.385399000	1.866537000
H	-0.860093000	1.642532000	3.132063000
C	-2.292664000	0.049586000	3.550478000
H	-3.170701000	0.148450000	2.902250000
H	-2.505622000	0.515914000	4.526467000

[$UO_2(NO_3)_2$(pynhfu)(thf)$_2$]

C	-1.187427000	-6.581396000	5.024358000
C	-1.196477000	-7.976190000	5.001084000
C	-2.375048000	-8.647418000	5.331466000
C	-3.495359000	-7.886315000	5.675137000
N	-3.490013000	-6.552909000	5.706868000
C	-2.360005000	-5.909227000	5.384736000
C	-2.439903000	-4.404270000	5.398726000
N	-3.625857000	-3.950939000	4.645198000
C	-3.808115000	-2.482984000	4.783815000
C	-5.218199000	-2.038169000	4.596233000
C	-6.354194000	-2.220893000	5.344605000
C	-7.403194000	-1.505044000	4.676436000
C	-6.824049000	-0.937398000	3.574391000
O	-5.508239000	-1.258270000	3.521149000
H	-2.510523000	-4.057421000	6.444858000
H	-1.551848000	-3.955099000	4.949969000
H	-3.154548000	-1.974678000	4.069190000
H	-3.480794000	-2.179841000	5.792060000
H	-6.428088000	-2.782989000	6.269451000
H	-8.440818000	-1.418991000	4.978596000
H	-7.196728000	-0.307609000	2.776484000
H	-0.305889000	-6.014930000	4.733618000
H	-2.433042000	-9.733787000	5.312728000
H	-4.437371000	-8.378314000	5.926620000
H	-0.304503000	-8.529584000	4.711396000
U	-3.707764000	-4.892833000	2.112584000
O	-4.103341000	-3.218286000	1.675522000
O	-3.333160000	-6.582174000	2.519389000
O	-1.502453000	-4.321763000	2.550704000
O	-4.658670000	-7.290544000	-0.793593000
H	-4.431700000	-4.426386000	5.052167000
N	-0.921953000	-3.164468000	2.260019000
O	-0.422947000	-3.047151000	1.156741000
O	-0.912386000	-2.312002000	3.134944000
N	-5.330652000	-6.907731000	0.146670000
O	-6.120725000	-7.592165000	0.776279000
O	-5.207711000	-5.635222000	0.504911000
O	-2.479210000	-5.108142000	0.025545000
C	-2.841172000	-4.425435000	-1.202329000
H	-2.577054000	-3.366808000	-1.084800000
H	-3.921071000	-4.532839000	-1.330171000
C	-2.014306000	-5.118368000	-2.278817000
H	-2.546776000	-6.007354000	-2.639060000
H	-1.812135000	-4.460724000	-3.131510000

C	-0.748095000	-5.522394000	-1.509861000
H	-0.083718000	-4.658961000	-1.380955000
H	-0.186164000	-6.328630000	-1.994354000
C	-1.309509000	-5.945642000	-0.157436000
H	-0.637637000	-5.756780000	0.683125000
H	-1.643873000	-6.990834000	-0.149220000
H	-7.127527000	-4.725571000	1.629409000
O	-5.911581000	-5.216696000	3.234439000
C	-6.318963000	-6.473364000	3.858067000
C	-7.818643000	-6.579559000	3.597709000
C	-8.247349000	-5.111727000	3.462123000
C	-7.072619000	-4.516658000	2.703761000
H	-5.742376000	-7.282554000	3.403942000
H	-6.066262000	-6.399380000	4.922315000
H	-8.338948000	-7.112879000	4.401120000
H	-7.986366000	-7.111035000	2.653985000
H	-8.346052000	-4.634559000	4.446466000
H	-9.192221000	-4.986290000	2.921970000
H	-6.920164000	-3.449422000	2.868115000

[$UO_2(NO_3)_2$(pynhfu)(thf)](thf)

C	-1.011160000	-6.222958000	5.306969000
C	-0.796275000	-7.601693000	5.306084000
C	-1.842819000	-8.448386000	5.677759000
C	-3.060945000	-7.869958000	6.045258000
N	-3.267728000	-6.552277000	6.060971000
C	-2.268525000	-5.741575000	5.692200000
C	-2.567286000	-4.261689000	5.727244000
N	-3.772342000	-3.934187000	4.935167000
C	-4.304012000	-2.599430000	5.277255000
C	-5.359535000	-2.181754000	4.305405000
C	-6.403665000	-2.845856000	3.706542000
C	-7.013981000	-1.899876000	2.812787000
C	-6.306595000	-0.741146000	2.946354000
O	-5.300786000	-0.903130000	3.848214000
H	-2.733094000	-3.968718000	6.778301000
H	-1.715849000	-3.691085000	5.343324000
H	-3.488689000	-1.868708000	5.219200000
H	-4.678516000	-2.591635000	6.315399000
H	-6.707077000	-3.865777000	3.913358000
H	-7.850551000	-2.083610000	2.149700000
H	-6.377622000	0.234530000	2.482727000
H	-0.240402000	-5.524351000	4.992603000
H	-1.723600000	-9.530152000	5.682577000
H	-3.902014000	-8.500394000	6.340967000
H	0.168976000	-8.008382000	5.007793000
U	-3.640056000	-4.186638000	2.347401000
O	-3.254369000	-2.473694000	2.073853000
O	-3.992105000	-5.907726000	2.617952000
O	-1.442572000	-4.574043000	2.786648000
O	-5.799316000	-5.756499000	-0.380582000
H	-4.498010000	-4.640577000	5.139330000
N	-0.409679000	-3.748808000	2.623100000
O	-0.033492000	-3.538928000	1.488133000

O	0.089627000	-3.294369000	3.639163000
N	-6.187052000	-5.155663000	0.605249000
O	-7.175889000	-5.427104000	1.261192000
O	-5.476579000	-4.096862000	0.988893000
O	-2.777485000	-4.753431000	0.174803000
C	-2.870219000	-3.886752000	-0.986880000
H	-2.065635000	-3.144729000	-0.911362000
H	-3.843468000	-3.389752000	-0.951101000
C	-2.698281000	-4.829696000	-2.171814000
H	-3.666779000	-5.270528000	-2.437488000
H	-2.290570000	-4.318392000	-3.050720000
C	-1.757576000	-5.903778000	-1.604714000
H	-0.721094000	-5.542901000	-1.595374000
H	-1.791999000	-6.845394000	-2.163436000
C	-2.272111000	-6.067388000	-0.178548000
H	-1.502883000	-6.331996000	0.553074000
H	-3.108792000	-6.773325000	-0.113548000
H	-5.454986000	-7.361184000	4.306427000
O	-6.142432000	-5.592547000	5.156865000
C	-6.485913000	-5.789294000	6.536432000
C	-6.998446000	-7.232025000	6.649522000
C	-7.435438000	-7.546363000	5.211272000
C	-6.388964000	-6.790036000	4.399196000
H	-5.593279000	-5.620784000	7.151946000
H	-7.258728000	-5.054729000	6.806197000
H	-7.804881000	-7.334207000	7.384540000
H	-6.181504000	-7.899580000	6.951907000
H	-8.436912000	-7.141209000	5.013017000
H	-7.451710000	-8.618939000	4.986398000
H	-6.718555000	-6.497491000	3.397399000

Literature-based structures

[$UO_2(acac)_2$(thf)]

U	-1.31342	-2.50621	3.10643
O	-2.04589	-1.45422	1.98865
O	-0.57611	-3.55651	4.21824
C	-0.83815	0.93866	6.33834
C	-0.23017	0.11124	5.14392
C	1.07605	0.23726	4.52680
C	1.39957	-0.44073	3.47173
C	2.76474	-0.09373	2.79489
O	-1.22399	-0.69276	4.70596
O	0.67169	-1.28314	2.83470
C	1.06414	-4.70413	-0.37034
C	-0.22901	-4.40458	0.46576
C	-1.40967	-4.94186	0.19702
C	-2.50433	-4.90554	0.96344
C	-3.88221	-5.62122	0.71460
O	0.04543	-3.65816	1.50093
O	-2.60430	-4.31657	2.16781
O	-3.20731	-2.73598	4.31921
C	-4.21230	-2.53511	6.40176
C	-4.34638	-1.20649	5.71795

C	-4.08041	-1.61822	4.27661
H	-3.51762	-0.55511	6.07188
H	-5.31090	-0.67758	5.87626
H	-5.02903	-1.98520	3.82520
H	-3.70498	-0.76472	3.68015
H	-0.09310	1.59898	6.73049
H	-1.16194	0.26917	7.10765
H	-1.67243	1.50992	5.98828
H	1.76614	0.88432	4.93924
H	2.90923	-0.72187	1.94080
H	3.55969	-0.25281	3.49321
H	2.76001	0.93108	2.48723
H	0.81158	-5.31617	-1.21089
H	1.77492	-5.21689	0.24348
H	1.48793	-3.78365	-0.71388
H	-1.49345	-5.44274	-0.70138
H	-4.38942	-5.75272	1.64752
H	-3.71166	-6.57642	0.26360
H	-4.50520	-5.00202	0.03641
C	-2.98204	-3.04962	5.67950
H	-5.08235	-3.17785	6.13060
H	-4.12097	-2.48935	7.50744
H	-2.08138	-2.51874	6.05585
H	-2.86329	-4.14208	5.84025

$[UO_2(acac)_2(thf)_2]$

U	8.21649	0.43781	5.56139
O	9.83792	0.23189	5.84263
O	8.82623	1.81130	3.71441
O	6.30035	1.49357	4.67270
O	8.29049	1.79485	6.69478
C	8.39543	2.96234	3.36201
C	7.14639	3.43707	3.65799
H	6.96220	4.32921	3.47132
C	6.12836	2.66055	4.22432
C	9.35472	3.79311	2.55537
H	10.17433	3.90070	3.04408
H	8.96819	4.65398	2.38421
H	9.53630	3.35178	1.72151
C	4.72956	3.20479	4.27927
H	4.22033	2.71335	4.92690
H	4.31933	3.11878	3.41580
H	4.75734	4.13152	4.52940
O	8.82623	-0.93568	7.40837
O	6.30035	-0.61795	6.45008
O	8.29049	-0.91923	4.42800
C	8.39543	-2.08672	7.76078
C	7.14639	-2.56145	7.46479
H	6.96220	-3.45359	7.65146
C	6.12836	-1.78493	6.89846
C	9.35472	-2.91749	8.56741
H	10.17433	-3.02508	8.07870
H	8.96819	-3.77836	8.73857
H	9.53630	-2.47616	9.40127

C	4.72956	-2.32917	6.84351
H	4.22033	-1.83773	6.19588
H	4.31933	-2.24316	7.70698
H	4.75734	-3.25590	6.59338
C	10.31502	1.53520	5.49286
C	11.06872	2.10396	6.68536
H	9.53489	2.27162	5.24656
H	11.01487	1.40761	4.63786
C	11.59725	0.84276	7.27327
H	10.33851	2.54105	7.40738
H	11.82105	2.88136	6.43375
C	10.34136	0.00381	7.15539
H	11.99360	0.94125	8.30637
H	12.38828	0.42370	6.60906
H	9.90071	0.59912	8.06837
H	10.90426	-0.98369	7.27529
O	6.18892	-0.71013	4.14545
C	6.29010	-2.07111	3.65606
C	4.88846	-2.55844	3.33188
H	6.74977	-2.77624	4.38180
H	6.87786	-2.05000	2.70931
C	4.30628	-1.29752	2.80447
H	4.34538	-2.83508	4.25246
H	4.85237	-3.42608	2.63924
C	4.83744	-0.29674	3.82180
H	3.20211	-1.29422	2.68188
H	4.77662	-1.09250	1.81359
H	4.21053	-0.28895	4.73254
H	4.74919	0.66119	3.27584

[UO$_2$(salophen)(thf)]

U	-3.20072	-4.68745	-0.14453
O	-1.71275	-3.71604	-0.02573
O	-4.62739	-5.76250	-0.23157
O	-3.27882	-4.29549	-2.36370
O	-3.59769	-4.28889	2.05838
N	-1.77505	-6.39094	-1.37911
N	-1.96041	-6.39754	1.27619
C	-2.29031	-4.10705	-3.21964
C	-2.34088	-3.04708	-4.14248
H	-3.07175	-2.44124	-4.11847
C	-1.33997	-2.86900	-5.08934
H	-1.40113	-2.14822	-5.70513
C	-0.24312	-3.73771	-5.14937
H	0.44788	-3.60768	-5.78918
C	-0.19108	-4.77601	-4.26255
H	0.54166	-5.37996	-4.31058
C	-1.18455	-4.98989	-3.27453
C	-1.06851	-6.18272	-2.44089
H	-0.43681	-6.84508	-2.69476
C	-1.68263	-7.66857	-0.75474
C	-1.54103	-8.86139	-1.42886
H	-1.50167	-8.86422	-2.37914
C	-1.45781	-10.04856	-0.73759

H	-1.34275	-10.86545	-1.20758
C	-1.54146	-10.05422	0.64496
H	-1.48798	-10.87581	1.12010
C	-1.70181	-8.86516	1.33794
H	-1.77683	-8.87647	2.28480
C	-1.75378	-7.65820	0.65525
C	-1.40691	-6.14691	2.40315
H	-0.80131	-6.79609	2.74107
C	-1.62529	-4.95691	3.20935
C	-0.71446	-4.71948	4.27456
H	0.02729	-5.30270	4.38434
C	-0.87486	-3.66610	5.15623
H	-0.25074	-3.50781	5.85436
C	-2.00384	-2.83508	4.97956
H	-2.14222	-2.10959	5.57820
C	-2.90525	-3.04990	3.96409
H	-3.66270	-2.48270	3.88518
C	-2.72527	-4.10140	3.03611
O	-3.93792	-2.59309	0.22485
C	-5.34968	-2.49585	0.14224
C	-5.65684	-1.27565	-0.71563
H	-5.71142	-2.31288	1.17791
H	-5.83419	-3.40562	-0.26636
C	-4.42949	-0.45131	-0.45503
H	-6.62943	-0.78612	-0.49048
H	-5.64035	-1.56989	-1.78904
C	-3.38845	-1.55189	-0.54824
H	-4.45732	-0.07135	0.59491
H	-4.27780	0.40236	-1.14692
H	-2.42209	-1.19372	-0.13310
H	-3.26194	-1.87619	-1.60198

[UO_2(salophen)(thf)$_2$]

U	2.12791	-0.45998	10.56688
O	1.88349	0.61146	11.98216
O	0.03580	-1.23504	10.74406
O	4.34565	-0.40945	10.95443
C	-2.20476	-1.08301	7.86320
H	-2.41511	-0.51241	7.15973
O	1.89942	-2.80507	11.11840
N	0.56674	0.95401	9.13480
C	0.14239	3.35956	8.68726
H	-0.77736	3.22677	8.71138
O	2.39352	-1.51601	9.14284
C	7.54794	-1.33415	9.55018
H	8.25582	-1.90667	9.74179
C	2.02622	4.82983	8.43267
H	2.36494	5.68140	8.27992
C	-1.18284	-0.71833	8.75962
C	4.37806	1.29849	8.60552
H	4.56896	1.90853	7.93020
C	5.40434	0.35647	8.94185
C	6.45004	-1.32164	10.36620

H	6.42423	-1.88742	11.10450
C	0.65446	4.62969	8.46483
H	0.07631	5.34751	8.33754
C	1.00213	2.28380	8.87351
C	-1.62484	-2.75727	9.98700
H	-1.45519	-3.32594	10.70386
C	6.56932	0.32568	8.13923
H	6.62069	0.87992	7.39422
C	-0.88247	-1.56316	9.85300
C	-0.51915	0.54699	8.55595
H	-0.90944	1.13010	7.94494
C	-2.60482	-3.09405	9.06646
H	-3.06932	-3.89365	9.16562
N	3.20570	1.37546	9.15758
C	-2.90373	-2.26461	8.00121
H	-3.56285	-2.49940	7.38886
C	2.89224	3.77235	8.62830
H	3.81081	3.91091	8.60686
C	5.36098	-0.47104	10.10893
C	2.38361	2.49934	8.85475
C	7.62211	-0.49702	8.43133
H	8.37892	-0.49990	7.89134
C	3.14076	-3.44682	11.38659
C	3.10285	-4.85282	10.81778
H	3.28476	-3.63732	12.45293
H	3.95499	-2.89077	10.88128
C	2.39715	-4.57156	9.55441
H	2.45102	-5.49892	11.45602
H	4.09606	-5.34023	10.72065
C	1.31565	-3.66436	10.10988
H	2.01907	-5.46904	9.02011
H	3.07307	-4.00015	8.87792
H	0.55478	-4.29761	10.61655
H	0.84553	-3.17697	9.24220
O	3.37422	-0.57037	12.91205
C	4.01416	0.66349	13.32641
C	4.18157	-1.60937	13.46412
C	5.40909	0.31440	13.80216
H	3.42205	1.08564	14.17027
H	4.08567	1.43459	12.52749
C	5.08997	-0.96076	14.50227
H	5.89126	1.09619	14.42684
H	6.06377	0.09650	12.92875
H	4.49915	-0.71983	15.41755
H	5.96901	-1.57317	14.79665
H	4.85414	-2.03975	12.70400
H	3.57680	-2.35091	14.02340

[UO_2(salen)(thf)]

U	3.88435	20.10189	11.00133
O	5.13502	18.21824	11.07773
O	2.63747	21.81516	11.78310
O	2.37146	19.20485	10.69091
O	5.39715	21.01879	11.27551

O	3.66549	19.58817	13.33213
N	3.61812	21.93042	9.18914
N	4.35055	19.23445	8.60979
C	2.56796	23.77987	10.40386
C	5.64339	17.21101	10.39685
C	1.69552	23.80906	12.66176
H	1.50680	23.37727	13.46515
C	4.18268	21.54020	7.87290
H	5.14977	21.60868	7.89566
H	3.85119	22.13373	7.18153
C	1.60529	25.79893	11.32802
H	1.37810	26.69574	11.23875
C	6.38515	16.19991	11.04972
H	6.54063	16.25789	11.96513
C	2.18965	25.11803	10.28659
H	2.33735	25.56160	9.48319
C	2.31875	23.09147	11.62907
C	4.83115	18.12733	8.18796
H	4.76333	17.96287	7.27605
C	6.88286	15.12172	10.33384
H	7.35246	14.45879	10.78542
C	5.47224	17.09827	8.99135
C	3.18553	23.13721	9.26965
H	3.28173	23.65853	8.50477
C	1.35935	25.12491	12.51823
H	0.95952	25.57778	13.22536
C	3.77326	20.11293	7.56835
H	2.80645	20.03643	7.56835
H	4.10221	19.85143	6.69319
C	6.70245	15.00324	8.97385
H	7.08804	14.19574	8.45975
C	5.98742	15.99771	8.30523
H	5.83725	15.91803	7.28733
C	4.43093	18.50623	13.82020
C	4.63226	18.81851	15.29269
H	5.43612	18.51133	13.34969
H	3.92467	17.52821	13.67918
C	4.92886	20.28842	15.18349
H	5.42222	18.21570	15.78915
H	3.66759	18.68041	15.83336
C	3.85221	20.69994	14.19034
H	5.92848	20.40390	14.70287
H	4.92436	20.85942	16.13599
H	4.16484	21.62539	13.66669
H	2.89115	20.86909	14.72405

[UO_2(salen)(thf)$_2$]

U	9.60330	3.00519	6.95913
N	9.75777	1.88362	9.24792
O	10.94993	4.07238	7.44747
O	8.21053	1.95057	6.54843
O	9.78785	1.84183	4.84161
O	11.03272	1.25748	6.83749
O	8.57112	4.78718	6.06351

N	8.03575	4.01990	8.70755
C	6.46276	5.06949	7.14543
C	13.11165	0.15270	7.02155
H	13.21468	0.18798	6.07767
C	7.31089	5.20973	6.02221
C	11.89005	0.68563	9.01195
C	9.44633	0.44395	4.81697
C	8.30436	3.66611	10.09859
H	7.51142	3.86155	10.65902
H	9.06786	4.19453	10.44193
C	11.98572	0.74083	7.58080
C	5.15101	5.56171	7.05104
H	4.56229	5.44680	7.78727
C	10.71628	1.16247	9.70453
H	10.64453	0.91093	10.61773
C	8.96341	2.45121	3.84210
C	12.88352	0.04502	9.71986
H	12.81913	0.01064	10.66728
C	6.82335	5.89197	4.91079
H	7.39000	6.02317	4.16041
C	8.63000	2.18311	10.15641
H	8.86916	1.92886	11.08259
H	7.83522	1.65651	9.88740
C	13.94690	-0.53678	9.12050
H	14.60554	-0.98919	9.63491
C	5.54840	6.37514	4.87776
H	5.24300	6.84113	4.10850
C	4.70211	6.19508	5.94198
H	3.80798	6.51358	5.90187
C	6.93006	4.57817	8.44680
H	6.33397	4.69218	9.17831
C	14.05913	-0.45896	7.73300
H	14.81159	-0.84170	7.29527
C	8.47385	1.33388	2.93901
H	9.50377	3.20181	3.22826
H	8.08588	2.93110	4.32406
C	8.19255	0.29323	3.97126
H	7.61241	1.60543	2.29254
H	9.31673	0.98666	2.29706
H	8.02057	-0.73208	3.58049
H	7.30057	0.60729	4.55961
H	9.30013	-0.03118	5.80946
H	10.29136	-0.08399	4.32057
O	10.80278	3.98263	5.11537
C	11.09059	5.39000	5.17876
C	12.06502	3.34574	4.89373
C	12.60066	5.54709	5.23607
H	10.62477	5.94455	6.01836
H	10.71034	5.83656	4.23279
C	12.99561	4.41279	4.34924
H	12.95915	5.33289	6.26916
H	12.97266	6.54722	4.92801
H	14.07314	4.14458	4.37831
H	12.70561	4.65241	3.29974

H	12.01252	2.50518	4.17088
H	12.48078	2.98827	5.85821

19 References

(1) Stukowski, A. Visualization and Analysis of Atomistic Simulation Data with OVITO–the Open Visualization Tool. *Model. Simul. Mater. Sci. Eng.* **2010**, *18* (1), 015012. https://doi.org/10.1088/0965-0393/18/1/015012.

(2) Maglic, J. B.; Lavendomme, R. MoloVol : An Easy-to-Use Program for Analyzing Cavities, Volumes and Surface Areas of Chemical Structures. *J. Appl. Crystallogr.* **2022**, *55* (4), 1033–1044. https://doi.org/10.1107/S1600576722004988.

(3) Evans, R. The Interpretation of Small Molecule Diffusion Coefficients: Quantitative Use of Diffusion-Ordered NMR Spectroscopy. *Prog. Nucl. Magn. Reson. Spectrosc.* **2020**, *117*, 33–69. https://doi.org/10.1016/j.pnmrs.2019.11.002.

(4) Dagdug, L.; Peña, J.; Pompa-García, I. *Diffusion Under Confinement*; Springer International Publishing: Cham, 2024. https://doi.org/10.1007/978-3-031-46475-1.

(5) Simons, J.; Ponter, A. B. Semi-empirical Formulae for the Prediction of Diffusion in Liquid Systems. *Can. J. Chem. Eng.* **1975**, *53* (5), 541–550. https://doi.org/10.1002/cjce.5450530514.

(6) Spernol, A.; Wirtz, K. Zur Mikroreibung in Flüssigkeiten. *Z. Naturforsch. A* **1953**, *8* (9), 522–532. https://doi.org/10.1515/zna-1953-0902.

(7) Gierer, A.; Wirtz, K. Molekulare Theorie Der Mikroreibung. *Z. Naturforsch. A* **1953**, *8* (9), 532–538. https://doi.org/10.1515/zna-1953-0903.

(8) Chen, H. C.; Chen, S. H. Diffusion of Crown Ethers in Alcohols. *J. Phys. Chem.* **1984**, *88* (21), 5118–5121. https://doi.org/10.1021/j150665a063.

(9) Bondi, A. Van Der Waals Volumes and Radii. *J. Phys. Chem.* **1964**, *68* (3), 441–451. https://doi.org/10.1021/j100785a001.

(10) Bryant, K.; Hammond-Pereira, E.; Saunders, S. R. Ionic Liquid Aggregation Mechanism for Nanoparticle Synthesis. *J. Phys. Chem. B* **2021**, *125* (1), 253–263. https://doi.org/10.1021/acs.jpcb.0c08908.

(11) Tang, B.; Chong, K.; Massefski, W.; Evans, R. Quantitative Interpretation of Protein Diffusion Coefficients in Mixed Protiated–Deuteriated Aqueous Solvents. *J. Phys. Chem. B* **2022**, *126* (31), 5887–5895. https://doi.org/10.1021/acs.jpcb.2c03554.

(12) Graham, T. R.; Pope, D. J.; Ghadar, Y.; Clark, S.; Clark, A.; Saunders, S. R. Alcohol Clustering Mechanisms in Supercritical Carbon Dioxide Using Pulsed-Field Gradient, Diffusion NMR and Network Analysis: Feedback on Stepwise Self-Association Models. *J. Phys. Chem. B* **2019**, *123* (25), 5316–5323. https://doi.org/10.1021/acs.jpcb.9b02305.

(13) Evans, R.; Deng, Z.; Rogerson, A. K.; McLachlan, A. S.; Richards, J. J.; Nilsson, M.; Morris, G. A. Quantitative Interpretation of Diffusion-Ordered NMR Spectra: Can We Rationalize Small Molecule Diffusion Coefficients? *Angew. Chemie Int. Ed.* **2013**, *52* (11), 3199–3202. https://doi.org/10.1002/anie.201207403.

(14) Evans, R.; Dal Poggetto, G.; Nilsson, M.; Morris, G. A. Improving the Interpretation of Small Molecule Diffusion Coefficients. *Anal. Chem.* **2018**, *90* (6), 3987–3994. https://doi.org/10.1021/acs.analchem.7b05032.

(15) Neufeld, R.; Stalke, D. Accurate Molecular Weight Determination of Small Molecules via DOSY-NMR by Using External Calibration Curves with Normalized Diffusion Coefficients. *Chem. Sci.*

2015, *6* (6), 3354–3364. https://doi.org/10.1039/C5SC00670H.

(16) Bachmann, S.; Neufeld, R.; Dzemski, M.; Stalke, D. New External Calibration Curves (ECCs) for the Estimation of Molecular Weights in Various Common NMR Solvents. *Chem. Eur. J.* **2016**, *22* (25), 8462–8465. https://doi.org/10.1002/chem.201601145.

(17) Kreyenschmidt, A.; Bachmann, S.; Niklas, T.; Stalke, D. Molecular Weight Estimation of Molecules Incorporating Heavier Elements from Van-der-Waals Corrected ECC-DOSY. *ChemistrySelect* **2017**, *2* (24), 6957–6960. https://doi.org/10.1002/slct.201701497.

(18) Kreyenschmidt, A.-K.; John, M.; Stalke, D. Molecular Weight Determination of Relevant Organometallic Catalysts in Solution by DOSY-NMR to Monitor Reactivity. *Organometallics* **2023**, *42* (20), 2957–2962. https://doi.org/10.1021/acs.organomet.3c00265.

(19) Li, D.; Kagan, G.; Hopson, R.; Williard, P. G. Formula Weight Prediction by Internal Reference Diffusion-Ordered NMR Spectroscopy (DOSY). *J. Am. Chem. Soc.* **2009**, *131* (15), 5627–5634. https://doi.org/10.1021/ja810154u.

(20) Li, D.; Keresztes, I.; Hopson, R.; Williard, P. G. Characterization of Reactive Intermediates by Multinuclear Diffusion-Ordered NMR Spectroscopy (DOSY). *Acc. Chem. Res.* **2009**, *42* (2), 270–280. https://doi.org/10.1021/ar800127e.

(21) Keresztes, I.; Williard, P. G. Diffusion-Ordered NMR Spectroscopy (DOSY) of THF Solvated *n*-Butyllithium Aggregates. *J. Am. Chem. Soc.* **2000**, *122* (41), 10228–10229. https://doi.org/10.1021/ja002278x.

(22) Bachmann, S.; Gernert, B.; Stalke, D. Solution Structures of Alkali Metal Cyclopentadienides in THF Estimated by ECC-DOSY NMR-Spectroscopy (Incl. Software). *Chem. Commun.* **2016**, *52* (87), 12861–12864. https://doi.org/10.1039/C6CC07273A.

(23) Wilkerson, M. P.; Burns, C. J.; Paine, R. T.; Scott, B. L. Synthesis and Crystal Structure of $UO_2Cl_2(THF)_3$: A Simple Preparation of an Anhydrous Uranyl Reagent. *Inorg. Chem.* **1999**, *38* (18), 4156–4158. https://doi.org/10.1021/ic990159g.

(24) Nipruk, O. V.; Knyazev, A. V.; Chernorukov, G. N.; Pykhova, Y. P. Synthesis and Study of Hydrated Uranium(VI) Oxides, $UO_3 \cdot nH_2O$. *Radiochemistry* **2011**, *53* (2), 146–150. https://doi.org/10.1134/S1066362211020044.

(25) Mizuoka, K.; Kim, S.-Y.; Hasegawa, M.; Hoshi, T.; Uchiyama, G.; Ikeda, Y. Electrochemical and Spectroelectrochemical Studies on UO_2(Saloph)L (Saloph = *N,N*'-Disalicylidene-*o*-Phenylenediaminate, L = Dimethyl Sulfoxide or *N,N*-Dimethylformamide). *Inorg. Chem.* **2003**, *42* (4), 1031–1038. https://doi.org/10.1021/ic0260926.

(26) Evans, D. J.; Junk, P. C.; Smith, M. K. The Effect of Coordinated Solvent Ligands on the Solid-State Structures of Compounds Involving Uranyl Nitrate and Schiff Bases. *Polyhedron* **2002**, *21* (23), 2421–2431. https://doi.org/10.1016/S0277-5387(02)01182-8.

(27) Taylor, J. C.; McLaren, A. B. Crystal and Molecular Structure of Bis(3-Chloropentane-2,4-Dionato)-Dioxo(Triphenylphosphine Oxide)Uranium(VI) and Dioxobis(Pentane-2,4-Dionato)(Triphenylphosphine Oxide)Uranium(VI)–Benzene (1/1). *J. Chem. Soc., Dalt. Trans.* **1979**, No. 3, 460–464. https://doi.org/10.1039/DT9790000460.

(28) Jana, A.; Das, K.; Kundu, A.; Thorve, P. R.; Adhikari, D.; Maji, B. A Phosphine-Free Manganese Catalyst Enables Stereoselective Synthesis of (1+n)-Membered Cycloalkanes from Methyl

Ketones and 1,n -Diols. *ACS Catal.* **2020**, *10* (4), 2615–2626. https://doi.org/10.1021/acscatal.9b05567.
(29) Sheldrick, G. M. SHELXT – Integrated Space-Group and Crystal-Structure Determination. *Acta Cryst A* **2015**, *71* (1), 3–8. https://doi.org/10.1107/S2053273314026370.
(30) Sheldrick, G. M. Crystal Structure Refinement with SHELXL. *Acta Cryst. C* **2015**, *71* (1), 3–8. https://doi.org/10.1107/S2053229614024218.
(31) Merz, K.; Bieda, R. In Situ Crystallization of $N(SiMe_3)_3$ and $As(SiMe_3)_3$: Trigonal Planar or Pyramidal Coordination of the Central Atoms? *Z. Kristallogr. - Cryst. Mater.* **2014**, *229* (9), 635–638. https://doi.org/10.1515/zkri-2013-1719.
(32) Nakano, K.; Sada, K.; Nakagawa, K.; Aburaya, K.; Yoswathananont, N.; Tohnai, N.; Miyata, M. Organic Intercalation Material: Reversible Change in Interlayer Distances by Guest Release and Insertion in Sandwich-Type Inclusion Crystals of Cholic Acid. *Chem. Eur. J.* **2005**, *11* (6), 1725–1733. https://doi.org/10.1002/chem.200400809.
(33) Abrahams, S. C.; Robertson, J. M.; White, J. G. The Crystal and Molecular Structure of Naphthalene. I. X-Ray Measurements. *Acta Cryst.* **1949**, *2* (4), 233–238. https://doi.org/10.1107/S0365110X49000618.
(34) Meyer-Wegner, F.; Lerner, H.-W.; Bolte, M. C—H...π Interactions in Cocrystals of Bis(Trimethylsilyl)Acetylene and Diphenylacetylene with Benzene. *Acta Cryst. C* **2010**, *66* (4), o182–o184. https://doi.org/10.1107/S0108270110005846.
(35) Abrahams, S. C. The Crystal Structure of Dpihenyl Sulfoxide. *Acta Cryst.* **1957**, *10* (6), 417–422. https://doi.org/10.1107/S0365110X5700136X.
(36) Cameron, T. S.; Dahlèn, B. Investigation of Phosphorus–Carbon Bond Lengths in Aromatic Phosphines. Part I. Crystal and Molecular Structures of Tri-*o*-Tolylphosphine, -Phosphine Oxide, -Phosphine Sulphide, and -Phosphine Selenide. *J. Chem. Soc., Perkin Trans. 2* **1975**, No. 15, 1737–1751. https://doi.org/10.1039/P29750001737.
(37) Tomlins, P. E.; Lydon, J. E.; Akrigg, D.; Sheldrick, B. Hexaphenylcyclotrisiloxane, $C_{36}H_{30}O_3Si_3$, a Redetermination. *Acta Cryst. C* **1985**, *41* (2), 292–294. https://doi.org/10.1107/S0108270185003675.
(38) Jones, M. D.; Almeida Paz, F. A.; Davies, J. E.; Johnson, B. F. G. (*S*)-(−)-2,2′-Bis(Diphenylphosphino)-1,1′-Binaphthyl, a Versatile Chelating Ligand. *Acta Cryst. E* **2003**, *59* (4), o535–o537. https://doi.org/10.1107/S1600536803006019.
(39) Asher, M.; Angerer, D.; Korobko, R.; Diskin-Posner, Y.; Egger, D. A.; Yaffe, O. Anharmonic Lattice Vibrations in Small-Molecule Organic Semiconductors. *Adv. Mater.* **2020**, *32* (10). https://doi.org/10.1002/adma.201908028.
(40) Wang, S.; Shen, Y.; Zhang, X.; Liu, H.; Zhang, S.-T.; Li, W.; Yang, B. Noncovalent π–π Dimerization Based on Acridine and Acid-Responsive Luminescence Switching. *Dye. Pigment.* **2022**, *205*, 110527. https://doi.org/10.1016/j.dyepig.2022.110527.
(41) Cox, P. J.; Sim, G. A. X-Ray Diffraction Studies of 9-Methylanthracene and 5-Methylnaphthacene. *Acta Cryst. B* **1979**, *35* (2), 404–410. https://doi.org/10.1107/S0567740879003642.
(42) Gong, Y.; Zhang, P.; Gu, Y.; Wang, J.; Han, M.; Chen, C.; Zhan, X.; Xie, Z.; Zou, B.; Peng, Q.;

Chi, Z.; Li, Z. The Influence of Molecular Packing on the Emissive Behavior of Pyrene Derivatives: Mechanoluminescence and Mechanochromism. *Adv. Opt. Mater.* **2018**, *6* (16). https://doi.org/10.1002/adom.201800198.

(43) Rather, S. A.; Saha, B. K. Understanding the Elastic Bending Mechanism in a 9,10-Anthraquinone Crystal through Thermal Expansion Study. *CrystEngComm* **2021**, *23* (34), 5768–5773. https://doi.org/10.1039/D1CE00467K.

(44) Xia, Y.; Zhu, C.; Cao, F.; Shen, Y.; Ouyang, M.; Zhang, Y. Host–Guest Doping in Flexible Organic Crystals for Room-Temperature Phosphorescence. *Angew. Chemie Int. Ed.* **2023**, *62* (8). https://doi.org/10.1002/anie.202217547.

(45) Basu, S.; Chatterjee, S.; De, K.; Bhaumik, A.; Mukhopadhyay, C. Vanadium Incorporated Zeolite-Y, a Versatile Catalyst for Inter and Intra- Molecular Haloalkynylation Coupling Reactions. *J. Organomet. Chem.* **2022**, *979*, 122494. https://doi.org/10.1016/j.jorganchem.2022.122494.

(46) Jones, C. G.; Asay, M.; Kim, L. J.; Kleinsasser, J. F.; Saha, A.; Fulton, T. J.; Berkley, K. R.; Cascio, D.; Malyutin, A. G.; Conley, M. P.; Stoltz, B. M.; Lavallo, V.; Rodríguez, J. A.; Nelson, H. M. Characterization of Reactive Organometallic Species via MicroED. *ACS Cent. Sci.* **2019**, *5* (9), 1507–1513. https://doi.org/10.1021/acscentsci.9b00403.

(47) Fernando, N. K.; Cairns, A. B.; Murray, C. A.; Thompson, A. L.; Dickerson, J. L.; Garman, E. F.; Ahmed, N.; Ratcliff, L. E.; Regoutz, A. Structural and Electronic Effects of X-Ray Irradiation on Prototypical $[M(COD)Cl]_2$ Catalysts. *J. Phys. Chem. A* **2021**, *125* (34), 7473–7488. https://doi.org/10.1021/acs.jpca.1c05759.

(48) Scott, N. W. J.; Ford, M. J.; Schotes, C.; Parker, R. R.; Whitwood, A. C.; Fairlamb, I. J. S. The Ubiquitous Cross-Coupling Catalyst System ‘$Pd(OAc)_2$’/$2PPh_3$ Forms a Unique Dinuclear Pd I Complex: An Important Entry Point into Catalytically Competent Cyclic Pd_3 Clusters. *Chem. Sci.* **2019**, *10* (34), 7898–7906. https://doi.org/10.1039/C9SC01847F.

(49) Viciu, M. S.; Navarro, O.; Germaneau, R. F.; Kelly, R. A.; Sommer, W.; Marion, N.; Stevens, E. D.; Cavallo, L.; Nolan, S. P. Synthetic and Structural Studies of (NHC)Pd(Allyl)Cl Complexes (NHC = N -Heterocyclic Carbene). *Organometallics* **2004**, *23* (7), 1629–1635. https://doi.org/10.1021/om034319e.

(50) Straub, T.; Haukka, M.; Pakkanen, T. A. Unbridged Homo and Hetero Dinuclear Complexes of Group 6 and 8 Metals: Synthesis, Characterization and Comparison of X-Ray Crystallographic Data. *J. Organomet. Chem.* **2000**, *612* (1–2), 106–116. https://doi.org/10.1016/S0022-328X(00)00428-9.

(51) Straiton, A. J.; McMullin, C. L.; Kociok- Köhn, G.; Lyall, C. L.; Parish, J. D.; Johnson, A. L. Lithium, Tin(II), and Zinc Amino-Boryloxy Complexes: Synthesis and Characterization. *Inorg. Chem.* **2023**, *62* (6), 2576–2591. https://doi.org/10.1021/acs.inorgchem.2c03108.

(52) Pflock, S.; Langer, F.; Reil, M.; Strinitz, L.; Lorenz, R.; Hübner, E. G.; Burzlaff, N. P -Block Metal Complexes with Bis(Pyrazol-1-Yl)Acetato Ligands. *Dalt. Trans.* **2022**, *51* (10), 3902–3912. https://doi.org/10.1039/D1DT04040E.

(53) Hamdouni, N.; Boudjada, A.; Medjroubi, M. L. Spectroscopy, Crystal Structure and Molecular Conformation of 1-Bromo-2,3,5,6-Tetra-Methylbenzene. *J. Mol. Struct.* **2019**, *1198*, 126827. https://doi.org/10.1016/j.molstruc.2019.07.074.

(54) Li, L.; Wu, W. X.; Liu, Z. F.; Deng, X.; Jin, W. J. The Self-Assembled Dual-Band Room Temperature Phosphorescence Crystals with Grating Morphology Directed by Deoxycholate. *Cryst. Growth Des.* **2018**, *18* (10), 6307–6315. https://doi.org/10.1021/acs.cgd.8b01206.

(55) Mondal, R.; Nesterov, E. E.; Fronczek, F. R. Experimental Crystal Structure Determination 9-Bromoanthracene. *CCDC 864332* **2012**. https://doi.org/10.5517/ccy0dp0.

(56) Bodzioch, A.; Owsianik, K.; Skalik, J.; Kowalska, E.; Stasiak, A.; Różycka-Sokołowska, E.; Marciniak, B.; Bałczewski, P. Efficient Synthesis of Bis(Dibromomethyl)Arenes as Important Precursors of Synthetically Useful Dialdehydes. *Synthesis* **2016**, *48* (20), 3509–3514. https://doi.org/10.1055/s-0035-1561651.

(57) Ranjan, S.; Takamizawa, S. Two-Dimensional Organoferroelasticity in a Single Crystal of 4-Iodoaniline. *Cryst. Growth Des.* **2022**, *22* (3), 1831–1836. https://doi.org/10.1021/acs.cgd.1c01394.

(58) Goubitz, K.; Sonneveld, E. J.; Schenk, H. Crystal Structure Determination of a Series of Small Organic Compounds from Powder Data. *Z. Kristallogr. - Cryst. Mater.* **2001**, *216* (3), 176–181. https://doi.org/10.1524/zkri.216.3.176.20326.

(59) Churchill, M. R.; Julis, S. A. Dimeric (Pentamethylcyclopentadienyl)Rhodium and Iridium Complex. 4. Crystal and Molecular Structure of Di-μ-Bromo-Dibromobis(Pentamethylcyclopentadienyl)Dirhodium and Partially Halide-Exchanged Species $[(\eta5\text{-}C_5Me_5)RhX]_2(\mu\text{-}X')_2$ Obtained by Recrystallization of Bromo Complex from 1,2-Dichloroethane. *Inorg. Chem.* **1978**, *17* (11), 3011–3019. https://doi.org/10.1021/ic50189a009.

(60) Wadepohl, H. Experimental Crystal Structure Determination Bis(μ_2-Chloro)-Tetrakis(η_2-Ethene)-Di-Rhodium. *CCDC 731896* **2013**. https://doi.org/https://dx.doi.org/10.5517/ccsklkg.

(61) Torker, S.; Müller, A.; Sigrist, R.; Chen, P. Tuning the Steric Properties of a Metathesis Catalyst for Copolymerization of Norbornene and Cyclooctene toward Complete Alternation. *Organometallics* **2010**, *29* (12), 2735–2751. https://doi.org/10.1021/om100185g.

(62) Lehman, S. E.; Wagener, K. B. Synthesis of Ruthenium Olefin Metathesis Catalysts with Linear Alkyl Carbene Complexes. *Organometallics* **2005**, *24* (7), 1477–1482. https://doi.org/10.1021/om049176q.

(63) Pollard, V. A.; Kennedy, A. R.; McLellan, R.; Ross, D.; Tuttle, T.; Mulvey, R. E. Structurally Defined Ring-Opening and Insertion of Pinacolborane into Aluminium-Nitrogen Bonds of Sterically Demanding Dialkylaluminium Amides. *Eur. J. Inorg. Chem.* **2021**, *2021* (1), 50–53. https://doi.org/10.1002/ejic.202000919.

(64) Macdonald, P. A.; Kennedy, A. R.; Weetman, C. E.; Robertson, S. D.; Mulvey, R. E. Synthesis, Characterisation, and Catalytic Application of a Soluble Molecular Carrier of Sodium Hydride Activated by a Substituted 4-(Dimethylamino)Pyridine. *Commun. Chem.* **2024**, *7* (1), 94. https://doi.org/10.1038/s42004-024-01184-5.

(65) Hevia, E.; Dankert, F. Reductive-Transmetalation Reactions of $ZnR_2/(AlCp^*)_4$ Heterobimetallic Combinations and Application Towards CO_2 Insertion. *Eur. J. Inorg. Chem.* **2024**, *27* (31). https://doi.org/10.1002/ejic.202400418.

(66) Kieser, J. M.; Jones, L. O.; Uible, M. C.; Zeller, M.; Schatz, G. C.; Bart, S. C. Late to the Party: Synthesis and Characterization of Tellurium and Selenium Half-Sandwich Complexes.

Organometallics **2021**, *40* (24), 4104–4109. https://doi.org/10.1021/acs.organomet.1c00546.
(67) Kreyenschmidt, A.-K.; John, M.; Stalke, D. Molecular Weight Determination of Relevant Organometallic Catalysts in Solution by DOSY-NMR to Monitor Reactivity. *Organometallics* **2023**, *42* (20), 2957–2962. https://doi.org/10.1021/acs.organomet.3c00265.
(68) Holz, M.; Mao, X.; Seiferling, D.; Sacco, A. Experimental Study of Dynamic Isotope Effects in Molecular Liquids: Detection of Translation-Rotation Coupling. *J. Chem. Phys.* **1996**, *104* (2), 669–679. https://doi.org/10.1063/1.470863.
(69) Hiller, W.; Grabe, B.; Schonert, J. Molar Mass Determination for Small and Large Molecules Using Diffusion-Ordered Spectroscopy. *Anal. Chem.* **2024**, *96* (37), 14902–14908. https://doi.org/10.1021/acs.analchem.4c02874.
(70) Neese, F. The ORCA Program System. *WIREs Comput. Mol. Sci.* **2012**, *2* (1), 73–78. https://doi.org/10.1002/wcms.81.
(71) Neese, F. Software Update: The ORCA Program System, Version 4.0. *WIREs Comput. Mol. Sci.* **2018**, *8* (1). https://doi.org/10.1002/wcms.1327.
(72) Neese, F.; Wennmohs, F.; Becker, U.; Riplinger, C. The ORCA Quantum Chemistry Program Package. *J. Chem. Phys.* **2020**, *152* (22). https://doi.org/10.1063/5.0004608.
(73) Weigend, F.; Ahlrichs, R. Balanced Basis Sets of Split Valence, Triple Zeta Valence and Quadruple Zeta Valence Quality for H to Rn: Design and Assessment of Accuracy. *Phys. Chem. Chem. Phys.* **2005**, *7* (18), 3297. https://doi.org/10.1039/b508541a.
(74) Perdew, J. P.; Burke, K.; Ernzerhof, M. Generalized Gradient Approximation Made Simple. *Phys. Rev. Lett.* **1996**, *77* (18), 3865–3868. https://doi.org/10.1103/PhysRevLett.77.3865.
(75) Stephens, P. J.; Devlin, F. J.; Chabalowski, C. F.; Frisch, M. J. Ab Initio Calculation of Vibrational Absorption and Circular Dichroism Spectra Using Density Functional Force Fields. *J. Phys. Chem.* **1994**, *98* (45), 11623–11627. https://doi.org/10.1021/j100096a001.
(76) Vosko, S. H.; Wilk, L.; Nusair, M. Accurate Spin-Dependent Electron Liquid Correlation Energies for Local Spin Density Calculations: A Critical Analysis. *Can. J. Phys.* **1980**, *58* (8), 1200–1211. https://doi.org/10.1139/p80-159.
(77) Lee, C.; Yang, W.; Parr, R. G. Development of the Colle-Salvetti Correlation-Energy Formula into a Functional of the Electron Density. *Phys. Rev. B* **1988**, *37* (2), 785–789. https://doi.org/10.1103/PhysRevB.37.785.
(78) Becke, A. D. Density-Functional Thermochemistry. III. The Role of Exact Exchange. *J. Chem. Phys.* **1993**, *98* (7), 5648–5652. https://doi.org/10.1063/1.464913.
(79) Pantazis, D. A.; Neese, F. All-Electron Scalar Relativistic Basis Sets for the Actinides. *J. Chem. Theory Comput.* **2011**, *7* (3), 677–684. https://doi.org/10.1021/ct100736b.
(80) van Lenthe, E.; Ehlers, A.; Baerends, E.-J. Geometry Optimizations in the Zero Order Regular Approximation for Relativistic Effects. *J. Chem. Phys.* **1999**, *110* (18), 8943–8953. https://doi.org/10.1063/1.478813.
(81) van Lenthe, E.; Baerends, E. J.; Snijders, J. G. Relativistic Total Energy Using Regular Approximations. *J. Chem. Phys.* **1994**, *101* (11), 9783–9792. https://doi.org/10.1063/1.467943.
(82) Lenthe, E. van; Baerends, E. J.; Snijders, J. G. Relativistic Regular Two-Component Hamiltonians. *J. Chem. Phys.* **1993**, *99* (6), 4597–4610. https://doi.org/10.1063/1.466059.

(83) Grimme, S.; Antony, J.; Ehrlich, S.; Krieg, H. A Consistent and Accurate Ab Initio Parametrization of Density Functional Dispersion Correction (DFT-D) for the 94 Elements H-Pu. *J. Chem. Phys.* **2010**, *132* (15). https://doi.org/10.1063/1.3382344.

(84) Grimme, S.; Ehrlich, S.; Goerigk, L. Effect of the Damping Function in Dispersion Corrected Density Functional Theory. *J. Comput. Chem.* **2011**, *32* (7), 1456–1465. https://doi.org/10.1002/jcc.21759.

(85) Hanwell, M. D.; Curtis, D. E.; Lonie, D. C.; Vandermeersch, T.; Zurek, E.; Hutchison, G. R. Avogadro: An Advanced Semantic Chemical Editor, Visualization, and Analysis Platform. *J. Cheminform.* **2012**, *4* (1), 17. https://doi.org/10.1186/1758-2946-4-17.

(86) Takao, K.; Ikeda, Y. Structural Characterization and Reactivity of UO_2(Salophen)L and $[UO_2(Salophen)]_2$: Dimerization of UO_2(Salophen) Fragments in Noncoordinating Solvents (Salophen = *N,N*'-Disalicylidene-*o*-Phenylenediaminate, L = *N,N*-Dimethylformamide, Dimethyl Sulfoxide. *Inorg. Chem.* **2007**, *46* (5), 1550–1562. https://doi.org/10.1021/ic0611950.

(87) Hardwick, H. C.; Royal, D. S.; Helliwell, M.; Pope, S. J. A.; Ashton, L.; Goodacre, R.; Sharrad, C. A. Structural, Spectroscopic and Redox Properties of Uranyl Complexes with a Maleonitrile Containing Ligand. *Dalt. Trans.* **2011**, *40* (22), 5939. https://doi.org/10.1039/c0dt01580f.
